# Functional and Structural Diversity of Acyl-coA Binding Proteins in Oil Crops

**DOI:** 10.3389/fgene.2018.00182

**Published:** 2018-05-22

**Authors:** Nadia Raboanatahiry, Baoshan Wang, Longjiang Yu, Maoteng Li

**Affiliations:** ^1^Department of Biotechnology, College of Life Science and Technology, Huazhong University of Science and Technology, Wuhan, China; ^2^Hubei Key Laboratory of Economic Forest Germplasm Improvement and Resources Comprehensive Utilization, Hubei Collaborative Innovation Center for the Characteristic Resources Exploitation of Dabie Mountains, Huanggang Normal University, Huanggang, China; ^3^College of Life Science, Shandong Normal University, Jinan, China

**Keywords:** Acyl-coA binding protein, structure and function, paralogs, orthologs, evolution, oil crops

## Abstract

Diversities in structure and function of ACBP were discussed in this review. ACBP are important proteins that could transport newly synthesized fatty acid, activated into -coA, from plastid to endoplasmic reticulum, where oil in the form of triacylglycerol occurs. ACBP were detected in various animal and plants species, which indicated their importance in biological function. In fact, involvement of ACBP in important process such as lipid metabolism, regulation of enzyme and gene expression, and in response to plant stresses has been proven in several studies. In this review, findings on ACBP of 11 well-known oil crops were reviewed to comprehend diversity, comparative analyses on ACBP structure were made, and link between structure and function, tissue expression and subcellular location of ACBP were also observed. Incomplete reports in some species were mentioned, which might be encouraging to start or to perform deeper studies. Similar characteristics were found in paralogs ACBP, and orthologs ACBP had different functions, despite the high identity in amino acid sequence. At the end, it is confirmed that ortholog proteins could not necessarily display the same function, even from closely related species.

## Introduction

The acyl-coA binding proteins (ACBP) could bind, convey, and maintain intracellular acyl-coA pool (Rosendal et al., 1993; Knudsen et al., 1994; Rasmussen et al., 1994; Schjerling et al., 1996; Huang et al., 2005). High affinity exists between the acyl-coA binding domain of ACBP and the long-chain acyl-coA esters (12–22 carbons) (Pacovsky, 1996; Leung et al., 2006). ACBP are present in many species, indicating their great value in biological function (Knudsen et al., 1999; Faergeman et al., 2007). In fact, involvement of ACBP in biosynthesis of membrane, in regulation of enzyme activities and gene expression in lipid metabolism, in cellular signaling, in stress management, and disease resistance have been reported in several studies (Hunt and Alexson, 2002; Chen et al., 2008; Li et al., 2008; Oikari et al., 2008; Du et al., 2013b).

**Figure 1:**
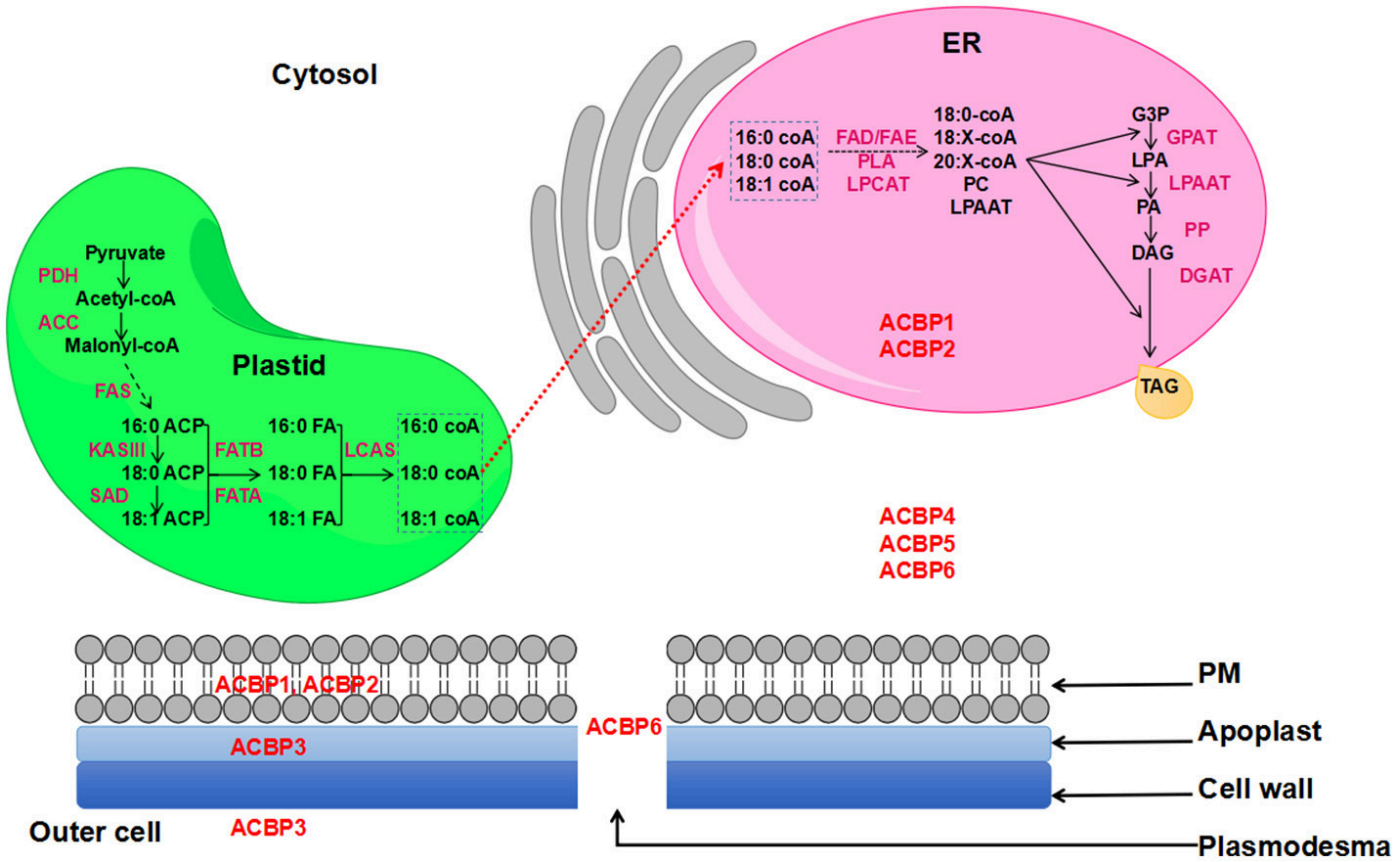
FA biosynthesis and TAG formation in *Arabidopsis thaliana*. ACBP are in red color and are placed according to their subcellular localization. Red dashed arrow indicates the non-vesicular transportation of *de-novo* synthesized FA to the ER involving ACBP.

Nowadays, demand in oil is increasing, and one strategy used to satisfy this demand is to transform plants via transgenic technology, so they could produce more oil. Also, transgenes are expected to have the ability of altering oil composition. In a former study, ACBP have proven their effectiveness in altering seed oil (Yurchenko et al., 2014). In fact, oil bodies are mainly constituted by triacylglycerol (TAG), which is an ester of glycerol and fatty acids (FA). Various plant organs and tissues contain TAG, including seeds, where TAG provides energy required for metabolism (Kaup et al., 2002). Formation of TAG occurs in the endoplasmic reticulum (ER) and FA are synthesized in the plastid. Several literatures gave comprehensive review illustrating FA biosynthesis and oil formation, as well as lipid transportation in plants, including the role of ACBP in lipid metabolism (Chapman and Ohlrogge, 2012; Li-Beisson et al., 2013; Hurlock et al., 2014). Actually, ACBP have been suggested to be the possible transporters of newly synthesized FA from plastid to the ER, prior to the formation of TAG (Chapman and Ohlrogge, 2012). A representation of FA biosynthesis and TAG formation in the model plant *Arabidopsis thaliana* is shown on Figure 1.

Current knowledge on ACBP in plant species mainly resulted from inquiries in *A. thaliana*. Certainly, many valuable findings proved the importance of these ACBP in lipid metabolism, in plant development, and in response to biotic and abiotic stress factors; however, roles of ACBPs in other plants have been poorly studied. Actually, efforts have been made to study ACBPs in other plants, referring to *A. thaliana*, in order to discover similar or new functions. Furthermore, the basic knowledge on their valuable function has given enough reason to highlight gene structure and evolutionary relationships within ACBP family, but also among plant species, which led us to compare and comprehend the structural and functional diversity in oil crops ACBP.

Oil crops are plants that are highly valued because of edible and industrial oils that they can provide from their seeds, fruits and nuts. The USDA reported that the most produced and consumed vegetable oils from years 2013/2014 to years 2017/2018 in the world were produced by eight oil crops: palm (*Areca* sp.), soybean (*Glycine max*), rapeseed (*Brassica napus*), sunflower (*Helianthus annuus*), peanut (*Arachis hypogaea*), cotton (*Gossypium hirsutum*), coconut (*Cocos nucifera*), and olive (*Olea europaea*), respectively (US Department of Agriculture; USDA Foreign Agricultural Service, 2017a,b In STATISTA—https://www.statista.com/statistics/263933/production-of-vegetable-oils-worldwide-since-2000/ and https://www.statista.com/statistics/263937/vegetable-oils-global-consumption/). In this review, ACBP from these important oil crops were reviewed, except for the peanut, coconut, and palm, of which ACBP could not be found on database. Besides, six other crops that could also provide oil, and of which ACBP are available on database, were added to this review, including rice (*Oryza sativa*), maize (*Zea mays*), tung tree (*Vernicia fordii*), physic nut (*Jatropha curcas*), turnip (*Brassica rapa*) and broccoli (*Brassica oleracea*). Note that *B. napus* was produced from hybridization of these two last species *B. rapa* and *B. oleracea*. Thus, in total, 11 well-known and important oil crops' ACBP were reviewed. Current findings were described, and incomplete investigations were pointed out, which might encourage to start or to add more researches. Additionally, conserved and separated functions in orthologs and paralogs ACBPs were discussed.

## Structure of ACBP

ACBP contain an acyl-coA binding domain that allows them to fulfill the function of acyl-coA transporters with high affinity (Rosendal et al., 1993; Leung et al., 2004, 2006). Three-dimensional structure of ACBP were reported in multiple classes of organism, such as in bovine (*Bos taurus*, Andersen and Poulsen, 1992; Kragelund et al., 1993), in *Plasmodium falciparum* (van Aalten et al., 2001), in yeast *Saccharomyces cerevisiae* (Teilum et al., 2005), and in human (*Homo sapiens*, Taskinen et al., 2007). In the past, we performed an *in-silico* analysis of ACBP structure in rapeseed (*B. napus*, Raboanatahiry et al., 2015a). In these above-cited reports, ACBP displayed a common alpha-helix shape. In bovine, the four alpha-helixes were held by hydrophobic interaction and showed an “up-down-down-up” direction: H1 (Glu4-Leu15), H2 (Asp21-Val36), H3 (Gly51-Lys62), and H4 (Ser65-Tyr84) (Andersen and Poulsen, 1992; Kragelund et al., 1993). The hydrophobic cleft between the second and third helixes protect the ω-end of the acyl-chain and the -coA part of the ligand from interactions with solvent (Faergeman et al., 2007). An overhang loop connected the second and third helixes and could be implicated in the capture of the ligand (Vallejo et al., 2009). In bovine ACBP (and in human also), amino acid residues Met-46, Leu-47, Phe-49 in that loop could preserve hydrophobicity. Also, hydrophobic residues Met-46 and Leu-47, and charged residues Lys-18 and Lys-50 were involved in ACBP-membrane interaction and acyl-coA extraction (Vallejo et al., 2009). In 1996, Engeseth et al. characterized ACBP in *A. thaliana*. They compared ACBP of *A. thaliana* with bovine and human ACBP and other group of plants. Fifteen conserved amino acid residues were found, which corresponded to Phe-7, Leu-27, Tyr-30, Lys-34, Glu-35, Ala-36, Gly-39, Pro-46, Gly-47, Lys-56, Trp-57, Asp-58, Trp-60, Ala-71, Tyr-75 in *A. thaliana* and *B. napus* (Engeseth et al., 1996). Later in 1999, Kragelund et al. compared ACBP in animal, yeast and plants including *A. thaliana* and *B. napus*. The ligand binding site of the protein could be divided into three subsites destined to the acyl-part of the ligand, to the adenine ring and to the 3′-phosphate of the ligand, respectively (Kragelund et al., 1999). The 3′-phosphate (CoA) could cooperate with ACBP through a network of two salt bridges to Lys-34 and Lys-56, and a hydrogen bond to Tyr-30 (Faergeman et al., 2007). The adenine rings maintained a non-polar interaction with aromatic rings of Tyr-30, Tyr-75, and Phe-7 (Chye et al., 2000), and the non-polar ω-end of the acyl chain made several attractions to the non-polar side chains of Leu-27 and Ala-55. In 2011, Xiao and Chye aligned the acyl-coA binding domains of ACBP from different species of animal, yeast, and plant including *A. thaliana* and *B. napus*. Nineteen amino acid residues were suggested to be conserved in all concerned species. The potential binding sites for acyl-CoA esters implied five amino acid residues which in AtACBP6 corresponded to Phe-7, Tyr-30, Lys-34, Lys-56, and Tyr-75. The YKQA and KWDAW motifs, essential in binding acyl-CoA esters (as suggested by Kragelund et al., 1993) were conserved in all species (Xiao and Chye, 2011). The acyl-coA binding mechanism was studied: one acyl-coA ester could bind to a single binding site by cooperativity. The bond is strong, with high affinity (Rosendal et al., 1993; Gossett et al., 1996). This binding affinity could occur in a low micromolar range (0.1–20 μM) in animal and plant ACBP (Rasmussen et al., 1990; Gossett et al., 1996; Leung et al., 2006). The affinity to bind acyl-coA ester depended greatly on the length of the acyl chain and the number of double bonds in acyl-CoA, with a clear preference for acyl-coA esters that have more than eight carbon atoms, but yet those with 12–20 carbon atoms were, by far, most preferred (Faergeman et al., 1996; Pacovsky, 1996).

## ACBP in the model plant *A. thaliana*

As mentioned above, most of reports on plant ACBP are about *A. thaliana* ACBP (AtACBP), which allowed to enrich our knowledge on ACBP functions. Thus, it is worth to sum up findings on AtACBP before reviewing those of the oil crops. So, AtACBP are divided into four separate classes according to their structure, their binding affinity, their subcellular localization, their expression and their function.

### The small AtACBP

The small AtACBP, widely known as AtACBP6, contains 92 amino acids, with relative molecular mass of 10.4 kDa. Small AtACBP was found to be localized in the cytosol (Xiao and Chye, 2009), and in the plasmodesmata where it interacted with plasmodesma protein PDLP8 (Ye et al., 2017a). AtACBP6 could bind better 16:0-CoA and 18:2-CoA, rather than 18:1-CoA and 18:3-CoA (Xiao and Chye, 2011; Hsiao et al., 2014). In fact, AtACBP6 was involved in intracellular binding and shipping of PC in plant phospholipid metabolism (Chen et al., 2008). Small AtACBP were demonstrated to affect fatty acid composition in a study performed by Enikeev and Mishutina (2005): in this study high and low erucic acid rapeseed cultivars were transformed small AtACBP construct. Levels of monounsaturated fatty acids (20:1 and 22:1) decreased in the high erucic acid cultivar transformed with sense construct, whereas those transformed with anti-sense construct displayed an increase of 22:1 in the seed oil. Otherwise, AtACBP6 was shown to be expressed in all tissues and enhanced freezing tolerance to the host (Chen et al., 2008; Chye et al., 2010; Liao et al., 2014). Its presence in the seeds and in phloem were also demonstrated (Hsiao et al., 2014; Ye et al., 2016), and its role in systemic trafficking, and jasmonates and/or its derivatives increasing content in the sieve tubes were recently reported (Ye et al., 2016). Mutants of *acbp4/acbp5/acbp6* in *AtACBP6* produced important accumulation of 18:1-coA in the embryos and considerable decrease in seed weight (Hsiao et al., 2014).

### The ankyrin repeats AtACBP

The ankyrin repeats AtACBPareAtACBP1 and AtACBP2 that share 76.9% of identity. AtACBP1 contain 338 amino acids (37.5 kDa) and AtACBP2 have 354 amino acids (38.5 kDa). Apart from the acyl-coA binding domain, a N-terminal membrane-associated domain and a C-terminal ankyrin repeats domain were found in their structure (Leung et al., 2004). AtACBP1 and AtACBP2 could bind C18:2-coA and C18:3-coA esters (Gao et al., 2009). Especially, AtACBP1 could bind PA (Du et al., 2010). Recombinants AtACBP1 were found to bind C18:1-coA (Chye, 1998), they were later demonstrated to bind PA and PC (Du et al., 2013a), whereas rAtACBP2 could bind lysophospholipids (LPL) and LPC (Gao et al., 2010), and PC (Chen et al., 2010). The N-terminal membrane associated domain targeted them to their common localization, the ER and the plasma membrane (Li and Chye, 2003; Xiao and Chye, 2009). Expressions of proteins were observed in all tissues but AtACBP1 expression level was higher in seeds and siliques (Chye et al., 1999), whereas AtACBP2 was highly expressed in roots, stems and flowers (Li and Chye, 2003). The ankyrin repeats AtACBP could be involved in lipid metabolism and plant stress response. AtACBP1 and AtACBP2 are membrane-associated proteins, involved in acyl-CoA transfer and metabolism (Li and Chye, 2003), they were thought to be protein-protein interactions mediators in responses to heavy-metal stress (Xiao et al., 2008; Gao et al., 2009, 2010), but no obvious role was found in embryo development (Chen et al., 2010). Particularly, AtACBP1 was involved in seed lipid metabolism in presence of acyl-CoA esters (Chye, 1998), and could maintain a membrane-associated acyl pool in inter-membrane lipid transport from the ER to the plasma membrane via vesicles. Possible roles in cuticle and cutin formation (Chye et al., 1999), in Pb (II) tolerance and accumulation in shoots (Xiao et al., 2008), in epicuticular wax deposition (Xue et al., 2014), in decreasing freezing tolerance (Du et al., 2010), and in abscisic acid elevation (Du et al., 2013a) were suggested. Recently, it was demonstrated that AtACBP1 negatively modulated sterol synthesis during embryogenesis, and controlled the metabolism of FAs and sterols influencing cellular signaling (Lung et al., 2017). However, AtACBP2 was reported to interact with the *A. thaliana* ethylene-responsive element-binding protein (AtEBP) and farnesylated protein 6 (AtFP6) through ankyrin repeat (Li and Chye, 2004). AtACBP2 could bind Pb(II), Cd(II), and Cu(II), and might be implicated in post-stress membrane repair (Gao et al., 2009). Additionally, via LPC and LPL binding, AtACBP2 could sustain LPC degradation in response to Cd-induced oxidative stress (Gao et al., 2010). Also, AtACBP2 could promote ABA signaling in germination, seedling development, and drought response (Du et al., 2013b).

### The large AtACBP

The large AtACBP is commonly known as AtACBP3, it contains 362 amino acids (39.3 kDa). A N-terminal membrane-associated domain was also seen in AtACBP3 (Xiao et al., 2010), but it had been found in outer cell (Leung et al., 2006; Xiao and Chye, 2009) and in the apoplast (Xiao and Chye, 2011). AtACBP3had high affinity for binding arachidonyl-coA (C20:4) (Leung et al., 2006), and rAtACBP3 could bind PC, phosphatidylethanolamine (PE), and unsaturated acyl-CoA (Xiao et al., 2010). It was found that AtACBP3 was expressed in all tissues but expression in siliques and young shoots were higher (Xiao and Chye, 2009). Expression was induced by darkness and down-regulated in extended light. Otherwise, AtACBP3 was involved in many biological functions as plant defense signaling during fungal infection (Choi et al., 1994), circadian regulation (Zheng et al., 2012), and response to hypoxia (Xie et al., 2015). Another study demonstrated that AtACBP3 could indorse starvation and age-dependent leaf senescence, and increased PE, PA, LPA, and arabidopsides level (Xiao et al., 2010). Moreover, AtACBP3 was reported to be essential for maintaining normal lipid level and participated in the lipid fluctuation between the prokaryotic and eukaryotic pathways; similar to AtACBP4 and AtACBP6, AtACBP3 was defined to be vital for cuticle development and for defense against microbial pathogens (Xia et al., 2012). A recent study reported the presence of AtACBP3 in companion cells, sieve elements and the apoplast of phloem and its role in jasmonate production in response to injuries, the profile of fatty acid content was also affected by the diminution of AtACBP3, i.e., lower C18:2 and C18:3 level (Hu et al., 2018).

### The kelch motifs AtACBP

The kelch motifs AtACBP are AtACBP4 and AtACBP5 that shares 81.4% of identity: AtACBP4 contains 668 amino acids (73.3 kDa) and AtACBP5 has 648 amino acids (71 kDa), they both conserve five kelch motifs apart from the acyl-coA binding domain found on their structure (Leung et al., 2004). These kelch motif proteins could bind C18:1-coAand PC (Leung et al., 2004; Xiao et al., 2009), and they were found in the cytosol (Chen et al., 2008; Xiao et al., 2008; Xiao and Chye, 2009; Ye et al., 2017b). Their expression in all tissues had been demonstrated but the level was higher in roots for AtACBP4 and in young shoots and mature leaves for AtACBP5 (Li et al., 2008). In seeds, expression of AtACBP4 occurred in early embryogenesis and AtACBP5 were expressed later (Hsiao et al., 2014). In a recent study, AtACBP4 and AtACBP5 were inversely expressed in anther development, with early expression of AtACBP5 in microspores and tapetal (before stage 9, and absent at stage 10) and later expression of AtACBP4 in pollen and endothecium (from stage 10) (Ye et al., 2017b). Concerning the role of these kelch motifs AtACBP, they were reported to be actively involved in plant lipid metabolism and defense reaction. They could satisfy demands of lipids in plant cells (Xiao et al., 2009). Thus, AtACBP4 was suggested to act on the biosynthesis of membrane lipids (galactolipids and phospholipids) (Xiao et al., 2008). Similar to AtACBP2, AtACBP4 interacted with AtEBP, related to AtEBP-mediated defense possibly via ethylene and/or jasmonate signaling (Li et al., 2008). Though, AtACBP5 cooperated in seed and pollen development (Hsiao et al., 2014). The ability of these kelch motif ACBP to accumulate Pb(II) in roots as response to stress, were also demonstrated (Du et al., 2015). Additionally, Ye et al. (2017b) reported that significant increase in C29-alkanes (wax) was observed in flower buds of mutants *acbp4* and *acbp4acbp5*, accompanying by an increase in C18:2, but decrease in C18:0. They also pointed out that AtACBP5 and AtACBP4 expression increased in *acbp4* and *acbp5*, respectively, and a decrease of ɑ-amylose content was only seen in *acbp5*. A pollen-specific cis-acting element POLLEN1 (AGAAA) at AtACBP4 was also reported.

## Synopsis on fourteen oil crops and their ACBP

Breeding history of species is reflected on their genetic profile, which is also affected by environment fluctuation. Genes work together to support their development and adaption (nutriment assimilation, stress management). Complex mechanisms are coordinated first in molecular level and then within cells, and gene products support species for survival, but this also affects their productivity. In the following paragraphs, major findings on ACBP in oil crops were assembled, the aim was to recognize their divergence in functions despite their similar belonging to the same family. To the best of our knowledge, very few studies have been done on ACBP in some species, and no report of ACBP function were found in maize (*Z. mays*), soybean (*G. max*), peanut (*A. hypogaea*), coconut (*C. nucifera*), palm (*Areca* sp.), olive (*O. europea*), turnip (*B. rapa*), and broccoli (*B. oleracea*). Therefore, this synopsis might encourage to initiate or to perform further studies.

### Rapeseed (*B. napus*)

*B. napus* ACBP (BnACBP) was first isolated by Hills et al. (1994), six copies were found, of which three copies each were inherited from *B. rapa* and *B. oleracea*, respectively. Protein contained 92 amino acids which displayed high conservation with yeast and human ACBP. Small ACBP of *B. napus* weighted 10 kDa and showed 84% amino acid sequence identity to *A. thaliana* AtACBP6 (Hills et al., 1994; Engeseth et al., 1996). Hills et al. (1994) reported these proteins to be strongly expressed in developing embryo, flowers and cotyledons of seedlings, but lower expression were found in roots and leaves of *B. napus*, which suggest their implication in seed development and storage of lipids. Observation of ACBP fluctuation level during embryo development for adaption of acyl-coA intracellular levels resulted in findings that highest expression occurred simultaneously the peak of TAG biosynthesis (Engeseth et al., 1996; Brown et al., 1998). Moreover, Brown et al. (1998) demonstrated the ability of 10 kDa BnACBP to bind C16:0-coA and C18:1-coA. It was also reported that recombinant BnACBP (rBnACBP) could improve LPAAT and GPAT activities (Brown et al., 1998, 2002). Diminution of glucose-6-phosphate (G6P) inhibition, caused by high concentration of LCAS in the plastid, was observed due to BnACBP activity (Fox et al., 2000). BnACBP isolated from embryonic plastid could bind long chain acyl-coA, and in presence of coA and BnACBP, carbon from glucose-6-phosphate (G6P) was incorporated into FA and converted into acyl-coA, prior to their exportation from plastid; however, absence of coA or bovine serum albumin (BSA) altered the rate of synthesis and/or its end products, independently from presence of BnACBP due to its affinity for binding of exported acyl-coA (Johnson et al., 2000, 2002).

Overexpression of these 10 kDa BnACBP in *A. thaliana* seeds resulted in increased level of C18:2 and C18:3, and rBnACBP enhanced PC and was exposed as important for LPCAT activity in the transfer of acyl group from PC into acyl-coA (Yurchenko et al., 2009). Moreover, test on effect of rBnACBP concentration on microsomal DGAT activity and TAG level were also made, and it was reported that at a ratio of 0.33 in rBnACBP:acyl-CoA, DGAT activity increased by 20% and at 1.66, TAG level decreased as DGAT activity weaken. rBnACBP had few influence on DGAT at ratio of 0.6–0.8, in a ratio equal to 1, activity of DGAT was inhibited (Yurchenko et al., 2009). Furthermore, this 10 kDa BnACBP was shown to effectively alter seed oil acyl-coA pool and acyl composition. In fact, increase of C18:2 but decrease of C20:1 were observed in acyl-coA pool and seed oil of *A. thaliana* at maturity stage, which were not often correlated. The same study revealed the activity of BnACBP in ER, where they exhibited similar activity as in cytosolic space concerning the FA profile alteration, but showed an obvious decrease of C18:3 in both oil and acyl-coA pool (Yurchenko et al., 2014).

Since previous studies focused only on 10 kDa BnACBP, we assessed *in-silico* studies to identify and characterize other classes of BnACBP, including ankyrin repeats, large and kelch motifs proteins, using AtACBP as model, they were named BnACBP1 to BnACBP6, similar to AtACBP; then, we cloned homologs kelch motif ACBP in *B. napus* (BnACBP4 and BnACBP5) and found eight copies of which structure was very close to AtACBP4 and AtACBP5 (Raboanatahiry et al., 2015b). Structure of BnACBP was compared to AtACBP, including conserved residues, conserved domains, secondary and tertiary structures. Structures were less or more similar to those of *A. thaliana*. Eight amino acid residues were identical in acyl-coA binding domains of all BnACBP, and they displayed alpha-helix shape by 3D visualization (Raboanatahiry et al., 2015a). Lately, Ling et al. (2017) studied a homolog of AtACBP1, named BnACBP1-like in *B. napus*, and suggested its involvement in early leaf senescence through induction of jasmonate and oxylipin signal transduction, in contrast to AtACBP1 which could not induce leaf senescence in another study made by Lung and Chye (2016). It was also suggested that BnACBP1-like might enhance C18:3 content in plastid, as well as PC/PA exchange (Ling et al., 2017). Thus, despite the fact that *A. thaliana* and *B. napus* are closely related, functions could not be the same, this is why more attention should be focused in studying function of ACBP in *B. napus*.

### Rice (*O. sativa*)

An abundant protein expressed in rice phloem was isolated and characterized and it showed high similarity with AtACBP and BnACBP 10 kDa proteins. This hypothetical rice ACBP was demonstrated to be one of major proteins present in phloem-sap. Mature protein from sieve tubes lacked of methionine in its N-terminal part which might have been removed after translation. Sieve tube in various plants, such as *C. nucifera, Cucurbita maxima*, and *B. napus* also contained ACBP (Suzui et al., 2006). A complete characterization of rice ACBP family (OsACBP) including six gene members was performed by Meng et al. (2011). Their study reported relationship and comparison of OsACBP with other ACBP from 16 land plants. Like AtACBP, OsACBP could be subdivided into four classes: OsACBP1, OsACBP2, and OsACBP3 were small ACBP, OsACBP4 was ankyrin repeats ACBP, OsACBP5 was large ACBP, and OsACBP6 was kelch motif ACBP. These OsACBP displayed difference in binding affinities, in spatial expression and in implication in plant stress response. In fact, all rOsACBP could bind C18:3-coA, but in addition, rOsACBP1 could bind C18:1-coA, whereas rOsACBP4 could bind C18:2-coA. Expression analyses of OsACBP revealed their presence in seed, leaf, stem, and root. However, the expression level varied with seed development: higher expression of all mRNA in leaf, moderate expression in root, and lower expression in stem at germination stage. During seed development, constant expression of OsACBP1 was found in anthesis, milk, and soft dough stages, whereas peaked expression were observed in OsACBP2 at dough stages, and in OsACBP3 and OsACBP4 at anthesis stage. OsACBP6 displayed its lowest expression at milk stage. Though, high expression of OsACBP5 was observed during the entire phase of reproduction.

Besides, OsACBP responded differently to stress, i.e., drought and salinity, cold, wound, and pathogen attack. Drought and high salinity treatments did not affect OsACBP1, OsACBP2, and OsACBP3, then OsACBP4 was induced by drought, and with OsACBP5, they both peaked at 12 h after salt treatment which persisted at relatively high levels. Under cold treatment for 12 h, all OsACBP were suppressed and then came back to normal level at 24 h, except for OsACBP6 of which no obvious influence was found up to 12 h but lower level was detected at 24 h. OsACBP5 and OsACBP6 were induced by wound, peaks were observed at 0.5 h following by a decreased expression level. While exposed to fungus infection, OsACBP5 was induced at the expense of OsACBP1, OsACBP2, OsACBP3, and OsACBP4 (Meng et al., 2011). While all gene members of ACBP family were discovered in rice, further inquiries were made, and revealed that OsACBP1 and OsACBP2 were localized in the cytosol, OsACBP4 and OsACBP5 were found in endoplasmic reticulum, OsACBP6 was located in the peroxisomes, and OsACBP3 was found in multiple subcellular location (Meng and Chye, 2014; Meng et al., 2014). Moreover, overexpression of OsACBP6 in *peroxisomal abc transporter1* mutant recovered expression of wound-induced VSP1 and promoted production of jasmonate (Meng et al., 2014).

In a study reported by Guo et al. (2017), small OsACBP2 could bind unsaturated coA esters with high affinity than OsACBP. While elucidating OsACBP structure, common helix shape of ACBP was revealed, but difference between OsACBP1 and OsACBP2 resided at helix 3, which was suggested to be the cause of ligand binding affinity between them. These various reports on OsACBP certainly enriched our knowledge, but in case of gene knock-out, which findings could be expected, would they confirm previously cited discoveries? Deeper studies are worth to be added.

### ACBP in sunflower (*H. annuus*), in cotton (*G. hirsutum*), in tung tree (*V. fordii*), and physic nut (*J. curcas*)

So far as we know, only few reports are currently available on ACBP in these four oil crops. Fortunately, important evidence could be gathered and might be useful as biotechnological tool for adjustment of valuable traits. Nevertheless, it is essential to perform supplementary studies to take advantage of eventual beneficial functions.

In sunflower, HaACBP6 is a homolog of AtACBP6 and BnACBP6 (75 and 78% of identity, respectively). Structure, subcellular location and function were studied: this protein had molecular weight of 10.86 kDa, and as predicted in BnACBP and other ACBP, it displayed four-helix shape. HaACBP6 was expressed in vegetative tissues and strong expressions were observed in developing seeds and germinating cotyledons. This cytosolic protein was demonstrated to have high affinity for binding C16:0-coA, C18:0-coA, and C18:1-coA, rather than C18:2-coA, and its recombinant rHaACBP6 had affinity to bind dipalmitoyl-PC, dioleoyl-PC, and dilinoleoyl-PC, but no affinity for LPC, PA, and LPA were found (Aznar-Moreno et al., 2016).

In cotton, 21 ACPBP genes that could be subdivided into four classes were identified, according to AtACBP, they were named GhACBP1 to GhACBP6. Analysis revealed high level expression of GhACBP6 in all tissues, whereas the expression of GhACBP1 in developing ovules, GhACBP3 in flower, petal, stamen, and in developing secondary wall of fiber, and GhACBP4 in developing fiber were observed. However, no expression of GhACBP2 and GhACBP5 was detected in observed tissues. These GhACBP were exposed to stress treatments, and results revealed that GhACBP1, GhACBP3, and GhACBP6 were significantly induced by drought, salt, and low and high temperature treatments. Moreover, GhACBP3 and GhACBP6 were significantly induced by hydrogen peroxide, salicylic acid, jasmonate, abscisic acid, and ethylene. GhACBP3 and GhACBP6 down-regulation weaken drought and salt tolerances in plant, and reduced plant height, superoxide dismutase, and peroxidase activities, but improved malondialdehyde content (Qin et al., 2016).

Two genes belonging to class III ACBP (large ACBP) were characterized in tung tree (VfACBP3). They differed in structure notably in sequence, length, exon/intron architecture, and three-dimensional shape. VfACBP3 displayed similar expression in flowers and seed development, but one VfACBP3 (named VfACBP3A) was highly expressed in very young seeds, and stronger expression was also observed in leaves, compared to VfACBP3B. Then, 3D structure was predicted, and exposed the four alpha-helixes shapes of common ACBP in C-terminal side of protein, but two alpha-helixes were in addition in N-terminal side of proteins. Especially for VfACBP3B, C-terminal extension contained one long helix and two short helices. Subcellular localization inquiry resulted in the presence of these VfACBP3 in the ER, and it was reported that VfACBP3 had affinity for binding C18:1-coA and C20:4-coA (Pastor et al., 2013).

Limited information is available about *J. curcas* ACBP (JcACBP) by now. It was cloned and characterized; the protein contained 92 amino acids and was presumed to have a molecular mass of 10.30 kDa. JcACBP displayed 96% identity with *V. fordii* VfACBP. Significant expression of JcACBP was observed in different organ, but the highest detected was in fruit, where expression level was in accordance with lipid accumulation (Wen et al., 2014).

Finally, ACBP in oil crops had different characteristics, but this might be related to their structure. However, it is not clear how ACBP with same protein domain had different characteristic, and those which had different protein domain had similar characteristic, for instance in the above-mentioned study reported by Ling et al. (2017), BnACBP1 and AtACBP1 displayed different function. Thus, ACBP structure in oil crops should be compared, for further elucidations.

## Comparative analysis of ACBP structure in oil crops

Previously, we summarized essential findings on ACBP in oil crops, including protein size, binding affinity, subcellular location, expression, and biological functions. It was clear that ACBP displayed different characteristics, even for those which belonged to the same class. Protein function is linked to its structure, and since these diverse ACBP were classified according to their domain structure, in the following section, we compared ACBP structure of these oil crops in order to comprehend diversity.

### Identification of ACBP

Identification of oil crops ACBP were based on homology with *A. thaliana* ACBP. First, we acquired ACBP in *A. thaliana* from TAIR website (www.arabidopsis.org), then Blastp tool of NCBI (www.ncbi.nlm.nih.gov) was used, with protein sequences of *A. thaliana* as model to find homologous proteins in each of the crop species. To make sure each protein had the acyl-coA binding domain (ACBD), protein domain analysis for each protein sequence was performed (results shown below). All accessions found in NCBI database, which had sequence similarity more than 60% with *A. thaliana* ACBP and had acyl-coA binding domain in their structure, were taken in this study. Thus, a total of 180 ACBP proteins for 11 oil crop species could be found in our analysis (Supplementary Table 1). *B. napus* had a total of 45 different accessions of ACBP: six small proteins (90–95 Aa), seven ankyrin repeats proteins (339–352 Aa), 16 large proteins (208–381 Aa), and 16 kelch motifs proteins (254–670 Aa). In our previous study, 20 copies of ACBP were identified in *B. napus*, we cloned eight copies of kelch motif ACBPs from high oil content rapeseed (about 50% of oil; Raboanatahiry et al., 2015b). Apart from these 28 copies (20 identified in *Darmor-bzh*, 8 cloned in our laboratory), NCBI database conserves sequence from other cultivars, such as *Zhongshuang11. G. hirsutum* had 24 ACBP accessions: two accessions for each of small (88–89 Aa) and ankyrin repeats proteins (367 Aa, each), 12 large proteins (203–342 Aa), and eight kelch motifs proteins (650–679 Aa). In *B. rapa* and *B. oleracea*, the same numbers of accessions in each class of ACBP were found: three small ACBP, each (90 and 92 Aa in *B. rapa*, and 92 and 93 Aa in *B. oleracea*), two ankyrin repeats ACBP, each (341 and 351 Aa in *B. rapa*, and 340 and 351 Aa in *B. oleracea*), eight large ACBP, each (208–381 Aa in *B. rapa*, and 109–385 Aa in *B. oleracea*), and seven kelch motifs ACBP, each (665–668 Aa in *B. rapa*, and 665–671 Aa in *B. oleracea*). Thus, each of *B. rapa* and *B. oleracea* had a total of 20 ACBP accessions found in NCBI database. *G. max* had two accessions each for ankyrin repeats proteins (346–354 Aa) and large proteins (294–408 Aa), and seven kelch motifs proteins (575–663 Aa), which made a total of 11 accessions of ACBP. *H. annuus* had 10 accessions of ACBP: two accessions of small (90 Aa, each) and ankyrin repeats proteins (329–349 Aa), and three accessions for each of large (252–371 Aa) and kelch motifs proteins (658–662 Aa). For *J. curcas*, a total of six ACBP accessions were found: two accessions for each of small (92 Aa, each) and large proteins (274–387 Aa), and one accession for each of ankyrin repeats (366 Aa) and kelch motifs proteins (673 Aa). *O. europaea* had a total of nine accessions of ACBP: two accessions each for small and ankyrin repeats ACBP, with length of 88 and 96 Aa in small ACBP, and 314 and 357 Aa in ankyrin repeats ACBP. One large ACBP was found with 260 Aa, and four accessions of kelch motifs ACBP (625–677 Aa). *V. fordii* had only three ACBP accessions with one accession each of small (91 Aa), large (375 Aa), and kelch motifs proteins (669 Aa). Finally, for the monocots*, O. sativa* had a total of 10 ACBP accessions: four small proteins (91–155 Aa), two ankyrin repeats proteins (334–336 Aa), one large proteins (562 AA), and three kelch motifs proteins (536–656 Aa), and *Z. mays* had three accessions each for small (89–106 Aa) and large proteins (166–537 Aa), five ankyrin repeats proteins (267–330 Aa), and 11 kelch motifs proteins (421–783 Aa), for a total of 22 ACBP accessions. Note that small ACBP were missing in *G. max*, and ankyrin repeats were also absent in *V. fordii*. Besides, some small ACBP were much larger or much shorter compared to *A. thaliana* ACBP, but their sequence identity and their phylogenetic position (results shown below) led us to classify them as small or large ACBP. For example, Os03g0576600 (155 Aa) was classified as small ACBP in *O. sativa*, since it had 75% identity with *A. thaliana* small ACBP AT1G31812, against 34% with the large ACBP AT4G24230 (inquiry using Blastp of NCBI), also in the phylogenetic tree, Os03g0576600 was clustered in the small ACBP group, our analysis matched with reported by Meng et al. (2011), of which that 155 Aa ACBP (OsACBP3) was homologous to AtACBP6.

**Figure 2:**
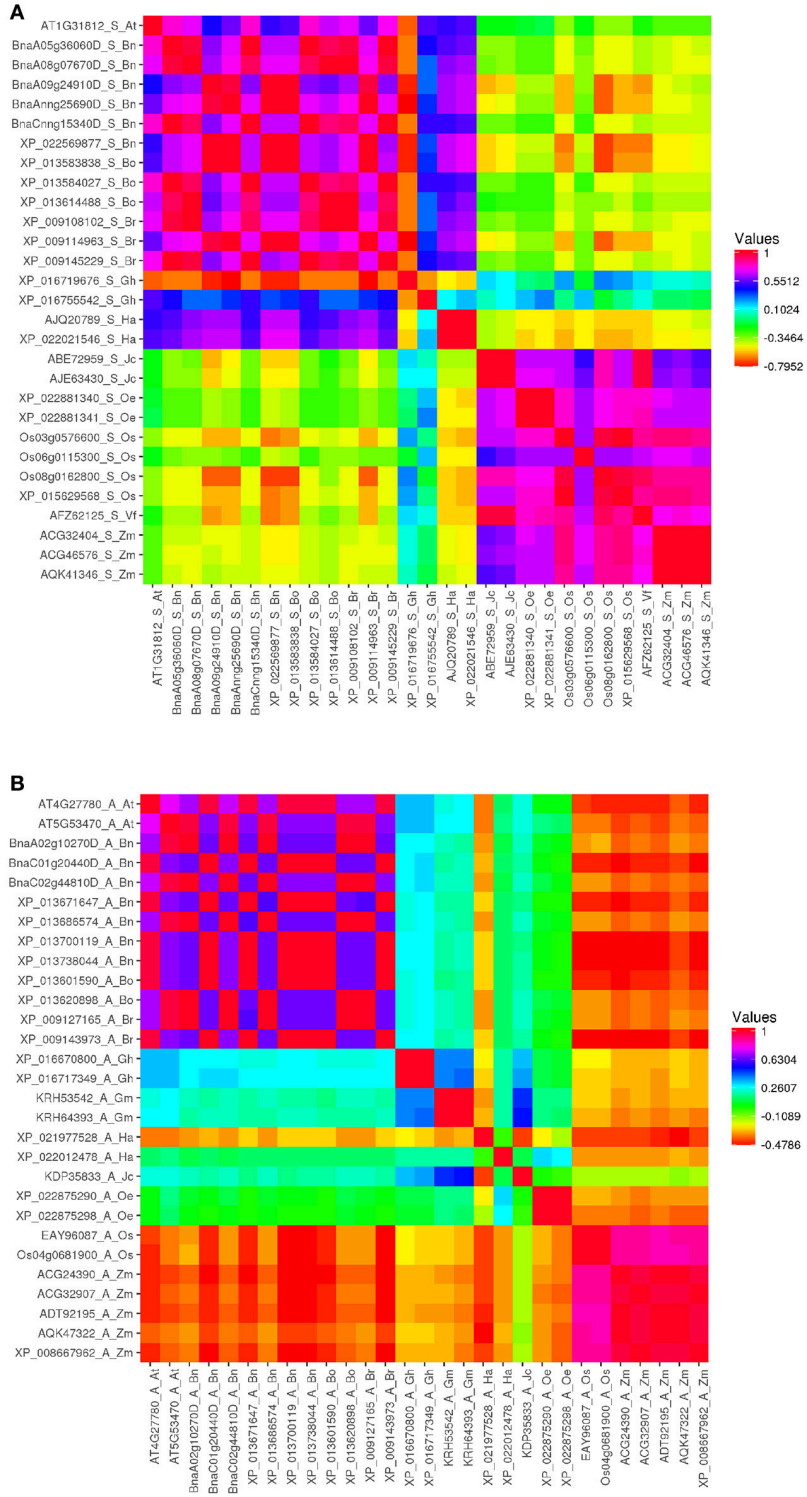
Heat map depicting amino acid sequence identity of oil crops ACBP. **(A)** Small ACBP, **(B)** Ankyrin repeats ACBP, large and kelch motifs ACBP are in Supplementary Figure 1. The map was generated by Heatmapper (Babicki et al., 2016), with Pearson's correlation matrix calculation. Color intensity change with distance value as indicated.

For deeper observation, amino acid sequence identity was measured with Vector NTI software (Supplementary Table 2). Pairwise correlation analysis was performed between these proteins, by using Heatmapper (www2.heatmapper.ca/; Babicki et al., 2016), then heat maps of each class of ACBP in oil crops were generated (Figure 2, Supplementary Figure 1). It was predictable that ACBP of same species or same family (*B. napus, B. oleracea*, and *B. rapa* both belong to *Brassicaceae* family) or same clade (monocots *O. sativa* and *Z. mays*) displayed higher identity compared to others. However, it was surprising that amino acid sequence identity of numerous large ACBP were considerably lower (<40%) even within same species, family or clade. In small ACBP class, identity between *J. curcas, V. fordii*, and the monocots were much higher, as in *H. annuus* and *G. hirsutum* (XP_016755542) which displayed moderately higher identity with *Brassicaceae* species (more than 76%). Heat map of ankyrin repeats and kelch motifs ACBP displayed nearly similar profile, *Brassicaceae* species displayed moderate identity value to slightly higher identity with the other eudicots (about 40–65% in ankyrin repeats ACBP and 65–75% in kelch motifs ACBP), with relatively lower identity with monocots.

ACBP were found in these 11 oil crops species which confirmed that ACBP are vital in biological function, and existed before plants speciation (Faergeman et al., 2007; Meng et al., 2011). Moreover, many copies were found in one ACBP class which might be due to duplication events, but multiple accessions were much available which might be the resultant of protein sequencing from different genotypes, reflecting copy number variation in different genotype, which increased the number of ACBP detected in these plants. However, some species lacked some class of ACBP as mentioned above; these missing ACBP might be lost during evolution. Therefore, 186 ACBP accessions, including six ACBP of *A. thaliana*, would be subjected to the next analyses.

### Phylogenetic relationship of oil crops based on ACBP structure

**Figure 3:**
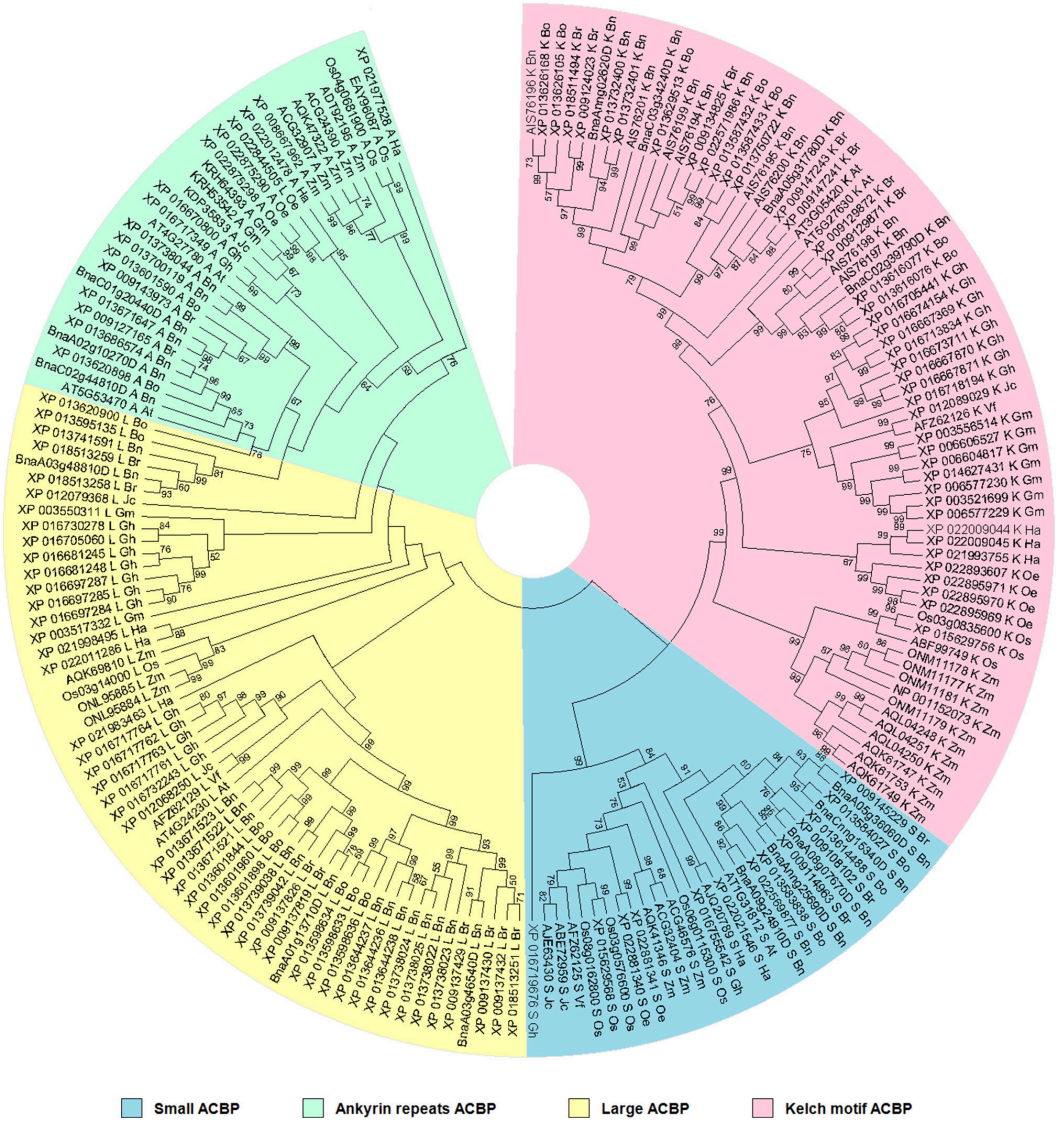
Phylogenetic relationships of oil crops, based on ACBP. The tree was inferred using the Neighbor-Joining method (Saitou and Nei, 1987). The evolutionary distances were computed using the p-distance method (Nei and Kumar, 2000) and are in the units of the number of amino acid differences per site. The analysis involved 186 amino acid sequences. ACBP families were clustered into four classes, indicated by different colors. Evolutionary analyses were conducted in MEGA7 (Kumar et al., 2016).

Phylogenetic relationship was analyzed to make a statement about resemblance level and affiliation in ACBP in oil crops. This is important to understand similarity or divergence in function of proteins based on their structure, but also might give a clue about function of ACBP in species which have not been defined yet. Therefore, phylogenetic tree was built based on structure using Neighbor-joining methods (Saitou and Nei, 1987; Nei and Kumar, 2000; Figure 3). Four major clusters separated the tree, according to the four different domain structures of ACBP. The topology of the tree was almost uniform: monocots species (*O. sativa* and *Z. mays*) were gathered side to side and diverged separately from the other species, as well as the *Brassicaceae* tribe *(A. thaliana* and *B. napus)*. Also, proteins of the same species were clustered, except for large ACBP of *O. europaea* (XP_022844505) which was misplaced in the ankyrin repeats clade. Each cluster of the tree were well and moderately supported (BS = 99% for small and kelch motifs ACBP, and BS = 76% for ankyrin repeats ACBP), except for the large proteins clade which lacked bootstrap support (BS < 50%).

Evolutionary histories of proteins and ACBP have been well discussed in the past (Apic et al., 2001; Ponting and Russell, 2002; Burton et al., 2005; Björklund et al., 2006; Vogel and Morea, 2006; Itoh et al., 2007; Meng et al., 2011; Raboanatahiry et al., 2015b). To sum up, ACBP derived from a common ancestor, and they became larger with the evolution. The small ACBP appeared before the other classes of ACBP, which were much larger. Because of protein domains which have independent evolution (duplication, insertion, deletion, recombination), new protein with multiple domains might appear, such as ankyrin repeats and kelch motifs ACBP. Expansion and functional diversity of ACBP might be resulted from evolution of proteins.

The aim of this analysis was to recognize the similarity and difference of ACBP structure in oil crops in order to compare their functions. Thus, referring to the tree, ACBP of same species or same family were grouped, which possibly indicated much similar in function or characteristics (expression and subcellular location) between them. Groups which were well supported, indicated that there were much more amino acid sequence similarities between them and they likely have similar functions, as for *B. rapa, B. oleracea*, and *B. napus* which were often supported by very good bootstrap value (BS > 90%). This is not much amazing as in distantly related species such as *V. fordii* and *J. curcas*, but still had good bootstrap value, as in one large well supported assembly (AFZ62129 and XP_012068250, respectively, with BS value of 99%), they might have closely similar structures and could be expected to have similar functions. Amino acid sequence alignment revealed more 12 amino acids presents in *J. curcas* (XP_012068250, 387 Aa) compared to *V. fordii* (AFZ62129, 375 Aa), they shared 69% amino acid sequence identity. Deeper explanations could be obtained with the next analysis, which focused more on ACBP protein domain architecture.

### Protein domain of ACBP

**Figure 4:**
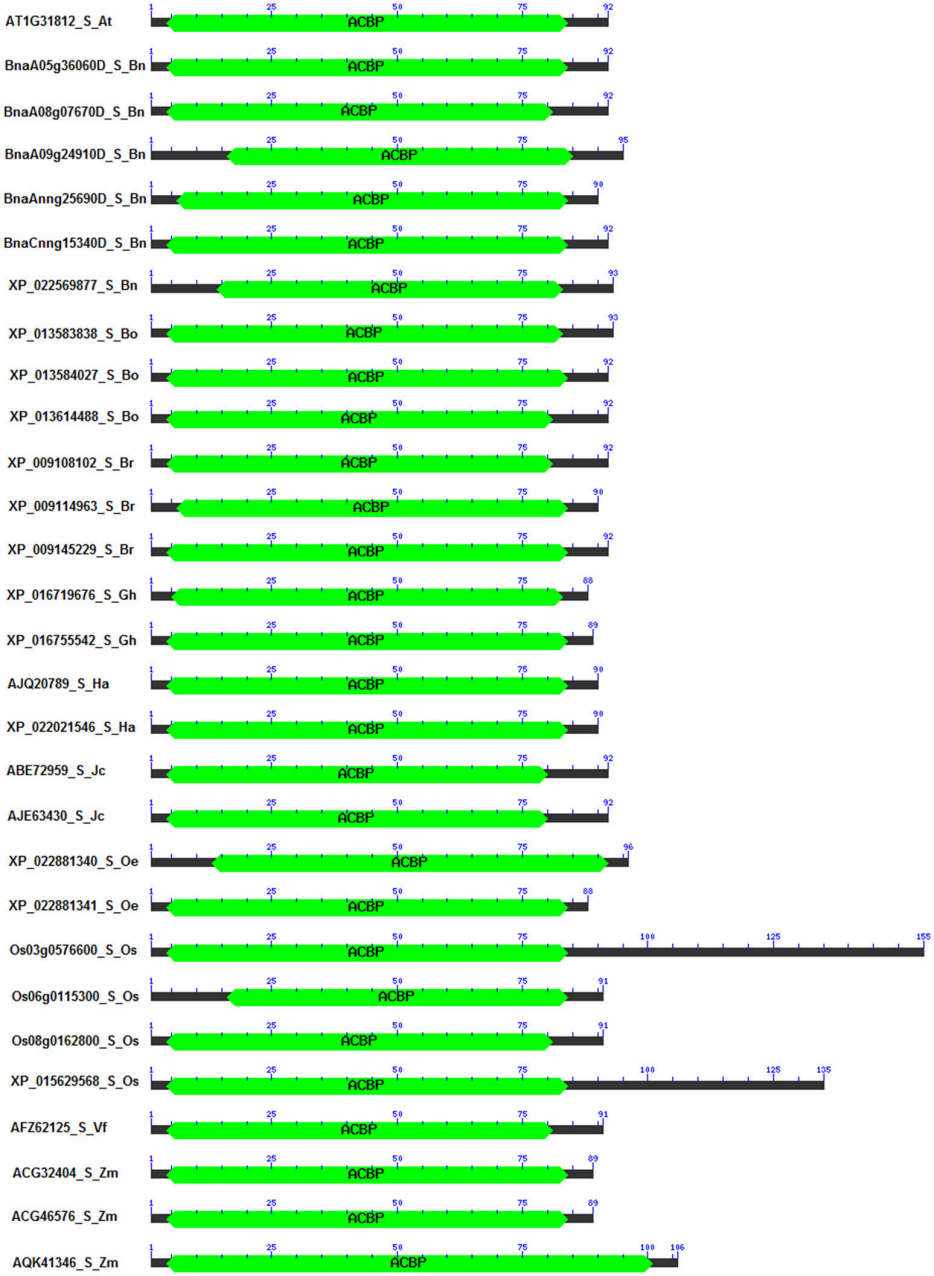
Domain architecture of small ACBP in oil crops. The architecture was generated by using Batch CD-search from NCBI database, using CDSEARCH/oasis_pfam v3 and E-value cut-off of 0.10. ACBD are labeled in green. (Ankyrin repeats, large, and kelch motifs ACBP are in Supplementary Figure 1).

Domains of proteins are important pieces which not only characterize the structure of proteins, but also explain their functions (Itoh et al., 2007). It has been reported that these domains varied within structure and function, and also changed in genome according to the organism (Scheeff and Bourne, 2005; Yang and Bourne, 2009). In our analysis, 186 sequences of ACBP were analyzed, focusing on their domain architecture. Analysis was made using CD search tool of NCBI Database, with CDSEARCH/oasis_pfam v3 as source and e-cut off value of 0.10. Obviously, they all had an acyl-coA binding domain that were located in almost the entire protein in small ACBP, near the C-terminal side of proteins in large ACBP, and near the N-terminal side of proteins in ankyrin repeats and kelch motifs ACBP which both had additional domains, ankyrin and kelch domains, respectively near the C-terminal side of proteins (Figure 4, Supplementary Figure 2). Ankyrin repeats and large ACBP were reported to have N-terminal transmembrane domain that targeted proteins into the membrane (Chye, 1998; Li and Chye, 2003), and which could not be found in our analysis due to the choice of tool used. In previous phylogeny analysis, their positions on the tree were nearby, as their divergences were consecutive; because of that, they were suggested to have related functions (Meng et al., 2011).

A special attention was given to the difference of proteins in the same class of ACBP, among the oil crops studied. Again, similarity or difference in their structure might explain or predict their function. Position and size of domains were observed to be different (Supplementary Table 3). First, in the small ACBP of which protein length varied from 88 Aa (*G. hirsutum* XP_016719676 and *O. europaea* XP_022881341) to 155 Aa (*O. sativa* Os03g0576600), the length of the acyl-coA binding domain varied from 69 Aa (*O. sativa* Os06g0115300, from Aa 16 to Aa 84) to 98 Aa (*Z. mays* AQK41346, Aa 4 to Aa 101), as well as their location on the protein (between Aa 4 and Aa 101). Domain architecture of small ACBP were similar in all studied species. Second, ankyrin repeats ACBP size varied from 267 Aa (*Z. mays* AQK47322) to 367 Aa (*G. hirsutum* XP_016670800, XP_016717349) and acyl-coA binding domain were located between Aa 87 and Aa 204, and their size varied from 27 Aa (*Z. mays* ADT92195) to 86 Aa (*A. thaliana* AT4G27780, *B. napus* BnaC01g20440D, XP_013671647, XP_013700119, XP_013738044, *B. oleracea* XP_013601590, *B. rapa* XP_009143973, *H. annuus* XP_021977528). Besides, these proteins had additional domain which were the ankyrins located near the C-terminal side of proteins, one ankyrin domain were found in each of all proteins, they were located between Aa 154 and Aa 343, their size varied from 54 Aa (*H. annuus* XP_021977528) to 93 Aa (*Z. mays* AQK47322, ADT92195, ACG32907, XP_008667962, ACG24390, *O. sativa* EAY96087, Os04g0681900). Third, the large ACBP had length that varied from 109 Aa (*B. oleracea* XP_013620900) to 525 Aa (*O. sativa* Os03g14000), the size of acyl-coA binding domain varied from 34 Aa (*B. oleracea* XP_013620900) to 87 Aa (*J. curcas* XP_012079368, *O. sativa* Os03g14000), and their locations were more or less near the C-terminal side of protein, which extended between Aa 35 and Aa 502, except for *B. oleracea* XP_013620900 of which acyl-coA binding domain was found on Aa 1, despite its short length and this position of acyl-coA binding domain, *B. oleracea* XP_013620900 was clustered in large ACBP group in the phylogenetic tree. Finally, the domain architecture of kelch motifs ACBP exposed one acyl-coA binding domain that contained 47 Aa (*Z. mays* AQL04248, AQL04250, AQL04251) to 89 Aa (*A. thaliana* AT5G27630) and which were located between Aa 12 and Aa 189. Additionally, one to five kelch were added to the architecture. For example, *B. napus* XP_013732400 had only one kelch motif on its structure which extended from Aa 186 to Aa 227, and *G. max* XP_014627431 had five kelch motifs in Aa 183–224, Aa 299–349, Aa 351–398, and Aa 389–426, respectively. Also, some supplementary domains were found in this class of ACBP that might affect these ACBP functions (Supplementary Figure 2,Supplementary Table 3). In overall, the domain architecture of all studied ACBP presented almost the same profile from each class, but the size of proteins and location of acyl-coA binding domain and additional domains (ankyrin repeats, kelch motifs) made their difference. Besides, ACBP of *A. thaliana* and *B. napus* (*Brassicaceae* tribe) presented almost the same architecture, which was not surprising because they were closer than the other species. More attention should be focused on amino acid sequences of these acyl-coA binding domains to study more about similarity and difference of these ACBP structures, which would be developed more in the next paragraph.

### Acyl-coA binding domain

**Figure 5:**
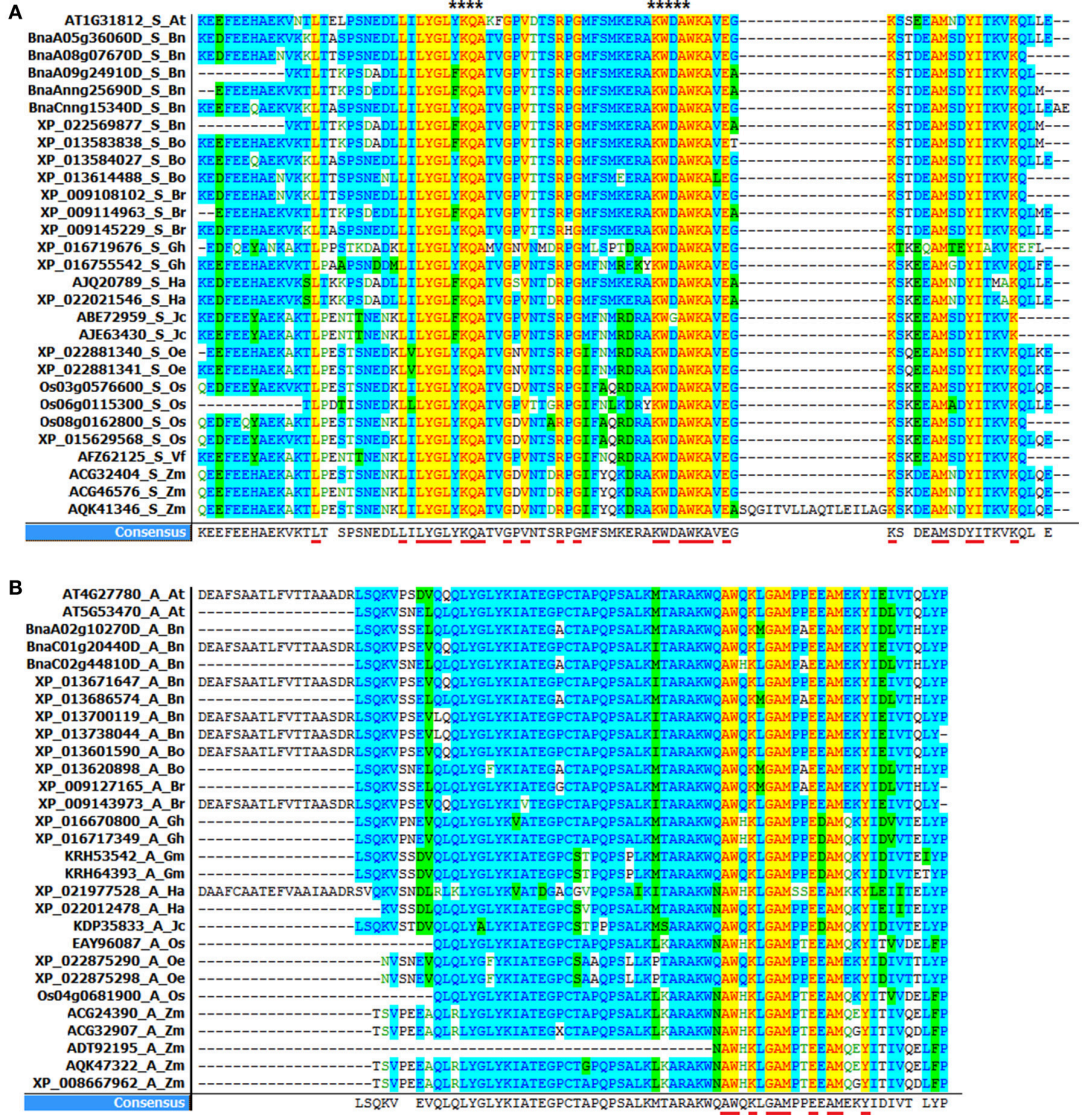
Alignment of acyl-coA binding domain in oil crops. **(A)** ACBD in small ACBP (YKQA and KWDAW motifs are marked with stars at the top of the figure). **(B)** ACBD in ankyrin repeats ACBP. (ACBD alignment in large and kelch motifs ACBP are in Supplementary Figure 2). The alignments were performed using Vector NTI software. Underlined capital letter are highly conserved amino acids in ACBD, and leftover are consensual amino acids found in ACBD.

In the past, it was suggested that differences in amino acid sequences among different classes of ACBP might explain the difference in binding affinities (Emanuelsson et al., 2000; Xie et al., 2015). In this report, we emphasized dissimilarities in acyl-coA binding domain, by alignment of amino acid sequences within the same class of ACBP in different species (Figure 5, Supplementary Figure 3, Supplementary Table 4). Vector NTI software (Lu and Moriyama, 2004) was used to perform the alignment and to calculate the percentage identity of amino acid sequences. Highly conserved amino acids were highlighted, referring to the model plant *A. thaliana*.

Consequently, highly conserved residues in all small ACBP corresponded to 26 residues: Leu-17, Leu-27, Leu-29, Tyr-30, Gly-31, Leu-32, Lys-34, Gln-35, Ala-36, Gly-39, Val-41, Arg-45, Gly-47, Lys-56, Trp-57, Ala-59, Trp-60, Lys-61, Ala-62,Glu-64, Lys-66, Ala-71, Met-72, Tyr-75, Ile-76, Lys-80 in *A. thaliana* AT1G31812, 78 residues were though consensual residues. YKQA and KWDAW motifs which were previously suggested to be essential in binding acyl-CoA esters and conserved in all species (Kragelund et al., 1993; Xiao and Chye, 2011) were present in consensus residues. Then, 65 consensual residues were found in ankyrin repeats ACBP, and 10 among them were highly conserved in all acyl-coA binding domain: Ala-154, Trp-155, Lys-157, Gly-159, Ala-160, Met-161, Glu-164, Ala-166, Met-167, Tyr-170 in *A. thaliana* AT5G53470. In large ACBP, 74 consensual residues were found in large ACBP, but no highly conserved residues were found. Finally, 79 consensual residues were found in kelch motifs ACBP, 18 residues were highly conserved in all species: Gln-54, Gly-58, Pro-59, Pro-65, Trp-68, Glu-72, Trp-76, Ser-78, Trp-79, Leu-82, Met-85, Ala-90, Phe-94, Val-95, Lys-96, Leu-98, Glu-99, Glu-100 in *A. thaliana* AT5G27630. Therefore, in our analysis, acyl-coA binding domain in small ACBP had the highest conserved amino acid in all studied plants.

### Secondary and tertiary structures of ACBP in oil crops

Analysis of protein's secondary structure allows to know the local structural conformation, it is determined by the hydrogen bonds which exist within strands, and which contribute to protein stability and amino acids fold in repeating form. If these hydrogen bonds exist inside a single strand, the protein exhibits an alpha-helix shape, but if two strands are involved, beta-sheet shape is displayed. In our report, secondary structures of ACBP were analyzed using GOR version IV (Garnier et al., 1996; Combet et al., 2000, https://npsa-prabi.ibcp.fr/cgi-bin/npsa_automat.pl?page=npsa_gor4.html). A representative of each accession of ACBP in every class was taken for the analysis. Alpha-helix spaced by extended strands and random coils were obvious in ACBP secondary structure, but extent and proportion varied within species and class of ACBP (Supplementary Figure 4). Small ACBP were mainly composed of alpha-helix shape and random coils, which represented 41.9% of protein (*O. sativa* Os03g0576600) to 64.15% (*Z. mays* AQK41346), and 32.08% (*Z. mays* AQK41346) to 48.35% (*V. fordii* AFZ62125), respectively. In ankyrin repeats ACBP, random coils were rather dominant compared to alpha-helix: 45.20 and 42.09%, respectively in *G. max* KRH53542, to 50.55 and 38.25%, respectively in *J. curcas* KDP35833. Exceptions were obvious in *Z. mays* ACG24390 and *H. annuus* XP_022012478, of which 43.33% of random coils and 47.88% alpha-helix, and 42.69% of random coils and 53.58% of alpha-helix, respectively, were found. Shape of large ACBP displayed similar pattern as in small ACBP, which were mainly composed of alpha-helix and random coils: 47.49% (*Z. mays* ONL95885) to 55.22% (*A. thaliana* AT4G24230) of alpha-helix, and 37.09% (*A. thaliana* AT4G24230) to 44.00% (*V. fordii* AFZ62129) of random coils. Exceptions were also found of which random coils were more dominant, as in *H. annuus* XP_022011286 (16.44% of alpha-helix, 55.43% of random coils) and *G. max* XP_003550311 (39.46% of alpha-helix, 51.47% of random coils), and in *J. curcas* XP_012068250, large ACBP were composed equally of 43.41% of alpha-helix and random coils. Extended strands represented <17% of proteins in small, large, and ankyrin repeats ACBP. However, in kelch motifs ACBP, extended strands were between 16.86% (*Z. mays* AQK61749) and 23.08% (*G. max* XP_006606527). In fact, random coils composed the main structure of kelch motifs ACBP, which were of 45.92% (*H. annuus* XP_021993755) to 50.19% (*Z. mays* AQK61749). Alpha-helix constituted 29.66% (*B. oleracea* XP_013616076) to 35.06% (*O. sativa* XP_015629756).

**Figure 6:**
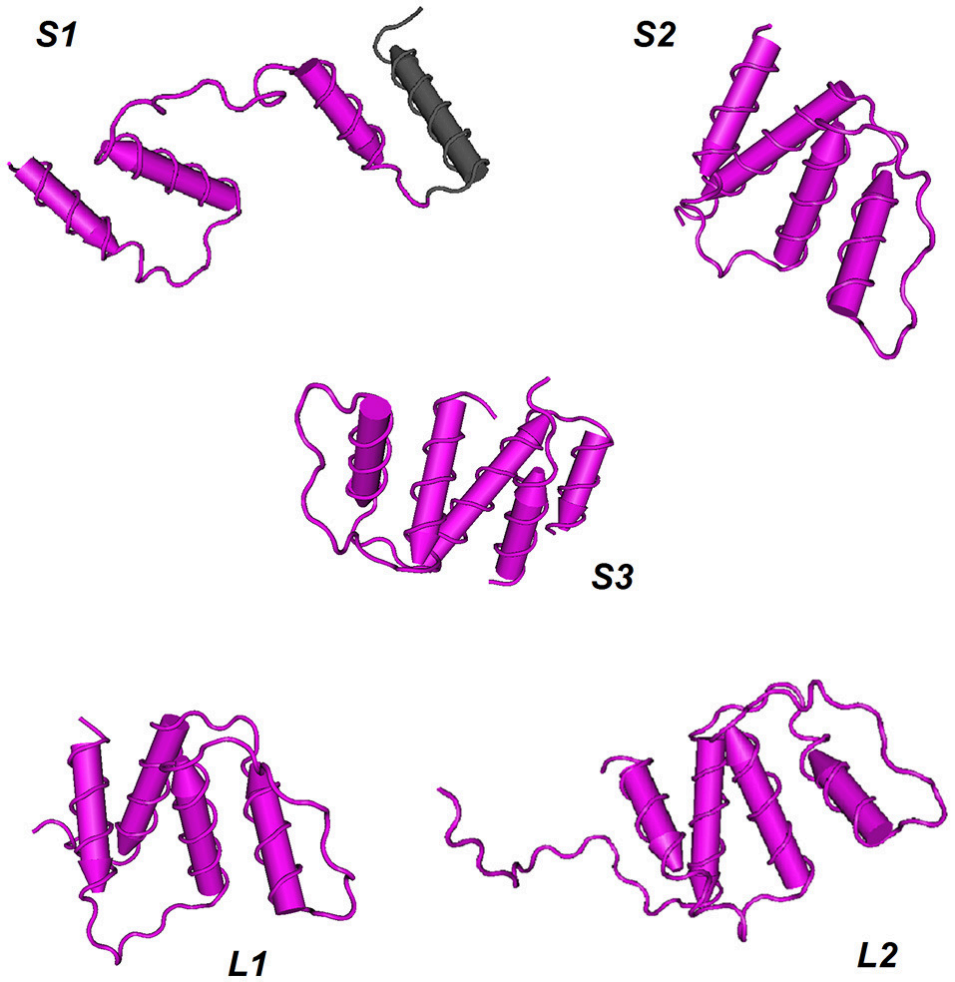
Hypothetical 3D domain structure of ACBP in oil crops. The models were obtained from Phyre2. A part but not full structure of ACBP could be modeled. Conserved domains were highlighted using VAST. ACBD are in pink. S1, S2, and S3 indicates three different model of small ACBP: *A. thaliana, B. napus, B. oleracea*, and *G. hirsutum* could have S1 model, *B. rapa, H. annuus, J. curcas, O. europeae, V. fordii*, and *Z. mays* could have S2 model, and *O. sativa* might have S3 model. Similarly, L1 and L2 indicate two different model of large ACBP, with L1 model for *A. thaliana, B. napus, B. oleracea, B. rapa, H. annuus, V. fordii*, and *Z. mays*, and L2 model for *O. sativa, G. hirsutum, O. europeae, J. curcas*, and *G. max*. Models were classified according to the number of domains and number of helixes or strands found in oil crops ACBP. 3D domain structure of ankyrin repeats and kelch motifs ACBP are in Supplementary Figure 5.

The three-dimensional shapes or tertiary structures of ACBP were also elucidated in oil crops. Tertiary structure is the general folding of helical protein to 3D shape, and it is determined by attractions that offer maximum stability, and lowest energy state. Interaction bound between side-chain group of amino acids contributes to this stability of protein. 3D shapes of ACBP were predicted using Phyre2 (Kelley et al., 2015, http://www.sbg.bio.ic.ac.uk/phyre2/html/page.cgi?id=index_advanced): a batch processing analysis was first performed (multiple protein sequence of ACBP were submitted together at Phyre2 in one analysis), ACBP shape were then predicted based on homology with pre-existing protein on Phyre2 that serves as model. A portion of ACBP protein could be model, and not the total. Then, to highlight the conserved domains of each protein, results obtained from Phyre 2 were submitted for analysis to VAST tool of NCBI (Gibrat et al., 1996, https://www.ncbi.nlm.nih.gov/Structure/VAST/vastsearch.html), and visualized with Cn3D macromolecular structure viewer (Wang et al., 2000; Porter et al., 2007). The observed domains structures of ACBP were then confirmed with SMART (Letunic et al., 2014, http://smart.embl-heidelberg.de/), by using of protein sequences provided with PDB file which were generated from Phyre2. Thus, model of 3D structure of ACBP are represented on Figure 6 and Supplementary Figure 5. The hypothetical 3D structure of ACBP could be subdivided into groups, according to their shape (alpha-helix or beta strands, and their respective amount), and the number of domain detected. In small ACBP, three models of protein could be obtained, both of them displayed dominance of alpha-helix shape. The first model (S1, Figure 5) was for *A. thaliana, B. napus, B. oleracea*, and *G. hirsutum: four* alpha-helixes were found, three of them were predicted to be the acyl-coA binding domain by SMART analysis. The second model (S2, Figure 5) was for *B. rapa, H. annuus, J. curcas, O. europeae, V. fordii*, and *Z. mays*. The four alpha-helixes of this second group were predicted to be the acyl-coA binding domain, and compared to the first model, these alpha-helixes seemed to be closer to each other. The last model (S3, Figure 5) was for *O. sativa* which displayed the same profile as the second model but in difference of having five alpha-helixes rather than four in the second model S2. The same dominant and four alpha-helixes shape was found in the two models of large ACBP (L1 and L2, Figure 5), which differed in an amino acid chain in the second model which did not fit into the alpha-helix shapes (L2, Figure 5). Protein domain analysis confirmed that these alpha-helixes corresponded to the acyl-coA binding domain of ACBP. The 3D structure of ankyrin repeats and kelch motifs ACBP are in Supplementary Figure 5. These two classes of ACBP have additional domain in their protein, apart from the acyl-coA binding domain. So VAST provided results which displayed 5 different domains in different colors. Note that same colors do not indicate the same domain structure. The ankyrin repeats ACBP from the 11 crops studied in our analysis could have nine different models (A1–A9, Supplementary Figure 5): A1 (*O. sativa*,) had four alpha-helixes of which the first domain (pink) had three alpha-helixes and was not recognized by SMART, and the fourth alpha-helix with the second domain (blue) was confirmed by SMART as an ankyrin domain. A2 (*Z. mays*) had five alpha-helixes which were all predicted as ankyrin domains by SMART despite their label as two different protein domains (pink and blue) by VAST. A3 (*A. thaliana*) showed five alpha-helixes of three domains in VAST: the first domain (gray) was not recognized by SMART, the second (pink) and third (blue) domains were predicted as ankyrin domains in SMART. A4 (*B. rapa* and *O. europeae*) displayed six alpha-helixes with two domains, however, the first domain (pink) was predicted as containing both an acyl-coA binding domain and an ankyrin domain, and the second domain (blue) was also an ankyrin domain. Note that in model A1–A4, beta-hairpin shapes were noticeable with alpha-helixes shapes. The model A5 (*B. napus*) contained 17 alpha-helixes of which nine were labeled as domain 1 (pink) containing the acyl-coA binding domain, and eight were domain 2 (blue) with ankyrin domains. A6 (*G. max*) showed 19 alpha-helixes with four domains: the first (gray) and second (pink) domains were displayed in one and three alpha-helixes, respectively, and they were not recognized by SMART, but the third domain (blue) was the acyl-coA binding domain, and the last domain (brown) was the ankyrin domains. In the model A7 (*J. curcas*), 17 alpha-helixes with four domains were displayed, three alpha-helix was highlighted by the first domain (pink) which contain transmembrane region, the second domain (blue) with seven alpha-helixes were the acyl-coA binding domain, the third domain (gray) was not recognized and the last domain (brown) was the ankyrin domain. A8 (*G. hirsutum*) had 18 alpha-helixes of three domains: the first domain (pink) of three alpha-helix contained transmembrane domain, the second domain (blue) of seven alpha-helixes had the acyl-coA binding domain and the third domain (brown) of eight alpha-helixes were the ankyrin domains. The model A9 (*B. oleraceae* and *H. annuus*) had 16 alpha-helixes with three domains, the first domain (pink) of four alpha-helixes were not recognized by SMART, the second domain (blue) of seven alpha-helixes were the acyl-coA binding domain and the last domain of six alpha-helixes were the ankyrin domain. Finally in kelch motifs ACBP, four models could be found, which exclusively had beta-sheets, except for the model K4: in fact, the model K1 (*Z. mays*) had three domains of kelch domains, K2 (*G. max*) had five domains that correspond all to kelch domains unless for the last domain (gray) which was not recognized by SMART, K3 (*A. thaliana, B. napus, B. oleraceae, G. hirsutum, H. annuus, J. curcas, O. europeae, O. sativa*, and *V. fordii*) had four domains (pink, blue, green, brown) corresponding to kelch motifs, and K4 (*B. rapa*) had five domains, of which the first domain (pink) had five alpha-helix and some beta-sheets containing the acyl-coA binding domain, and the remaining four domains had all beta-sheets in which three domains (blue, green, and brown) correspond to kelch domains and the last one (gray) was not recognized by SMART.

Taken together the findings on the secondary and tertiary structures of these oil crops' ACBP, it is obvious that alpha-helixes shapes were dominant in small and large ACBP. This finding is consistent with ACBP structure found in *B. taurus* (Andersen and Poulsen, 1992; Kragelund et al., 1993), in *P. falciparum* (van Aalten et al., 2001), in *S. cerevisiae* (Teilum et al., 2005), and in *H. sapiens* (Taskinen et al., 2007), as mentioned above. However, ankyrin repeats and kelch motifs ACBP displayed less dominance of alpha-helixes in overall, probably because of additional domains presents in these proteins. In fact, ankyrin and kelch domains have their individual shape. Ankyrin are composed by 30% of alpha-helixes and beta-hairpin loop in L-shape (Davis and Bennett, 1990; Gorina and Pavletich, 1996; Rubtsov and Lopina, 2000). It is thus understandable that in our findings, both alpha-helixes and beta-hairpin shapes were seen, but in secondary structure, it was clear that alpha-helixes were not dominant, and in 3D structure, dominance of alpha-helixes might be explain by the fact that a part of proteins could be modeled in Phyre2, maybe different model could be obtained if the total proteins could be modeled. As well, kelch motifs proteins display beta-propellers containing tandem of kelch. One propeller is composed of four-stranded beta-sheets with kelch motifs (Adams et al., 2000). This is consistent with our findings in which kelch domains had beta-sheets shape. Therefore, it is clear that natural form of acyl-coA binding domain is in alpha-helix shape, form of ankyrin repeats is a mix of alpha-helix and beta-hairpin, and form of kelch motifs is in beta-sheets. Based on our analysis, the fact that ACBP of the same class could not display exactly the same configuration might be due to difference in amino acid sequences. Despite this difference, some ACBP from different species could obviously display the same configuration. Our findings were just prediction; experimental approaches are needed for confirmation.

### Subcellular localization of ACBP in oil crops

Subcellular locations of ACBP were predicted using two different tools: the first tool was TargetP1.1 (http://www.cbs.dtu.dk/services/TargetP/) which predict the location based on presence of N-terminal pre-sequences related to chloroplast, mitochondria, or secretory pathway signal peptide (Emanuelsson et al., 2000), and the second tool was MultiLoc2 (http://abi.inf.uni-tuebingen.de/Services/MultiLoc2) which analyzed proteins in a large scale giving more accurate and robust results (Blum et al., 2009). Predicted subcellular locations of ACBP are presented in Supplementary Table 5. Thus, based on TargetP1.1, small ACBP and kelch motifs ACBP were suggested to be located in other places than in chloroplast, or mitochondria, except for the kelch motif ACBP of *Z. mays* AQK61749 which might be found in chloroplast. However, ankyrin repeats and large ACBP might be related to secretory pathway. Value of reliability class indicated strong prediction all classes of ACBP except for kelch motifs ACBP. Multiloc2 gave more precise results: in all oil crops, small and kelch motifs ACBP were all predicted to be located in cytoplasm; additionally, kelch motifs ACBP of *H. annuus* (XP_021993755) and *Z. mays* (AQK61749) were likely to be located in mitochondria as well. Similarly, ankyrin repeats and large ACBP in all oil crops were predicted to be located in ER.

Furthermore, based on MultiLoc2 analysis, ankyrin repeats ACBP of *A. thaliana* (AT4G27780), *G. max* (KRH53542), *J. curcas* (KDP35833) might be located in outer cell, in golgi apparatus and in plasma membrane, large ACBP of *H. annuus* (XP_022011286) and *V. fordii* (AFZ62129) might be outer cell and in vacuole, and in plasma membrane also for *V. fordii* (AFZ62129), and extracellular for large ACBP of *Z. mays* ONL95885. Kelch motifs ACBP were also found in chloroplast and mitochondria for some species: *B. napus* (AIS76197) and *V. fordii* (AFZ62126) in chloroplast, *Z. mays* (AQK61749) in mitochondria, and *H. annuus* (XP_021993755) in both chloroplast and mitochondria.

Subcellular localizations of ACBP were predicted based on amino acid sequences. In *A thaliana, B. napus, H. annuus, V. fordii*, and *O. sativa, in vitro* analyses of ACBP subcellular localization were already performed as cited above, and compared to our analysis, consistencies were found in small and kelch motifs ACBP, which were located in cytoplasm, and large and ankyrin repeats ACBP in ER and plasma membrane. However, MultiLoc2 neither displayed significant presence of *A. thaliana* large ACBP in outer cell, nor *O. sativa* kelch motifs ACBP in peroxysome. At the end, findings from *in-vitro* analysis are always more trustworthy than from *in silico* analysis. Our findings might encourage to perform *in-vitro* analysis of subcellular localization of oil crops ACBP.

## Structure-function relationship in oil crops ACBP

Several reports gave a comprehensive review on structure-function relationship in proteins (Hegyi and Gerstein, 1999; Orengo et al., 1999; Lee et al., 2007; Redfern et al., 2008; Sadowski and Jones, 2009; Fang et al., 2010; Uversky and Dunker, 2010; Polanco et al., 2016). Also, studies on particular proteins have been reported (Mills et al., 1995; Mulakala and Reilly, 2002; Albenne et al., 2009; Feng et al., 2009; Payne et al., 2011; Scavuzzo-Duggan et al., 2015), and quite a few approaches have been even proposed for function prediction or for structure-function relationship studies (Guerrucci and Bell, 1995; Lavery and Sacquin-Mora, 2007; Xie et al., 2007; Atkinson et al., 2009; Wu et al., 2010; Osadchy and Kolodny, 2011; Micheletti, 2013; Cohn, 2016; Mudgal et al., 2017).

**Figure 7:**
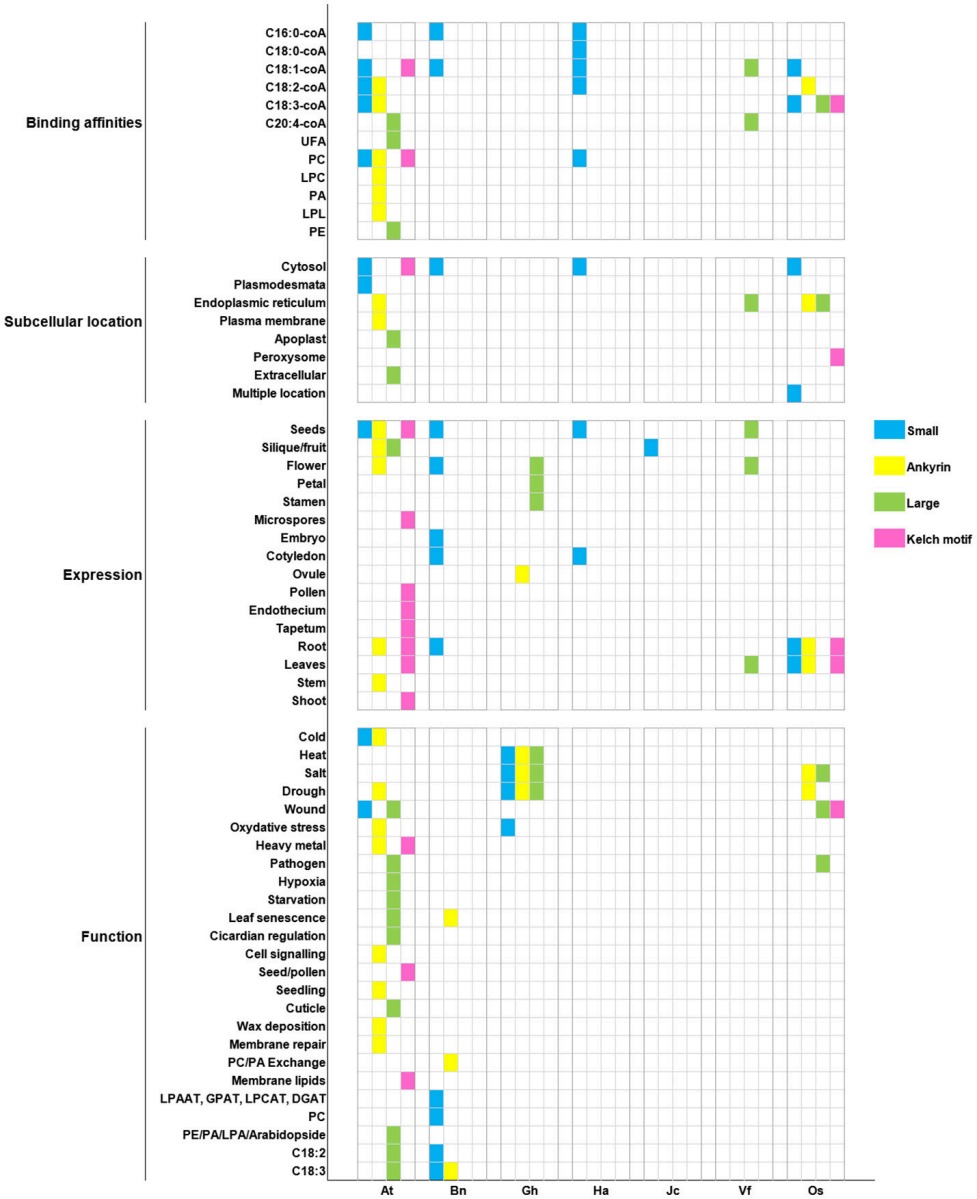
Recapitulation of ACBP characteristics in oil crops. Oil crop species are respectively arranged on X axis with the model plant *A. thaliana* (At): Bn (*B. napus*), Gh (*G. hirsutum*), Ha (*H. annuus*), Jc (*J. curcas*), Vf (*V. fordii*), Os (*O. sativa*). Binding affinities, subcellular location, tissues expression, and function of ACBPs are, respectively arranged on Y axis. Different classes of ACBP are shown in different color, as indicated on the left side of the figure.

Formerly, we summarized findings on function of ACBP in 11 oil crops alongside the model plant *A. thaliana*, and then we analyzed their structure through comparison. It was obvious that ACBP exhibited diversity in both structure and function. We recapitulated the major findings in Figure 7, thus conserved features among ACBP could be clearly seen. In technical term, ortholog proteins exist in different species, they are results of speciation events, and they are presumed to have similar function; in contrast to paralogs that exist within the same species, and are supposed to have different function (Koonin et al., 1996; Tatusov et al., 1997; Fitch, 2000; Koonin, 2005; Gabaldón and Koonin, 2013). The foundation of functional annotation of sequenced genome arose from orthology and function inference (Koonin, 2005; Descorps-Declère et al., 2008).

In our report, cases were found in which orthologous ACBP had different function (e.g., AtACBP6 and GhACBP6, BnACBP6), and paralogous ACBP had similar function (e.g., GhACBP1, GhACBP3, and GhACBP6 in response to heat treatment). Difference was not limited only in function, but also in subcellular localization and expression. No fixed subcellular location was found, but as known in *A. thaliana*, small and kelch motifs ACBPs were found in cytosol and large and ankyrin repeats ACBPs were in the ER, so orthologs BnACBP6, HaACBP6, OsACBP1, OsACBP4, OsACBP5, and VfACBP3 resided in the same location as in *A. thaliana*. The binding affinities were in majority non-specified, except for C16:0-coA and C20:4-coA which could be bound by small ACBP in *A. thaliana, B. napus*, and *H. annuus*, and large ACBP in *A. thaliana* and *V. fordii*, respectively. Then, a conserved expression of small ACBP in cotyledons was observed only in *B. napus* and *H. annuus*, remaining ACBP displayed non-exclusivity for a particular tissue. Lastly, pathogen response was the only function involving large ACBP only in *A. thaliana* and *O. sativa*. Indeed, single class of ACBP displaying unique characteristic existed. They could be qualified as having specified function, as for example, AtACBP3 involvement in hypoxia and starvation stresses, or the implication of AtACBP5 in seed and pollen development. But our conclusions could not be definitive, because they were deduced only from selected function tested in respective study. Lack of research in other species might encourage more researches to be performed.

Thus, based on our analysis, orthologs ACBP rarely conserved similar function in oil crops. It has been demonstrated in some studies that orthologs functional differences were larger than expected and might be at the same level of difference as in paralogs with different sequences (Studer and Robinson-Rechavi, 2000; Nehrt et al., 2011). In fact, functional diversity among orthologous genes might be due to specific functional region, it has been demonstrated in our analysis that ortholog proteins sequence and length were different, which implied that some regions were missing or varied in proteins. It has been suggested that few non-conservative amino acid mutations might cause significant functional differences, single mutation might be insufficient or deleterious, but multiple simultaneous mutations might be enough to change function without harm (Canepari et al., 2000). Additionally, orthologs functional diversity might be due to the imprecise methods for comparing gene functions in different organisms, the differences caused by species specific environments, and the overall ambiguity of the genotype to-phenotype mapping (Gabaldón and Koonin, 2013).

Besides, functional similarity in paralogs ACBP was also observed in our analysis. Paralogous genes emerged after duplication event, many duplicated genes diverged that no sequence similarity was found, they formed gene families (Zhang, 2013), as in ACBP. Zhang (2013) affirmed that paralogous genes that conserved similar function might be due to gene conversion after concerted evolution, in which proteins have very similar sequences and function, and purifying selection against mutations in which function of genes are modified to prevent divergence. Moreover, it has been affirmed that proteins that differed in structure might have converged to similar active sites, catalytic mechanism and biochemical function. Active-site residues and structure are conserved despite remaining sequence divergence that made the structure obviously dissimilar. This is the case of proteins that underwent convergent evolution, in which they have not evolved from a same ancestor, but autonomously and converged on the same active-site because of natural selection for particular function (Petsko and Ringe, 2004). ACBP have though common ancestor, and diverged throughout evolution as explained earlier, they had in common the acyl-coA binding domain, despite the fact that sequence in this region were not perfectly similar, probably similar function could be maintained due to conserved active-site residues.

## Conclusion

In conclusion, this review aimed to comprehend diversity in ACBP of 11 selected oil crops. ACBP were subdivided into four classes according to their domain structure. Separate inquiries on these ACBP enriched our knowledge on their function, subcellular location, tissues expression, and structure. In overall, ACBP in oil crops have important function in lipid metabolism, membrane biosynthesis and repair, cell signaling, plant development, stress management, and disease resistance. Though, structure comparison displayed low and high similarity among oil crops ACBPs, however, orthologs ACBP diverged in function, and paralogs had analogous functions, rare cases were found in which they had same function or same tissue expression, but in majority, these ACBPs of dissimilar protein domain displayed similar tissue expression and might act for the same role. It is uncertain whether specified structure could be assigned to a fixed function, which might weaken the concept of function prediction based on structure. Ultimately, it is confirmed that proteins from closely related species could not necessarily display the same function. Because findings from *in-vitro/in-vivo* analyses are far better than *in-silico* analyses, it is greatly needed to perform experimental studies related to ACBP structure, subcellular location and function. Moreover, ACBP from other oil crops as palm, peanut, and coconut should be isolated and characterized, and function of ACBP in broccoli, turnip, and maize should be studied. Additional studies in these oil crops ACBP are greatly encouraged to encounter new functions, in the purpose of altering crops to benefit valuable traits.

## Author contributions

NR wrote the manuscript. ML, BW, and LY supervised the work and revised the manuscript.

### Conflict of interest statement

The authors declare that the research was conducted in the absence of any commercial or financial relationships that could be construed as a potential conflict of interest.

## Supplementary material

Supplementary Table 1 List of oil crops species and their ACBP.

Supplementary Table 2 Amino acid sequence identity in oil crops ACBP.

Supplementary Table 3 Domain location in oil crops ACBP.

Supplementary Table 4 Amino acid sequence identity in oil crops ACBD.

Supplementary Table 5 Predicted subcellular localization of ACBP in oil crops.

Supplementary Figure 1 Heat map depicting amino acid sequence identity of oil crops ACBP. **(A)** large ACBP, **(B)** kelch motifs ACBP. The map was generated by Heatmapper (Babicki et al., 2016), with Pearson's correlation matrix calculation. Color intensity change with correlation value as indicated.

Supplementary Figure 2 Domain architecture of ankyrin repeats **(A)**, large **(B)**, and kelch motif **(C)** ACBP in oil crops. The architecture was generated by using Batch CD-search from NCBI database, using CDSEARCH/oasis_pfam v3 and *E*-value cut-off of 0.10. ACBD are labeled in green, ankyrin repeats are in blue, and kelch domains are in purple.

Supplementary Figure 3 Alignment of acyl-coA binding domain in large **(A)** and kelch motifs **(B)** ACBP of oil crops. The alignments were performed using Vector NTI software. Underlined capital letter are highly conserved amino acids in ACBD, and leftover are consensual amino acids found in ACBD.

Supplementary Figure 4 Secondary structure of ACBP in oil crops. The prediction was made using GOR version IV (Garnier et al., 1996; Combet et al., 2000).

Supplementary Figure 5 Hypothetical 3D domain structure of ACBP in oil crops. The model were obtained from Phyre2. A part but not full structure of ACBP could be modeled. Conserved domain were highlighted using VAST. ACBD are in pink, ankyrin repeats are in blue, kelch motifs are in blue, brown, and green, unknown domain is highlighted in gray. A1 to A9 indicate different model of ankyrin repeats ACBP: A1 (*O. sativa*), A2 (*Z. mays*), A3 (*A. thaliana*), A4 (*B. rapa* and *O. europeae*), A5 (*B. napus*), A6 (*G. max*), A7 (*J. curcas*), A8 (*G. hirsutum*), A9 (*B. oleraceae* and *H. annuus*). K1–K4 indicate different model of kelch motif ACBP: K1 (*Z. mays*), K2 (*G. max*), K3 (*A. thaliana, B. napus, B. oleraceae, G. hirsutum, H. annuus, J. curcas, O. europeae, O. sativa*, and *V. fordii*), K4(*B. rapa*). Models were classified according to the number of domains and number of helixes or strands found in oil crops ACBP.
