# Supplementary figures and images for: Functional and Structural Diversity of Acyl-coA Binding Proteins in Oil Crops
Source: Front Genet. 2018 May 22;9:182. doi: 10.3389/fgene.2018.00182 (PMC5972291; doi:10.3389/fgene.2018.00182)

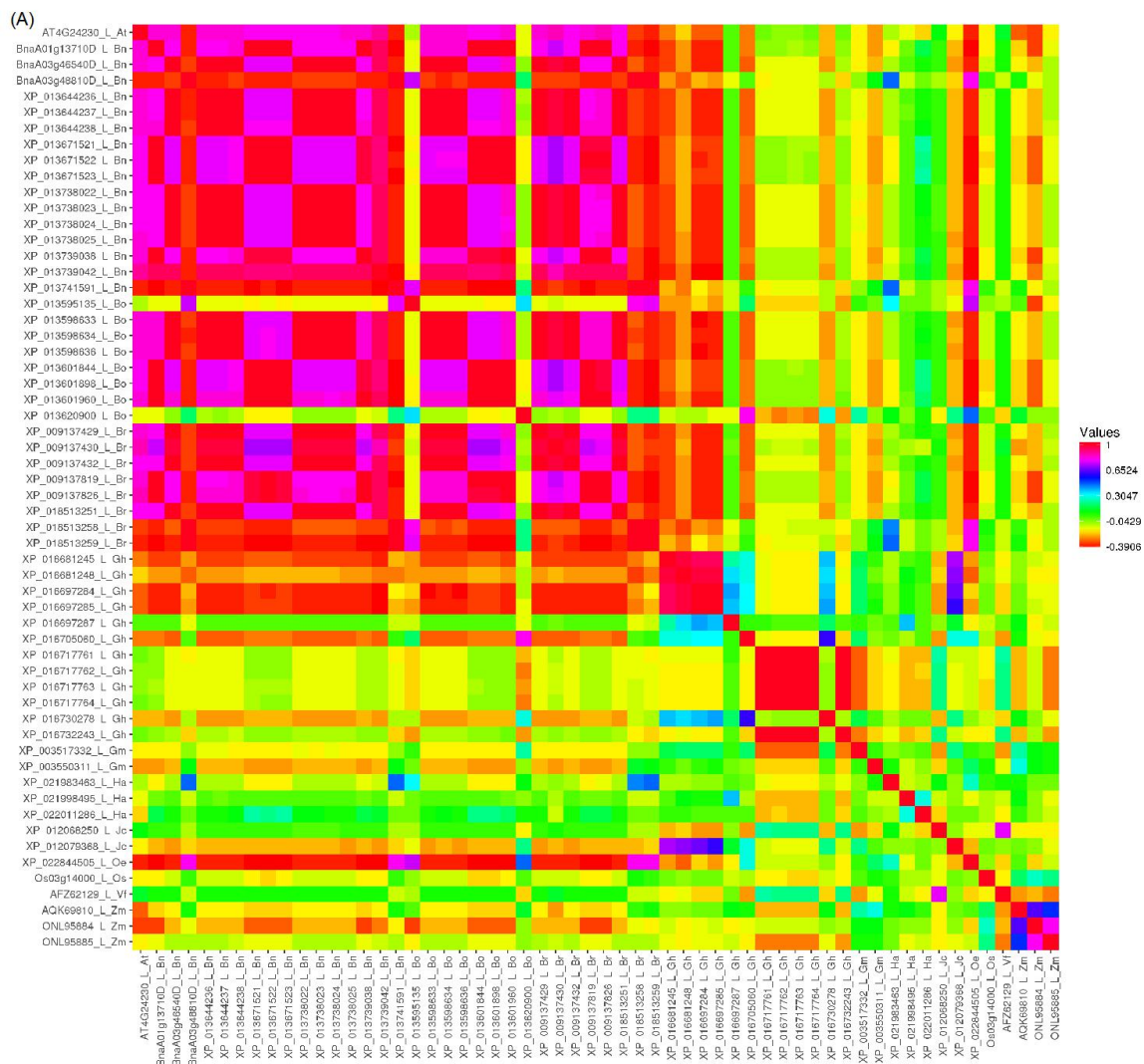

Supplement: Supplementary Figure 1 — Heat map depicting amino acid sequence identity of oil crops ACBP. (A) large ACBP, (B) kelch motifs ACBP. The map was generated by Heatmapper (Babicki et al., 2016), with Pearson's correlation matrix calculation. Color intensity change with correlation value as indicated. [file Image_1.PDF]
