# Supplementary material for: Functional and Structural Diversity of Acyl-coA Binding Proteins in Oil Crops
Source: Front Genet. 2018 May 22;9:182. doi: 10.3389/fgene.2018.00182 (PMC5972291; doi:10.3389/fgene.2018.00182)
Supplement: Supplementary Figure 2 — Domain architecture of ankyrin repeats (A), large (B), and kelch motif (C) ACBP in oil crops. The architecture was generated by using Batch CD-search from NCBI database, using CDSEARCH/oasis_pfam v3 and E-value cut-off of 0.10. ACBD are labeled in green, ankyrin repeats are in blue, and kelch domains are in purple. [file Image_2.PDF]

(A)

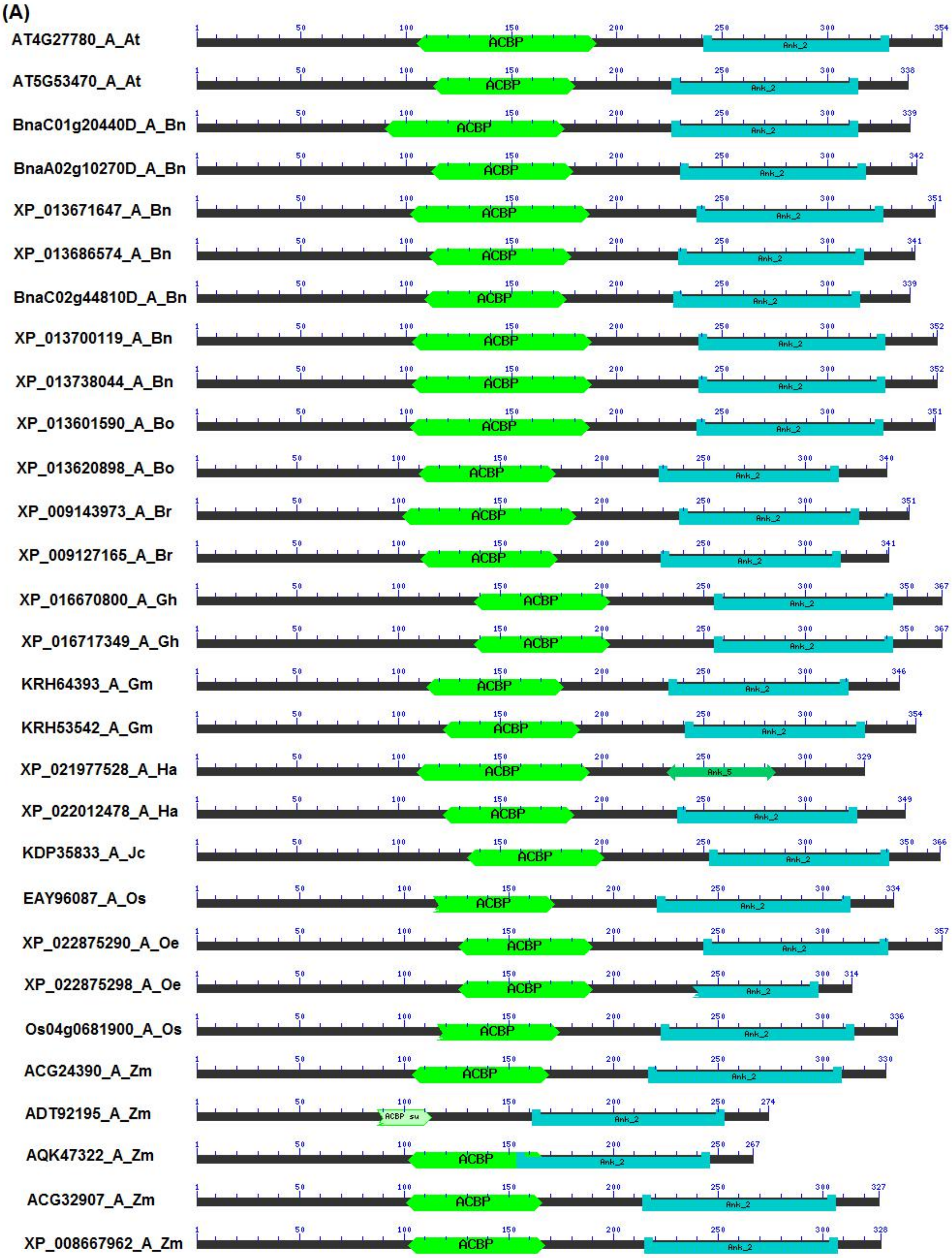
AT4G27780_A_At
AT5G53470_A_At
BnaC01g20440D_A_Bn
BnaA02g10270D_A_Bn
XP_013671647_A_Bn
XP_013686574_A_Bn
BnaC02g44810D_A_Bn
XP_013700119_A_Bn
XP_013738044_A_Bn
XP_013601590_A_Bo
XP_013620898_A_Bo
XP_009143973_A_Br
XP_009127165_A_Br
XP_016670800_A_Gh
XP_016717349_A_Gh
KRH64393_A_Gm
KRH53542_A_Gm
XP_021977528_A_Ha
XP_022012478_A_Ha
KDP35833_A_Jc
EAY96087_A_Os
XP_022875290_A_Oe
XP_022875298_A_Oe
Os04g0681900_A_Os
ACG24390_A_Zm
ADT92195_A_Zm
AQK47322_A_Zm
ACG32907_A_Zm
XP_008667962_A_Zm
ACBP
Ank_2
Ank_5
ACBP su

(B)

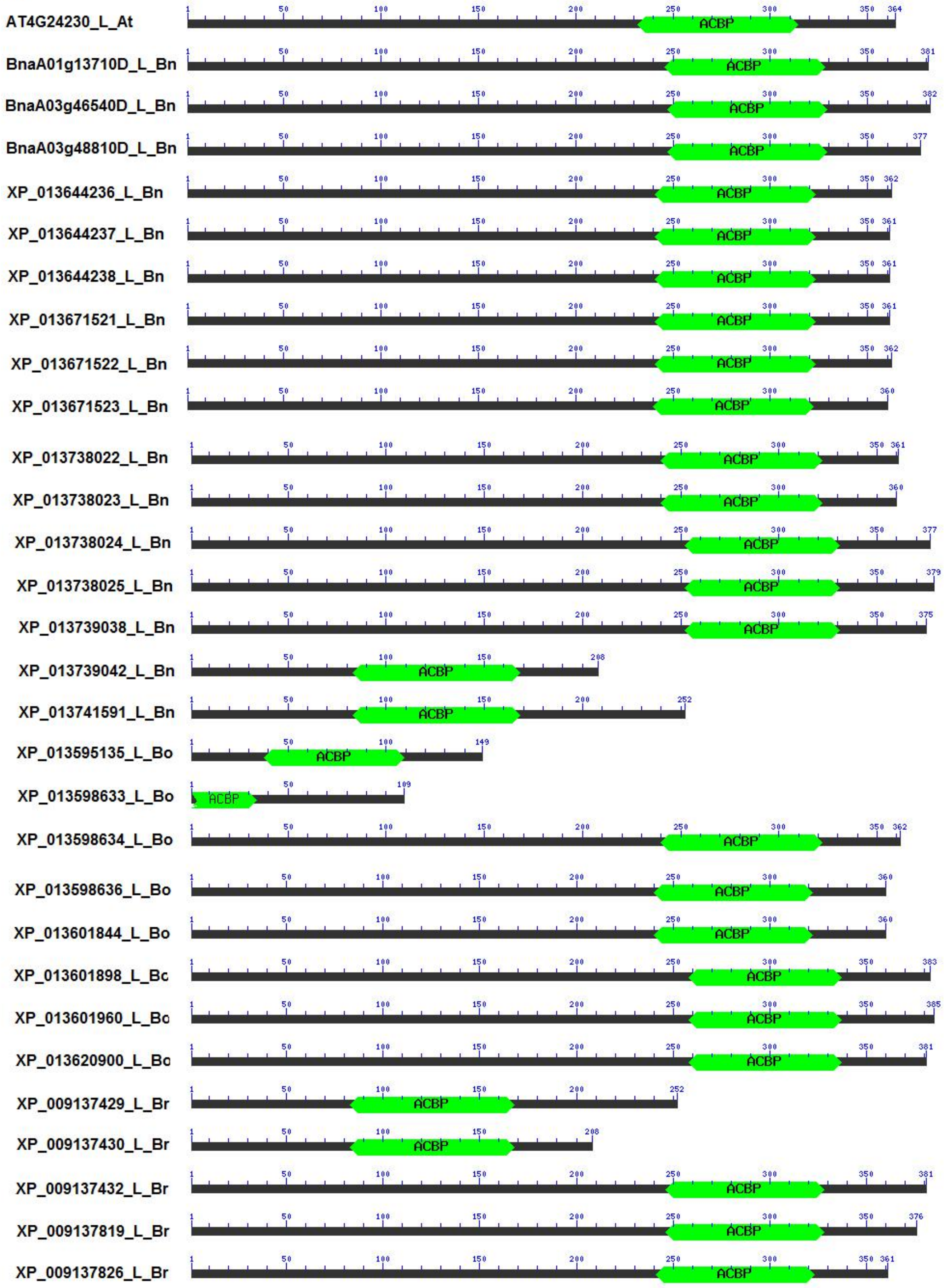
AT4G24230_L_At
BnaA01g13710D_L_Bn
BnaA03g46540D_L_Bn
BnaA03g48810D_L_Bn
XP_013644236_L_Bn
XP_013644237_L_Bn
XP_013644238_L_Bn
XP_013671521_L_Bn
XP_013671522_L_Bn
XP_013671523_L_Bn
XP_013738022_L_Bn
XP_013738023_L_Bn
XP_013738024_L_Bn
XP_013738025_L_Bn
XP_013739038_L_Bn
XP_013739042_L_Bn
XP_013741591_L_Bn
XP_013595135_L_Bo
XP_013598633_L_Bo
XP_013598634_L_Bo
XP_013598636_L_Bo
XP_013601844_L_Bo
XP_013601898_L_Bo
XP_013601960_L_Bo
XP_013620900_L_Bo
XP_009137429_L_Br
XP_009137430_L_Br
XP_009137432_L_Br
XP_009137819_L_Br
XP_009137826_L_Br
ACBP

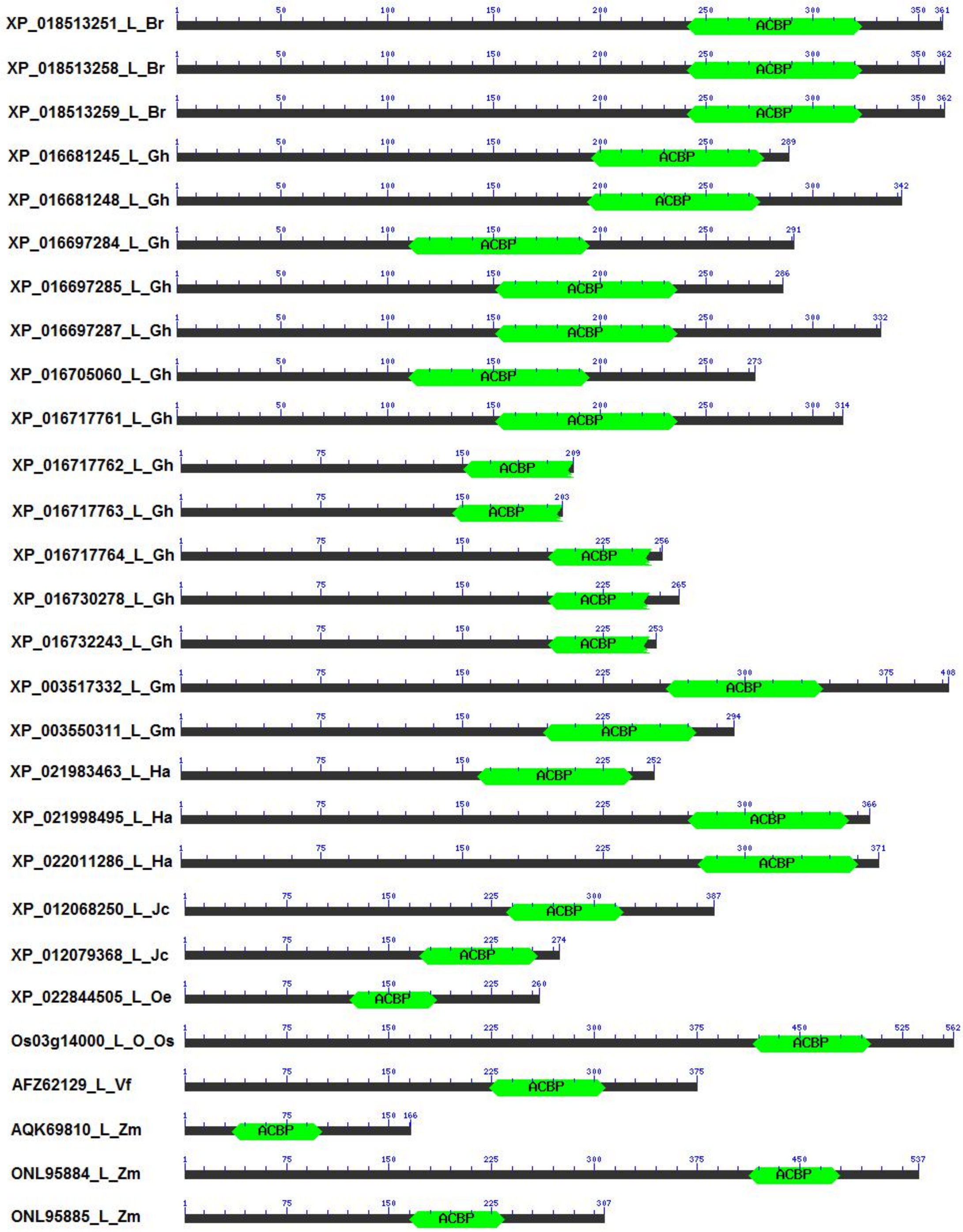

XP_018513251_L_Br
XP_018513258_L_Br
XP_018513259_L_Br
XP_016681245_L_Gh
XP_016681248_L_Gh
XP_016697284_L_Gh
XP_016697285_L_Gh
XP_016697287_L_Gh
XP_016705060_L_Gh
XP_016717761_L_Gh
XP_016717762_L_Gh
XP_016717763_L_Gh
XP_016717764_L_Gh
XP_016730278_L_Gh
XP_016732243_L_Gh
XP_003517332_L_Gm
XP_003550311_L_Gm
XP_021983463_L_Ha
XP_021998495_L_Ha
XP_022011286_L_Ha
XP_012068250_L_Jc
XP_012079368_L_Jc
XP_022844505_L_Oe
Os03g14000_L_O_Os
AFZ62129_L_Vf
AQK69810_L_Zm
ONL95884_L_Zm
ONL95885_L_Zm
ACBP

(C)

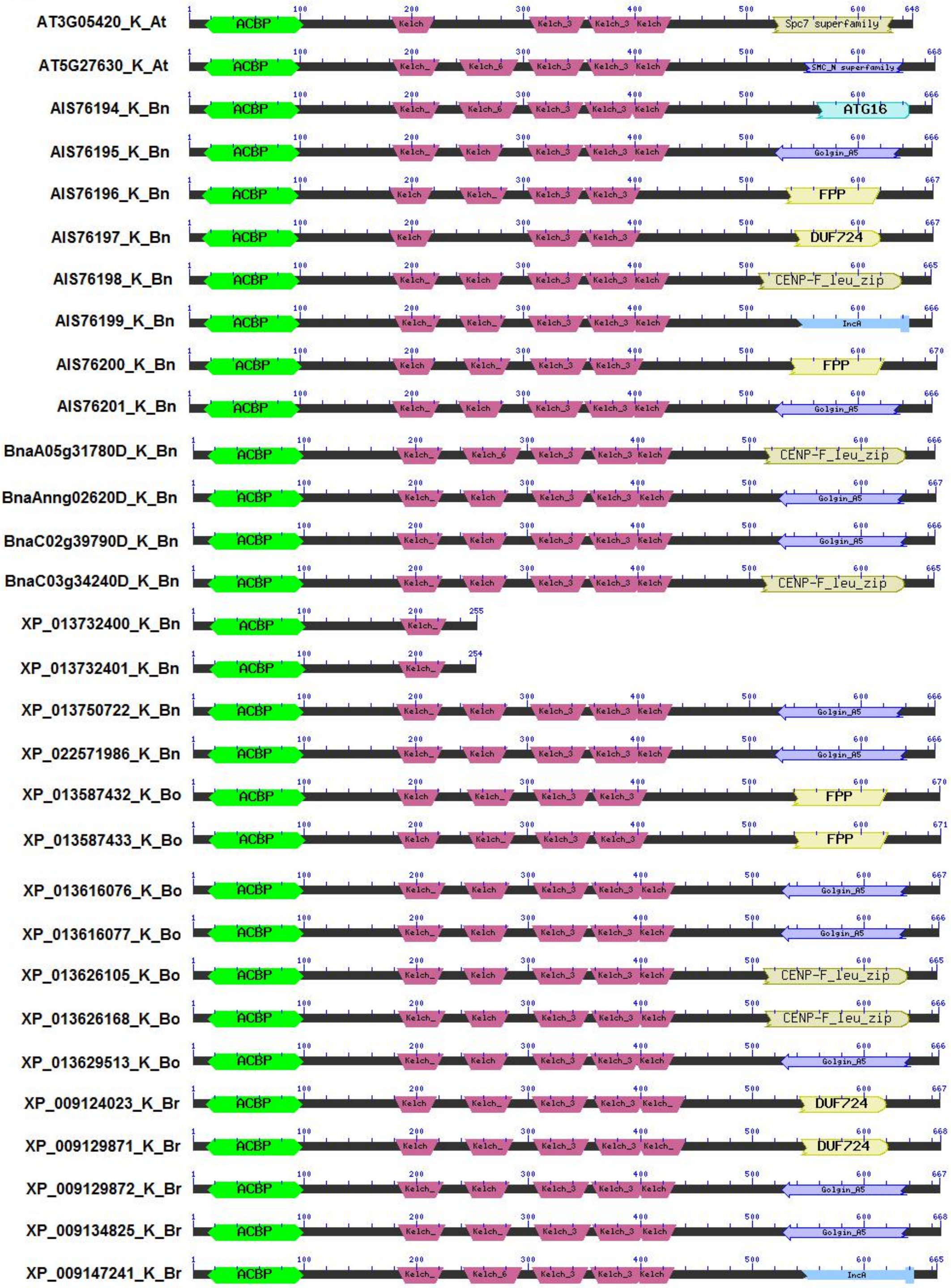
AT3G05420_K_At
AT5G27630_K_At
AIS76194_K_Bn
AIS76195_K_Bn
AIS76196_K_Bn
AIS76197_K_Bn
AIS76198_K_Bn
AIS76199_K_Bn
AIS76200_K_Bn
AIS76201_K_Bn
BnaA05g31780D_K_Bn
BnaAnng02620D_K_Bn
BnaC02g39790D_K_Bn
BnaC03g34240D_K_Bn
XP_013732400_K_Bn
XP_013732401_K_Bn
XP_013750722_K_Bn
XP_022571986_K_Bn
XP_013587432_K_Bo
XP_013587433_K_Bo
XP_013616076_K_Bo
XP_013616077_K_Bo
XP_013626105_K_Bo
XP_013626168_K_Bo
XP_013629513_K_Bo
XP_009124023_K_Br
XP_009129871_K_Br
XP_009129872_K_Br
XP_009134825_K_Br
XP_009147241_K_Br
ACBP
Kelch
Kelch_3
Spc7 superfamily
ATG16
FPP
DUF724
CENP-F_leu_zip
Golgin_A5
IncA

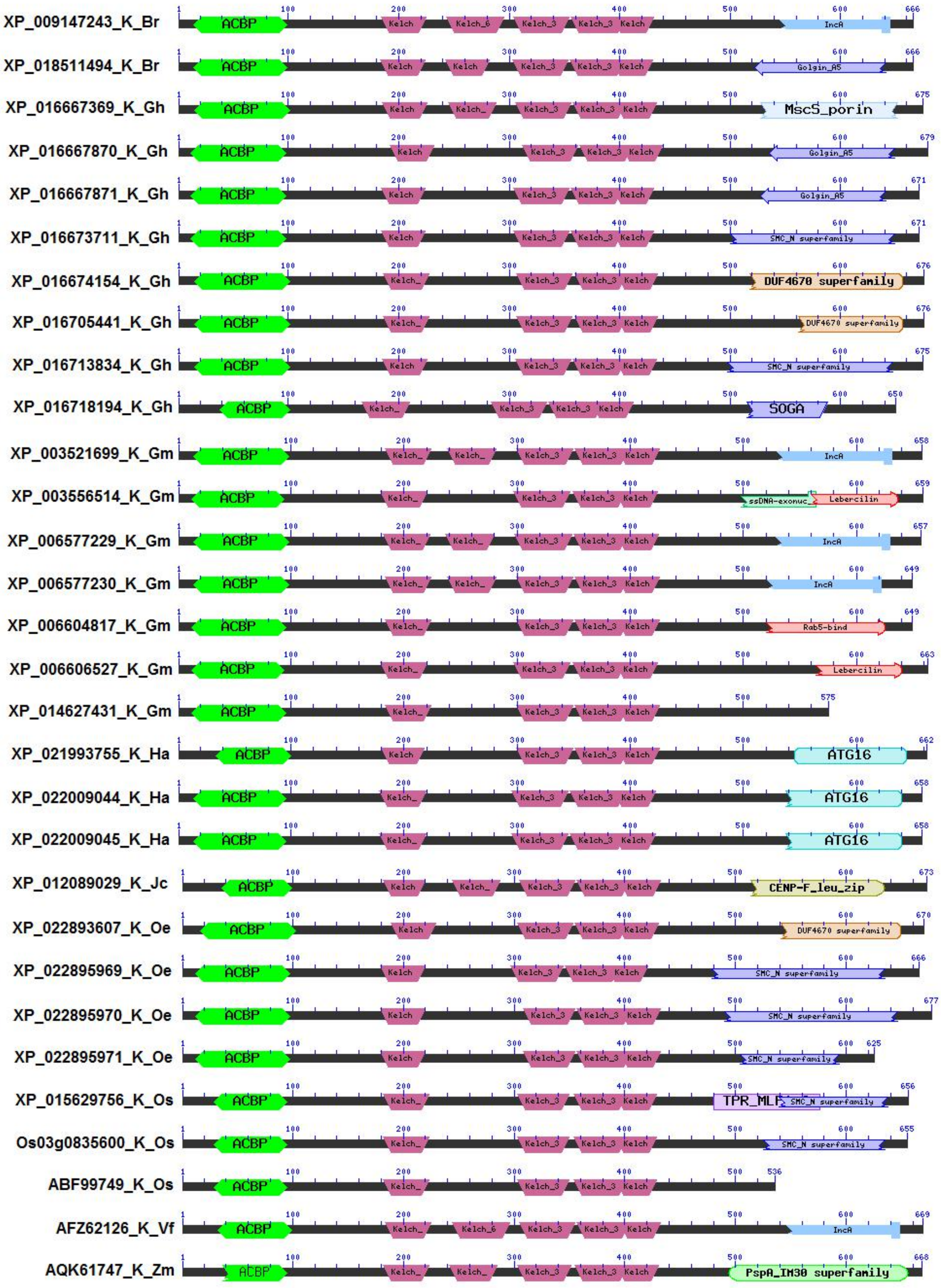

XP_009147243_K_Br
ACBP Kelch Kelch_6 Kelch_3 Kelch_3 Kelch IncA
XP_018511494_K_Br
ACBP Kelch Kelch Kelch_3 Kelch_3 Kelch Golgin_A5
XP_016667369_K_Gh
ACBP Kelch Kelch_ Kelch_3 Kelch_3 Kelch MscS_porin
XP_016667870_K_Gh
ACBP Kelch Kelch_3 Kelch_3 Kelch Golgin_A5
XP_016667871_K_Gh
ACBP Kelch Kelch_3 Kelch_3 Kelch Golgin_A5
XP_016673711_K_Gh
ACBP Kelch Kelch_3 Kelch_3 Kelch SMC_N superfamily
XP_016674154_K_Gh
ACBP Kelch_ Kelch_3 Kelch_3 Kelch DUF4670 superfamily
XP_016705441_K_Gh
ACBP Kelch_ Kelch_3 Kelch_3 Kelch DUF4670 superfamily
XP_016713834_K_Gh
ACBP Kelch Kelch_3 Kelch_3 Kelch SMC_N superfamily
XP_016718194_K_Gh
ACBP Kelch_ Kelch_3 Kelch_3 Kelch SOGA
XP_003521699_K_Gm
ACBP Kelch_ Kelch_ Kelch_3 Kelch_3 Kelch IncA
XP_003556514_K_Gm
ACBP Kelch_ Kelch_3 Kelch_3 Kelch ssDNA-exonuc_ Lebercilin
XP_006577229_K_Gm
ACBP Kelch_ Kelch_ Kelch_3 Kelch_3 Kelch IncA
XP_006577230_K_Gm
ACBP Kelch_ Kelch_ Kelch_3 Kelch_3 Kelch IncA
XP_006604817_K_Gm
ACBP Kelch_ Kelch_3 Kelch_3 Kelch Rab5-bind
XP_006606527_K_Gm
ACBP Kelch_ Kelch_3 Kelch_3 Kelch Lebercilin
XP_014627431_K_Gm
ACBP Kelch_ Kelch_3 Kelch_3 Kelch
XP_021993755_K_Ha
ACBP Kelch Kelch_3 Kelch_3 Kelch ATG16
XP_022009044_K_Ha
ACBP Kelch_ Kelch_3 Kelch_3 Kelch ATG16
XP_022009045_K_Ha
ACBP Kelch_ Kelch_3 Kelch_3 Kelch ATG16
XP_012089029_K_Jc
ACBP Kelch Kelch_ Kelch_3 Kelch_3 Kelch CENP-F_leu_zip
XP_022893607_K_Oe
ACBP Kelch Kelch_3 Kelch_3 Kelch DUF4670 superfamily
XP_022895969_K_Oe
ACBP Kelch Kelch_3 Kelch_3 Kelch SMC_N superfamily
XP_022895970_K_Oe
ACBP Kelch Kelch_3 Kelch_3 Kelch SMC_N superfamily
XP_022895971_K_Oe
ACBP Kelch Kelch_3 Kelch_3 Kelch SMC_N superfamily
XP_015629756_K_Os
ACBP Kelch_ Kelch_3 Kelch_3 Kelch TPR_MLP SMC_N superfamily
Os03g0835600_K_Os
ACBP Kelch_ Kelch_3 Kelch_3 Kelch SMC_N superfamily
ABF99749_K_Os
ACBP Kelch_ Kelch_3 Kelch_3 Kelch
AFZ62126_K_Vf
ACBP Kelch_ Kelch_6 Kelch_3 Kelch_3 Kelch IncA
AQK61747_K_Zm
ACBP Kelch_ Kelch_ Kelch_3 Kelch_3 Kelch PspA_IM30 superfamily

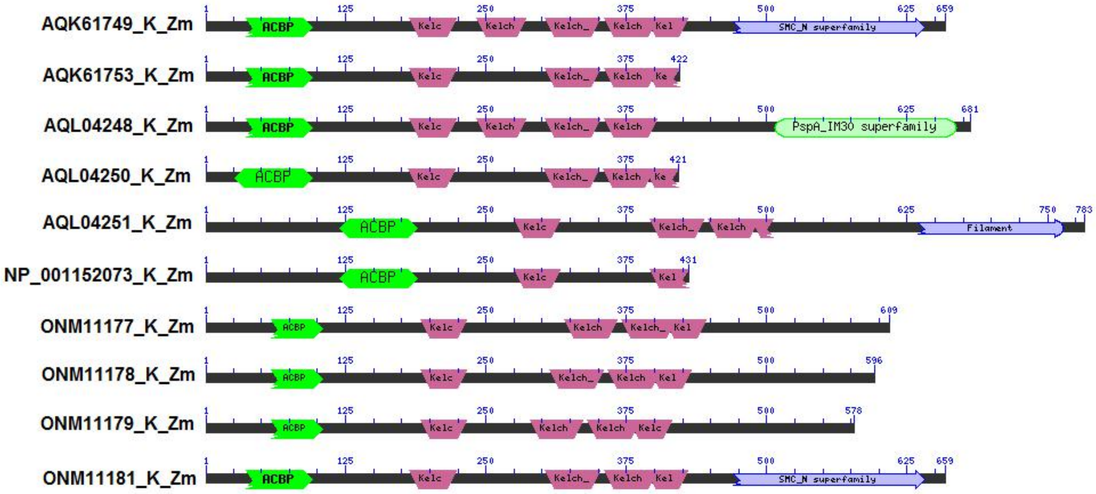

Supplementary figure 2 Domain architecture of ankyrin repeats (A), large (B), and kelch motif (C) ACBP in oil crops. The architecture was generated by using Batch CD-search from NCBI database, using CDSEARCH/oasis_pfam v3 and E-value cut-off of 0.10. ACBD are labeled in green, ankyrin repeats are in blue, and kelch motifs are in purple.
