# Supplementary material for: Functional and Structural Diversity of Acyl-coA Binding Proteins in Oil Crops
Source: Front Genet. 2018 May 22;9:182. doi: 10.3389/fgene.2018.00182 (PMC5972291; doi:10.3389/fgene.2018.00182)
Supplement: Supplementary Figure 3 — Alignment of acyl-coA binding domain in large (A) and kelch motifs (B) ACBP of oil crops. The alignments were performed using Vector NTI software. Underlined capital letter are highly conserved amino acids in ACBD, and leftover are consensual amino acids found in ACBD. [file Image_3.pdf]

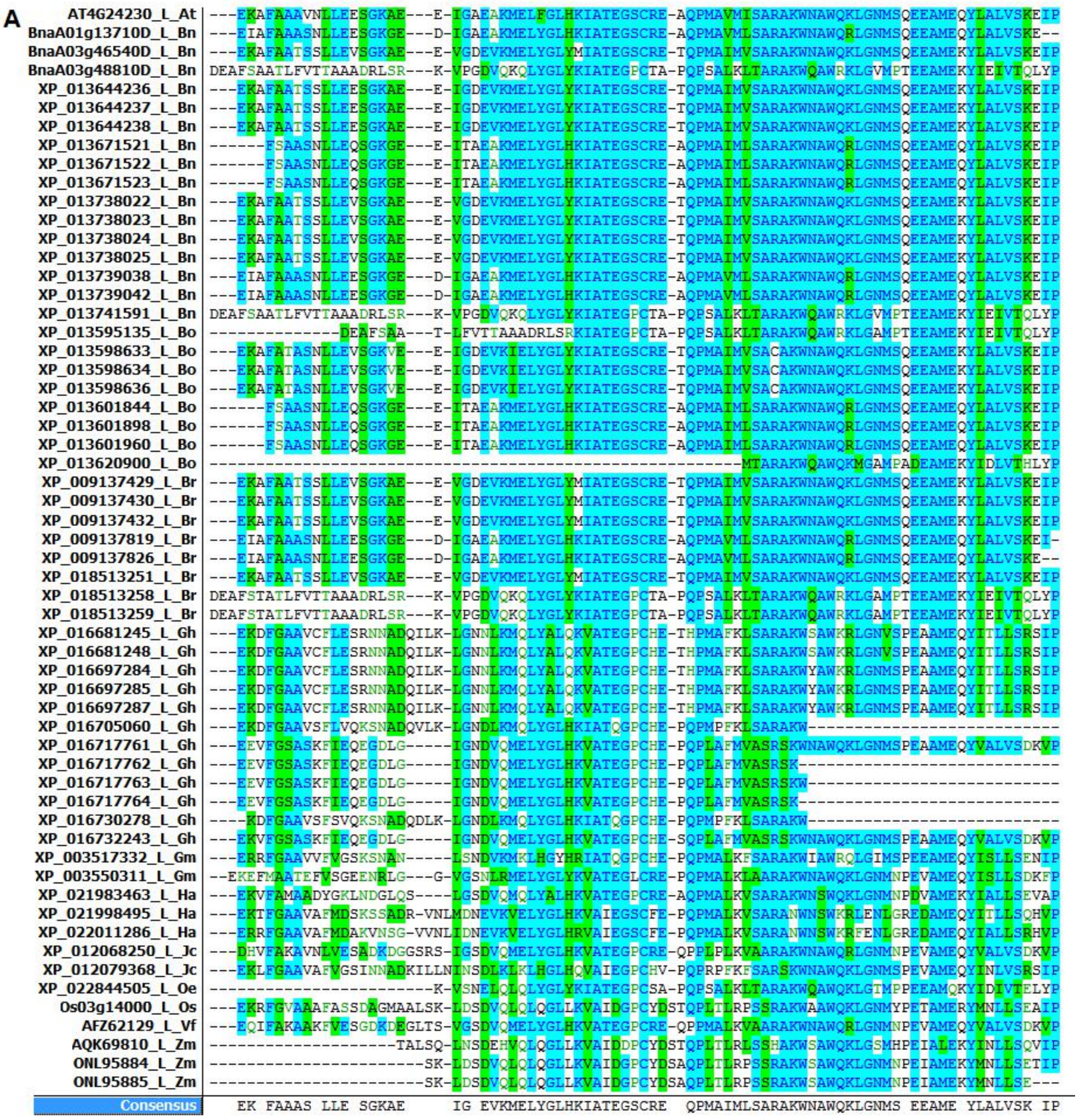
A
AT4G24230_L_At
BnaA01g13710D_L_Bn
BnaA03g46540D_L_Bn
BnaA03g48810D_L_Bn
XP_013644236_L_Bn
XP_013644237_L_Bn
XP_013644238_L_Bn
XP_013671521_L_Bn
XP_013671522_L_Bn
XP_013671523_L_Bn
XP_013738022_L_Bn
XP_013738023_L_Bn
XP_013738024_L_Bn
XP_013738025_L_Bn
XP_013739038_L_Bn
XP_013739042_L_Bn
XP_013741591_L_Bn
XP_013595135_L_Bo
XP_013598633_L_Bo
XP_013598634_L_Bo
XP_013598636_L_Bo
XP_013601844_L_Bo
XP_013601898_L_Bo
XP_013601960_L_Bo
XP_013620900_L_Bo
XP_009137429_L_Br
XP_009137430_L_Br
XP_009137432_L_Br
XP_009137819_L_Br
XP_009137826_L_Br
XP_018513251_L_Br
XP_018513258_L_Br
XP_018513259_L_Br
XP_016681245_L_Gh
XP_016681248_L_Gh
XP_016697284_L_Gh
XP_016697285_L_Gh
XP_016697287_L_Gh
XP_016705060_L_Gh
XP_016717761_L_Gh
XP_016717762_L_Gh
XP_016717763_L_Gh
XP_016717764_L_Gh
XP_016730278_L_Gh
XP_016732243_L_Gh
XP_003517332_L_Gm
XP_003550311_L_Gm
XP_021983463_L_Ha
XP_021998495_L_Ha
XP_022011286_L_Ha
XP_012068250_L_Jc
XP_012079368_L_Jc
XP_022844505_L_Oe
Os03g14000_L_Os
AFZ62129_L_Vf
AQK69810_L_Zm
ONL95884_L_Zm
ONL95885_L_Zm
Consensus
EK FAAAS LLE SGKAE IG EVKMELYGLHKIATEGSCRE QPMAIMLSARAKWNAWQKLGNMS EEAME YLALVSK IP

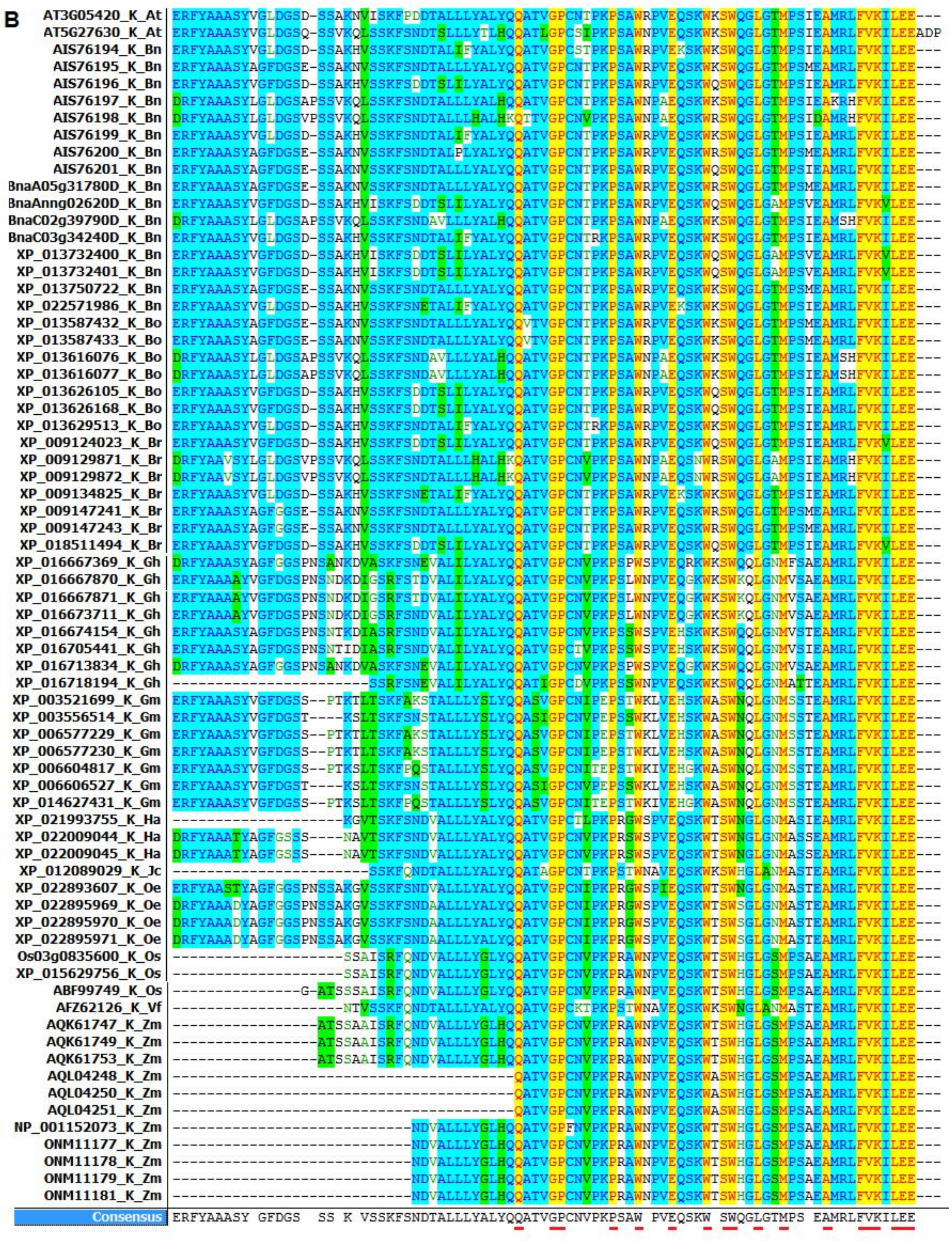

Supplementary figure 3 Alignment of acyl-coA binding domain in large (A) and kelch motifs (B) ACBP of oil crops. The alignments were performed using Vector NTI software. Underlined capital letter are highly conserved amino acids in ACBD, and leftover are consensual amino acids found in ACBD.
