# Supplementary material for: Functional and Structural Diversity of Acyl-coA Binding Proteins in Oil Crops
Source: Front Genet. 2018 May 22;9:182. doi: 10.3389/fgene.2018.00182 (PMC5972291; doi:10.3389/fgene.2018.00182)
Supplement: Supplementary Figure 4 — Secondary structure of ACBP in oil crops. The prediction was made using GOR version IV (Garnier et al., 1996; Combet et al., 2000). [file Image_4.PDF]

Small ACBP

AT1G31812_S_At

```
        10        20        30        40        50        60        70
         |         |         |         |         |         |         |
MGLKEEFEEHAEKVNTLTELPSNEDLLILYGLYKQAKFGPVDTSRPGMFSMKERAKWDAWKAVEGKSSEE
cccccchhhhhhhhhhhccccccchhhhhhhhhhcccccccccccccceeeeeeecchhhhhhhccccchh
AMNDYITKVKQLLEVAASKAST
hhhhhhhhhhhhhhhhhcceec

Sequence length :    92

GOR4 :
   Alpha helix      (Hh) :    47 is  51.09%
   3₁₀ helix        (Gg) :     0 is   0.00%
   Pi helix         (Ii) :     0 is   0.00%
   Beta bridge      (Bb) :     0 is   0.00%
   Extended strand  (Ee) :     9 is   9.78%
   Beta turn        (Tt) :     0 is   0.00%
   Bend region      (Ss) :     0 is   0.00%
   Random coil      (Cc) :    36 is  39.13%
   Ambiguous states (?)  :     0 is   0.00%
   Other states          :     0 is   0.00%
```

BnaA09g24910D_S_Bn

```
        10        20        30        40        50        60        70
         |         |         |         |         |         |         |
MYIYLQEEFEEHAEEVKTLTTKPSDADLLILYGLFKQATVGPVTTSRPGMFSMKERAKWDAWKAVEAKST
ccccccchhhhhhhhhhhhccccccchhhhhhhhhcccccccceecccccceeeeeeecchhhhhhhhhhcc
DEAMSDYITKVKQLMEAAEASAAST
hhhhhhhhhhhhhhhhhhhhheeec

Sequence length :    95

GOR4 :
   Alpha helix      (Hh) :    52 is  54.74%
   3₁₀ helix        (Gg) :     0 is   0.00%
   Pi helix         (Ii) :     0 is   0.00%
   Beta bridge      (Bb) :     0 is   0.00%
   Extended strand  (Ee) :    12 is  12.63%
   Beta turn        (Tt) :     0 is   0.00%
   Bend region      (Ss) :     0 is   0.00%
   Random coil      (Cc) :    31 is  32.63%
   Ambiguous states (?)  :     0 is   0.00%
   Other states          :     0 is   0.00%
```

XP_013583838_S_Bo

```
         10        20        30        40        50        60        70
          |         |         |         |         |         |         |
MGLKEEFEEHAEKVKTLTTKPSDADLLILYGLFKQATVGPVTTSRPGMFSMKERAKWDAWKAVETKSTDE
cccccchhhhhhhhhhhccccccchhhhhhhhhcccccccеeccccceeeeeeeccchhhhhhhhhcccch
AMSDYITKVKQLMEAAEASAAST
hhhhhhhhhhhhhhhhhhheeec

Sequence length :    93

GOR4 :
   Alpha helix      (Hh) :    49 is  52.69%
   3₁₀ helix        (Gg) :     0 is   0.00%
   Pi helix         (Ii) :     0 is   0.00%
   Beta bridge      (Bb) :     0 is   0.00%
   Extended strand  (Ee) :    12 is  12.90%
   Beta turn        (Tt) :     0 is   0.00%
   Bend region      (Ss) :     0 is   0.00%
   Random coil      (Cc) :    32 is  34.41%
   Ambiguous states (?)  :     0 is   0.00%
   Other states          :     0 is   0.00%
```

XP_009108102_S_Br

```
         10        20        30        40        50        60        70
          |         |         |         |         |         |         |
MGLKEDFEEHAENVKKLTTSPSNEDLLILYGLYKQATVGPVTTSRPGMFSMKERAKWDAWKAVEGKSTDE
cccccchhhhhhhhhhhccccccchhhhhhhhhccccccceecccccеeeeeeeccchhhhhhhcccccch
AMSDYITKVKQLLEAEAAAAST
hhhhhhhhhhhhhhhhhhhhhc

Sequence length :    92

GOR4 :
   Alpha helix      (Hh) :    49 is  53.26%
   3₁₀ helix        (Gg) :     0 is   0.00%
   Pi helix         (Ii) :     0 is   0.00%
   Beta bridge      (Bb) :     0 is   0.00%
   Extended strand  (Ee) :     9 is   9.78%
   Beta turn        (Tt) :     0 is   0.00%
   Bend region      (Ss) :     0 is   0.00%
   Random coil      (Cc) :    34 is  36.96%
   Ambiguous states (?)  :     0 is   0.00%
   Other states          :     0 is   0.00%
```

XP_016755542_S_Gh

```
       10        20        30        40        50        60        70
        |         |         |         |         |         |         |
MGLKEEFEEHAEKVKTLPAAPSNDDMLILYGLYKQATVGPVNTSRPGMFNMREKYKWDAWKAVEGKSKEE
cccccchhhhhhhhhhcccccccchhhhhhhhhccccccccccccccceeeeeechhhhhhhhhchhhhh
AMGDYITKVKQLFEAAGSS
hhhhhhhhhhhhhhceeec

Sequence length :    89

GOR4 :
   Alpha helix      (Hh) :    47 is  52.81%
   3_10 helix       (Gg) :     0 is   0.00%
   Pi helix         (Ii) :     0 is   0.00%
   Beta bridge      (Bb) :     0 is   0.00%
   Extended strand  (Ee) :     9 is  10.11%
   Beta turn        (Tt) :     0 is   0.00%
   Bend region      (Ss) :     0 is   0.00%
   Random coil      (Cc) :    33 is  37.08%
   Ambiguous states (?)  :     0 is   0.00%
   Other states          :     0 is   0.00%
```

AJQ20789_S_Ha

```
       10        20        30        40        50        60        70
        |         |         |         |         |         |         |
MGLKEDFEEHAEKVKSLTKKPSDADLLILYGLFKQATVGSVNTDRPGMFSMKERAKWDAWKAVEAKSKEE
ccccccchhhhhhhhhhcccccchhhhhhhhhccccccceeccccccceeeeeeecchhhhhhhhhhhhhh
AMNDYITMAKQLLEADAAAA
hhhhhhhhhhhhhhhcceec

Sequence length :    90

GOR4 :
   Alpha helix      (Hh) :    48 is  53.33%
   3_10 helix       (Gg) :     0 is   0.00%
   Pi helix         (Ii) :     0 is   0.00%
   Beta bridge      (Bb) :     0 is   0.00%
   Extended strand  (Ee) :    11 is  12.22%
   Beta turn        (Tt) :     0 is   0.00%
   Bend region      (Ss) :     0 is   0.00%
   Random coil      (Cc) :    31 is  34.44%
   Ambiguous states (?)  :     0 is   0.00%
   Other states          :     0 is   0.00%
```

ABE72959_S_Jc

```
       10        20        30        40        50        60        70
        |         |         |         |         |         |         |
MGLKEDFEEYAEKAKTLPENTTNENKLILYGLFKQATVGPVNTSRPGMFNMRDRAKWGAWKAVEGKSKEE
cccccchhhhhhhhhcccccccccchhhhhhhcccccccccccccccceeeeeechhhhhhhhhccchhh
AMSDYITKVKLLLEEAAAAASA
hhhhhhhhhhhhhhhhhhhhhc

Sequence length :    92

GOR4 :
   Alpha helix      (Hh) :    49 is   53.26%
   3₁₀  helix       (Gg) :     0 is    0.00%
   Pi helix         (Ii) :     0 is    0.00%
   Beta bridge      (Bb) :     0 is    0.00%
   Extended strand  (Ee) :     6 is    6.52%
   Beta turn        (Tt) :     0 is    0.00%
   Bend region      (Ss) :     0 is    0.00%
   Random coil      (Cc) :    37 is   40.22%
   Ambiguous states (?)  :     0 is    0.00%
   Other states          :     0 is    0.00%
```

XP_022881340_S_Oe

```
       10        20        30        40        50        60        70
        |         |         |         |         |         |         |
MALKVNDLSLLPEEFEEHAEKAKTLPESTSNEDKLVLYGLYKQATVGNVNTSRPGIFNMRDRAKWDAWKA
ccccccccccchhhhhhhhhhhhcccccccccchhhhhhhcccceeccccccccceeeeeeecchhhhhh
VEGKSQEEAMSDYITKVKQLKEAAAA
hhchhhhhhhhhhhhhhhhhhhceec

Sequence length :    96

GOR4 :
   Alpha helix      (Hh) :    46 is   47.92%
   3₁₀  helix       (Gg) :     0 is    0.00%
   Pi helix         (Ii) :     0 is    0.00%
   Beta bridge      (Bb) :     0 is    0.00%
   Extended strand  (Ee) :    11 is   11.46%
   Beta turn        (Tt) :     0 is    0.00%
   Bend region      (Ss) :     0 is    0.00%
   Random coil      (Cc) :    39 is   40.62%
   Ambiguous states (?)  :     0 is    0.00%
   Other states          :     0 is    0.00%
```

Os03g0576600_S_Os

10 20 30 40 50 60 70

MGLQEDFEEYAEKVKTLPESTSNEDKLILYGLYKQATVGDVNTSRPGIFAQRDRAKWDAWKAVEGKSKEE
cccccchhhhhhhhccccccccchhhhhhhhhcccceeccccccccceeeechhhhhhhhhhhhccccch
AMSDYITKVKQLQEEAAALKAVFRAYLVGEMNIFECHIGRLTRCRRGFRTQMKKQIVYSPGTREMNLLSL
hhhhhhhhhhhhhhhhhhhhhhhhhhhhcccceeeeeecccccccccccccceeeeeeccccchhhhhhh
IKPSLAHVGYCSTYG
cccccceeceeeeec

Sequence length : 155

GOR4 :

Alpha helix	(Hh) :	65	is	41.94%
3_{10} helix	(Gg) :	0	is	0.00%
Pi helix	(Ii) :	0	is	0.00%
Beta bridge	(Bb) :	0	is	0.00%
Extended strand	(Ee) :	25	is	16.13%
Beta turn	(Tt) :	0	is	0.00%
Bend region	(Ss) :	0	is	0.00%
Random coil	(Cc) :	65	is	41.94%
Ambiguous states	(?) :	0	is	0.00%
Other states	:	0	is	0.00%

AFZ62125_S_Vf

10 20 30 40 50 60 70

MGLKEEFEEYAEKAKTLPENTTNENKLILYGLYKQATVGPVNTSRPGIFNQRDRAKWDAWKAVEGKSKEE
cccccchhhhhhhhhcccccccccchhhhhhhcccccccccccccccceccccccchhhhhhhhhccccch
AMSDYITKVKQLLEEAAAAAS
hhhhhhhhhhhhhhhhhhhhc

Sequence length : 91

GOR4 :

Alpha helix	(Hh) :	46	is	50.55%
3_{10} helix	(Gg) :	0	is	0.00%
Pi helix	(Ii) :	0	is	0.00%
Beta bridge	(Bb) :	0	is	0.00%
Extended strand	(Ee) :	1	is	1.10%
Beta turn	(Tt) :	0	is	0.00%
Bend region	(Ss) :	0	is	0.00%
Random coil	(Cc) :	44	is	48.35%
Ambiguous states	(?) :	0	is	0.00%
Other states	:	0	is	0.00%

AQK41346_S_Zm

```
        10        20        30        40        50        60        70
         |         |         |         |         |         |         |
MGLQEEFEEHAEKAKTLPESTSNENKLILYGLYKQATVGDVNTDRPGIFYQKDRAKWDAWKAVEASQGIT
ccccchhhhhhhhhhcccccccccchhhhhhccccceeccccccccchhhhhhhhhhhhhhhhhhhcchh
VLLAQTLEILAGKSKDEAMNDYITKVKQLQEEAAAS
hhhhhhhhhhhhhchhhhhhhhhhhhhhhhhhhceec
```

Sequence length : 106

GOR4 :

Alpha helix	(Hh)	:	68	is	64.15%
3_{10} helix	(Gg)	:	0	is	0.00%
Pi helix	(Ii)	:	0	is	0.00%
Beta bridge	(Bb)	:	0	is	0.00%
Extended strand	(Ee)	:	4	is	3.77%
Beta turn	(Tt)	:	0	is	0.00%
Bend region	(Ss)	:	0	is	0.00%
Random coil	(Cc)	:	34	is	32.08%
Ambiguous states	(?)	:	0	is	0.00%
Other states		:	0	is	0.00%

Ankyrin repeats ACBP

AT4G27780_A_At

```
        10        20        30        40        50        60        70
         |         |         |         |         |         |         |
MGDWAQLAQSVILGLIFSYLLAKLISIVVTFKEDNLSLTRHPEESQLEIKPEGVDSRRLDSSCGGFGGEA
cccccchhhhhhhhhhhhhhhhhhheeeeecccccccccccchhhhhhcccccccceeeccccccccccc
DSLVAEQGSSRSDSVAGDDSEEDDDWEGVESTELDEAFSAATLFVTTAAADRLSQKVPSDVQQQLYGLYK
chhhhcccccccceeccccccccccchhhhchhhhhhhhhhhhhhhhhhhhhhhhcccccchhhhhhhhee
IATEGPCTAPQPSALKMTARAKWQAWQKLGAMPPEEAMEKYIEIVTQLYPTWLDGGVKAGSRGGDDAASN
ecccccccccccccchhhhhhhhhhhhhccccccchhhhhhhhheeeeecccccccceeeccccccccccc
SRGTMGPVFSSLVYDEESENELKIDAIHGFAREGEVENLLKSIESGIPVNARDSEGRTPLHWAIDRGHLN
ccccccceeeeeccchhhhhhhhhhhhhccchhhhhhhhhhhhcccccccccccccccceeeeecccccc
IAKVLVDKNADVNAKDNEGQTPLHYAVVCDREAIAEFLVKQNANTAAKDEDGNSPLDLCESDWPWIRDSA
hhhhhhhhcccccccccccccceeeeehhhhhhhhhhhhhhhhchhhhccccccceeecccccccccccc
KQAD
ccee

Sequence length :   354

GOR4 :
   Alpha helix      (Hh) :   140 is   39.55%
   3₁₀ helix        (Gg) :     0 is    0.00%
   Pi helix         (Ii) :     0 is    0.00%
   Beta bridge      (Bb) :     0 is    0.00%
   Extended strand  (Ee) :    41 is   11.58%
   Beta turn        (Tt) :     0 is    0.00%
   Bend region      (Ss) :     0 is    0.00%
   Random coil      (Cc) :   173 is   48.87%
   Ambiguous states (?)  :     0 is    0.00%
   Other states          :     0 is    0.00%
```

XP_013738044_A_Bn

```
        10        20        30        40        50        60        70
         |         |         |         |         |         |         |
MGDWAQLAQSVIIGLIFSYLLAKLISIVVTFKEDNLSLTRHHDPEPESKNLKPEVDSRRIESSTGEADSL
ccccccchhhhhhhhhhhhhhhhhheeeeecccccccceecccccccccccccccccceeeecccccchhh
VAEQGSSRGDSVAGDTEDEDDDDWEGVESTELDEAFSAATLFVTTAASDRLSQKVPSEVLQQLYGLYKIA
hhhcccccceeeccccccccccchhhhchhhhhhhhhhhhhhhhhhhhhhhhcccchhhhhhhhhheeec
TEGPCTAPQPSALKITARAKWQAWQKLGAMPPEEAMEKYIEIVTQLYPTWLDGGVKAGSGSKDEAVSNTG
cccccccccccchhhhhhhhhhhhhccccccchhhhhhhhheeeeecccccccceeccccccceeeeccc
GTMGPVFSSLVYEEESEIELKIDAIHEFAREGEVENLLKSIESGMPVNAKDSEGRTPLHWAIDRGHLDIA
ccccceecccccchhhhhhhhhhhhhhhhhhhhhhhhhhhhhhcccccccccccccccceeeeecccccchhh
KLLVDKNADVNAKDNEGQTPLHYAVVCDREAIAEFLVKQKANTASKDDDGNSPLDLCESDWPWLRETAKQ
hhhhhhcccccccccccccceeeeehhhhhhhhhhhhhhhhcccccccccccceeeccccccchhhhcccc
TD
ee

Sequence length :   352

GOR4 :
   Alpha helix      (Hh) :   143 is  40.62%
   3₁₀  helix       (Gg) :     0 is   0.00%
   Pi helix         (Ii) :     0 is   0.00%
   Beta bridge      (Bb) :     0 is   0.00%
   Extended strand  (Ee) :    45 is  12.78%
   Beta turn        (Tt) :     0 is   0.00%
   Bend region      (Ss) :     0 is   0.00%
   Random coil      (Cc) :   164 is  46.59%
   Ambiguous states (?)  :     0 is   0.00%
   Other states          :     0 is   0.00%
```

XP_013601590_A_Bo

```
         10        20        30        40        50        60        70
          |         |         |         |         |         |         |
MGDWAQLAQSVIIGLIFSYLLAKLISIVVTFKEDNLSLTRHHDPEPGLKNLKPEVDSRRIESSTGEADSL
ccccccchhhhhhhhhhhhhhhhhheeeeecccccccceeeccccccccccccccccceeeeccccccchhh
VAEQGSSRGDSVAGDTEDEDDDWEGVESTELDEAFSAATLFVTTAASDRLSQKVPSEVQQQLYGLYKIAT
hhhcccccceeecccccccccchhhhchhhhhhhhhhhhhhhhhhhhhhhhhcccchhhhhhhhhheeecc
EGPCTAPQPSALKITARAKWQAWQKLGAMPPEEAMEKYIEIVTQLYPTWLDGGVKAGSASKDEAVSNTGG
ccccccccccchhhhhhhhhhhhhccccccchhhhhhhhheeeeecccccccccccccccccceeeecccc
TMGPVFSSLVYQEESENELKIDAIHEFAREGEVENLLKSIESGIPVNAKDSEGRTPLHWAIDRGHFEIAK
cccceecccccchhhhhhhhhhhhhhhhhhhhhhhhhhhhhccccccccccccccceeeeecccchhhhh
LLFDKNADVNAKDNEGQTPLHYAVVCDREAIAEFLVKQKANTTSKDDDGNTPLDLCESDWPWLRETANQT
hhhhhchhhhccccccccceeeeehhhhhhhhhhhhhhcccccccccccccceeeccccccchhhhcccee
D
c
```

Sequence length : 351

GOR4 :

Alpha helix	(Hh) :	145	is	41.31%
3_{10} helix	(Gg) :	0	is	0.00%
Pi helix	(Ii) :	0	is	0.00%
Beta bridge	(Bb) :	0	is	0.00%
Extended strand	(Ee) :	44	is	12.54%
Beta turn	(Tt) :	0	is	0.00%
Bend region	(Ss) :	0	is	0.00%
Random coil	(Cc) :	162	is	46.15%
Ambiguous states	(?) :	0	is	0.00%
Other states	:	0	is	0.00%

XP_009143973_A_Br

```
        10        20        30        40        50        60        70
         |         |         |         |         |         |         |
MGDWAQLAQSVIIGLIFSYLLAKLISIVVTFKEDNLSLTRHHDPEPESKNLKPEVDSRRIESSTDEADSL
ccccccchhhhhhhhhhhhhhhhhhheeeeecccccccceecccccccccccccccccccceecccccchhhhh
VAEQGSSRGDSVAGDTEEDDDDWEGVESTELDEAFSAATLFVTTAASDRLSQKVPSEVQQQLYGLYKIVT
hhhcccccccceecccccccccccccccchhhhhhhhhhhhhhhhhhhhhhcccchhhhhhhhhheeeee
EGPCTAPQPSALKITARAKWQAWQKLGAMPPEEAMEKYIEIVTQLYPTWLDGGVKAGSGSKDEAVSNTGG
ccccccccccchhhhhhhhhhhhhccccccchhhhhhhhheeeeecccccccceecccccccceeeecccc
TMGPVFSSLVYEEESENELKIDAIHEFAREGEVESLLKSIESGIPVNAKDSEGRTPLHWAIDRGHFEIAK
cccceecccccchhhhhhhhhhhhhhhhhhhhhhhhhhhhhccccccccccccccccceeeeecccchhhhh
LLVDKNADVNAKDNEGQTPLHYAVVCDREAIAEFLVKQKANTASKDDDGNSPVDLCESDWPWLRETAKQT
hhhhhcccccccccccccceeeeehhhhhhhhhhhhhhhhcccccccccccceeeeccccchhhhcccce
D
e

Sequence length :   351

GOR4 :
   Alpha helix      (Hh) :   139 is  39.60%
   3_10 helix       (Gg) :     0 is   0.00%
   Pi helix         (Ii) :     0 is   0.00%
   Beta bridge      (Bb) :     0 is   0.00%
   Extended strand  (Ee) :    45 is  12.82%
   Beta turn        (Tt) :     0 is   0.00%
   Bend region      (Ss) :     0 is   0.00%
   Random coil      (Cc) :   167 is  47.58%
   Ambiguous states (?)  :     0 is   0.00%
   Other states          :     0 is   0.00%
```

XP_016670800_A_Gh

```
         10        20        30        40        50        60        70
          |         |         |         |         |         |         |
MSGFWLIDSMADWQQLLQSIILGLIFSYLLAKLISLVVSFKDDNLSITRARSSNVQGYEKIDDSGSGSDP
cccceeecccchhhhhhhhhhhhhhhhhhhhhhhheeeeccccccceeeecccccccceeeeccccccccc
IGSTEHLGLHKSDSVVAELGSIRAESDGDGDGVDDDDDDWEGVECTELDDAFSAATAFVAAAAADRLSQK
ccccccccccccchhhhhhccceeecccccccceecccccccccccccchhhhhhhhhhhhhhhhhhhhhc
VPNEVQLQLYGLYKVATEGPCTAPQPSALKMTARAKWQAWHKLGAMPPEDAMQKYIDVVTELYPSWAAGS
ccchhhhhhhhheeecccccccccccccchhhhhhhhhhhhhccccccchhhhhhheeeeeeeecccchhhh
AMGKGGADNASSKDVKGPMGPVFSSFVYEEESENDLKMDAIHTFAREGELDNLLKCIESGVSVHLQDSEG
cccccccccccccccccccccceeccccchhhhhhhhhhhhhhhhhhhchhhhhhhcccccceeeeccccc
RTPMHWAVDRGHLKIAEALLSRNDDVNAMDNEGQTPLHYAVMCEREDIAKLLVKQNADKDTKDNDGNSPV
ccceeeeeecccchhhhhhhhhcccchhhhccccccchhhhhhhhhhhhhhhhhhhhcccccccccccccce
NLCDSDWPWLQRAGKAE
eeecccccchhhhcceec
```

```
Sequence length :   367
```

```
GOR4 :
   Alpha helix      (Hh) :   146 is  39.78%
   310 helix        (Gg) :     0 is   0.00%
   Pi helix         (Ii) :     0 is   0.00%
   Beta bridge      (Bb) :     0 is   0.00%
   Extended strand  (Ee) :    47 is  12.81%
   Beta turn        (Tt) :     0 is   0.00%
   Bend region      (Ss) :     0 is   0.00%
   Random coil      (Cc) :   174 is  47.41%
   Ambiguous states (?)  :     0 is   0.00%
   Other states          :     0 is   0.00%
```

KRH53542_A_Gm

```
        10        20        30        40        50        60        70
         |         |         |         |         |         |         |
MAEWQSLLQSILVGLIFSYLLAKLISIVVSFKDDNLTVTRASAAETTATRAEQFEDALKRDDAVSSDARP
cccccchhhhhhhhhhhhhhhhhhhheeeeecccccchhhhhhhhhhhhhhhhhhhhhhhhcccccccccc
FEEESVVAEHGSVRIDSDGDYDDDWEGVESTELDEAFSAATAFVAAAAADRLSQKVSSDVQLQLYGLYKI
cchhhhhhcccceeeccccccccccccccchhhhhhhhhhhhhhhhhhhhhhhhcccchhhhhhhhheee
ATEGPCSTPQPSPLKMTARAKWQAWQKLGAMPPEDAMQKYIDIVTEIYPTWLDGSSLRNKSGDSGGHGSE
cccccccccccccchhhhhhhhhhhhccccccchhhhhhhhhhhhhhcccccccccccccccccccccee
AKGPMGPVFSTFVYEEEYGSDSQMEAIHGFAREGDMANLLKCIENGVSMNLKDSEGRTPLHWAVDRGHLN
cccccccceeceeeeeecccccchhhhhhhhhhhhchhhhhhhhhcccceeccccccccccceeeeecccccc
VTELLVGKNADVNAKDNDGQTPLHYAVTCEREAIAEYLVKHNADIYSKDNDGSSPRDICESNWPCMQHVG
hhhhhhcccccccccccccccceeechhhhhhhhhhhhhhcccceeecccccccceeeeeccccccceee
EEVN
eeec
```

```
Sequence length :   354
```

```
GOR4 :
   Alpha helix      (Hh) :   149 is  42.09%
   3₁₀ helix        (Gg) :     0 is   0.00%
   Pi helix         (Ii) :     0 is   0.00%
   Beta bridge      (Bb) :     0 is   0.00%
   Extended strand  (Ee) :    45 is  12.71%
   Beta turn        (Tt) :     0 is   0.00%
   Bend region      (Ss) :     0 is   0.00%
   Random coil      (Cc) :   160 is  45.20%
   Ambiguous states (?)  :     0 is   0.00%
   Other states          :     0 is   0.00%
```

XP_022012478_A_Ha

```
        10        20        30        40        50        60        70
         |         |         |         |         |         |         |
MFDWQQYTQSILFGLIFSFLLAKLFSIIFSFRDQNLRITRASDANDDDEDESVEVSEIHNDYDFNKEKQP
ccccccccchhhhhhhhhhhhhhhhhhhhhhhhcchhhhhhccccccccchhhhhhhhcccccccccccc
LIDREKESGEVYSDEEDSDDDWEGVESTELDEAFSAATAFVAATVADRSAQKVSSDLQLQLYGLYKIATE
hhhhhccccceeeccccccchhhhhchhhhhhhhhhhhhhhhhhhhhhhhhhhhhhhhhhhhheeeccc
GPCSVPQPSALKMTARAKWNAWQKLGAMPPEEAMQKYIEIITELYPTWLAGSTSKKRDENANERSSNENR
cccccccccchhhhhhhhhhhhhccccccchhhhhhhhhhhhhhccccccccccchhhhhhhhhcccccc
PMGPVFSSFIHEEESDELKLDAIHAFAREGDTENLIKCVEAGIPVDIKDSEGRAPLHWAVDRGHIEAAEL
ccccccccccchhhhhhhhhhhhhhhhhhhccchhhhhhhcccccccccccccccchhhhhccchhhhhhh
LLSRNADVNLKDNEGQTPLHYAAVCEREHIAELLVKKNASTDIKDDDGNYPADLCDSKWPWIQPQAPSL
hhhhhhhhhccccccccchhhhhhhhhhhhhhhhhhhccccceeccccccceeeccccccccccccccee
```

```
Sequence length :   349
```

```
GOR4 :
   Alpha helix     (Hh) :   187 is  53.58%
   3₁₀ helix       (Gg) :     0 is   0.00%
   Pi helix        (Ii) :     0 is   0.00%
   Beta bridge     (Bb) :     0 is   0.00%
   Extended strand (Ee) :    13 is   3.72%
   Beta turn       (Tt) :     0 is   0.00%
   Bend region     (Ss) :     0 is   0.00%
   Random coil     (Cc) :   149 is  42.69%
   Ambiguous states (?) :     0 is   0.00%
   Other states         :     0 is   0.00%
```

KDP35833_A_Jc

```
        10        20        30        40        50        60        70
         |         |         |         |         |         |         |
MGDWQQLLQSILIGLLFSYLLAKLISVLVSFKDDNLTISRSSESHLSHPPRPKSNPDYGLRARDMAAAGD
cccccchhhhhhhhhhhhhhhhhhhheeeeccccccеecccccccccccccccccccchhhhhhhhccc
VSTLFDTDSMMAEHGSVRNESTAGSEFGDNDIDDDDWEGVESTELDETFSAATAFVAAAAADRLSQKVST
ceeeccchhhhhhhccccccccccccccccccccccccccccchhhhhhhhhhhhhhhhhhhhhhhcccc
DVQLQLYALYKIATEGPCSTPPPSALKMSARAKWQAWQKLGAMPPEDAMQKYIDIVTELYPSWASGSAMK
hhhhhhhhheeeccccccccccccchhhhhhhhhhhhhccccccchhhhhhhhheeeeecccccchhhhh
RKDGDGDARSSDVKASMGPVFSTFVFEEESGNESKLEDIHAFAREGDVKNLLNCIESGASVNLKDSEGRT
hcccccccceecccccccccеeeeeecccccccchhhhhhhhhhhcchhhhhhcccccceeccccccccc
PLHWAVDRGHLNVVDVLVGMSADINAKDIDGQTPLHYAAVCDREDIAEFLIRQNADTNLKDNDGKSARDL
ceeeeecccccceeeeeeccchhhcccccccccchhhhhhcchhhhhhhhhhhccccccccccccchhh
CEFDWRCLRLPATQTE
hhhhhccccccceeec

Sequence length :   366

GOR4 :
   Alpha helix      (Hh) :   140 is  38.25%
   3₁₀ helix        (Gg) :     0 is   0.00%
   Pi helix         (Ii) :     0 is   0.00%
   Beta bridge      (Bb) :     0 is   0.00%
   Extended strand  (Ee) :    41 is  11.20%
   Beta turn        (Tt) :     0 is   0.00%
   Bend region      (Ss) :     0 is   0.00%
   Random coil      (Cc) :   185 is  50.55%
   Ambiguous states (?)  :     0 is   0.00%
   Other states          :     0 is   0.00%
```

XP_022875290_A_Oe

```
        10        20        30        40        50        60        70
         |         |         |         |         |         |         |
MCIIMAEWQQFVQSVFFGVIFSFLLAKLFSVIFAYRDENLRNARSVSPEPASKPAEETVASLSRGLGESK
ccccccccccccceeeccccchhhhhhhhhhhhhhhhhhhhhhhccccccccccccccchhhhhhhhhcccccc
PLIVENEGLARESSTVGDLTDSDDDWEGVESTELDEAFSAATAFVAATAADSSAQNVSNEVQLQLYGFYK
chhhhhcccccccceeceeeccccccccccccchhhhhhhhhhhhhhhhhccchhhhhhhhhhhhhhceee
IATEGPCSAAQPSLLKPTARAKWQAWQKLGAMPPEEAMEKYIDIVTTLYPSWAMGSGTKSRGEESSDAPN
eccccccccccccccccchhhhhhhhhccccccchhhhhhhheeeeeeeccccccccccccccccccccc
AGSKGPMGPVFSSFIYEEDSESELKFDAIHAFAREGDEEKLLKCIESGVPADLRDSEGRTPLHWAVDRGH
ccccccccceeceeecccccchhhhhhhhhhhhhhhcchhhhhhhhcccccccccccccccceeeeecccc
MNITELLLKKNADVNAKDDEGQTPLHYAVVCERAAIAELLVKNNADTKIKDNDGDSPCELCESSWPWMQQ
hhhhhhhhhhhhhhcccccccccchhhhhhhhhhhhhhhhhhccccceeecccccccceeeeeccccceee
ISTSADD
eeeeeec

Sequence length :   357

GOR4 :
   Alpha helix      (Hh) :   140 is  39.22%
   3_10 helix       (Gg) :     0 is   0.00%
   Pi helix         (Ii) :     0 is   0.00%
   Beta bridge      (Bb) :     0 is   0.00%
   Extended strand  (Ee) :    46 is  12.89%
   Beta turn        (Tt) :     0 is   0.00%
   Bend region      (Ss) :     0 is   0.00%
   Random coil      (Cc) :   171 is  47.90%
   Ambiguous states (?)  :     0 is   0.00%
   Other states          :     0 is   0.00%
```

Os04g0681900_A_Os

```
        10        20        30        40        50        60        70
         |         |         |         |         |         |         |
MGGDWQELAQAAVIGLLFAFLVAKLISTVIAFKEDNLRITRSTPTSPSAADTPAAPAPPPASLDGGHGDT
ccccchhhhhhhhhhhhhhhhhhhhhhhhhhhhhhccceeeecccccccccccccccccccccccccccccc
SDGSGSDSDSDWEGVESTELDEEFSAASAFVAASAASGTSVPEQAQLQLYGLYKIATEGPCTAPQPSALK
cccccccccccccccccchhhhhhhhhhhhhhhhhcccccchhhhhhhhhhheeeccccccccccchhhh
LKARAKWNAWHKLGAMPTEEAMQKYITVVDELFPNWSMGSSTKRKDEDTTVSASSSKGPMGPVFSSLMYE
hhhhhhhhhhhhcccccchhhhhhheeeeeeecccccccccccccccceeeeeecccccccceechhhhh
EEDQGNDSELGDIHVSAREGAIDDIAKHLAAGVEVNMRDSEGRTPLHWAVDRGHLNSVEILVNANADVNA
hccccccccccchhhhhhhhhhhhhhhhhhhhhhhhccccccccceeeeeccccccchhhhhhccccccc
QDNEGQTALHYAVLCEREDIAELLVKHHADVQIKDEDGNTVRELCPSSWSFMNLAN
ccccchhhhhhhhhhhhhhhhhhhhhhccceeeecccccceeeeeccccceeeceec
```

```
Sequence length :   336
```

```
GOR4 :
   Alpha helix      (Hh) :   138 is   41.07%
   3₁₀  helix       (Gg) :     0 is    0.00%
   Pi helix         (Ii) :     0 is    0.00%
   Beta bridge      (Bb) :     0 is    0.00%
   Extended strand  (Ee) :    41 is   12.20%
   Beta turn        (Tt) :     0 is    0.00%
   Bend region      (Ss) :     0 is    0.00%
   Random coil      (Cc) :   157 is   46.73%
   Ambiguous states (?)  :     0 is    0.00%
   Other states          :     0 is    0.00%
```

ACG24390_A_Zm

```
         10        20        30        40        50        60        70
          |         |         |         |         |         |         |
MAGDWQDLGQAAAIGLLFAFLLAKLISTVIAFKEDNLRITRSPPSSPTAAAAAPAPATPPLPSQHDAGIG
ccccccchhhhhhhhhhhhhhhhhhhhhhhhhhhcccccceeeccccccchhhhcccccccccccccccceec
SGSDSDWEGVESTELDEEFSAASAFVAASAASGTSVPEEAQLRLYGLYKIATEGPCTAPQPSALKLKARA
ccccccccccccchhhhhhhhhhhhhhhhhcccccchhhhhhhhhhheeeccccccccccchhhhhhhhh
KWNAWHKLGAMPTEEAMQEYITIVQELFPNWDAGTSAKRKDEDSITSASASKGPMGPVFSSLMYEEDEGN
hhhhhhhcccccchhhhhhhhhhhhhhcccccccccccccccccceeeeecccccccceechhhhhhcccc
DSELGDIHVLAREGATEDIVKFLAAGVEVNMRDTEGRTPLHWAVDRGHLSAVEVLAKANADLNAKDNEGQ
ccccchhhhhhhhcchhhhhhhhhhceeeccccccccccceeeeecccchhhhhhhhhhhhhhhhhccchh
TALHYAAVCEREDIAELLVKHHADLQIKDEDGNTAQDLCPPSWSFMNRAN
hhhhhhhhhhhhhhhhhhhhhcchhhhhcccccceeccccccccceeceec
```

```
Sequence length :   330
```

```
GOR4 :
   Alpha helix      (Hh) :   158 is  47.88%
   3_10 helix       (Gg) :     0 is   0.00%
   Pi helix         (Ii) :     0 is   0.00%
   Beta bridge      (Bb) :     0 is   0.00%
   Extended strand  (Ee) :    29 is   8.79%
   Beta turn        (Tt) :     0 is   0.00%
   Bend region      (Ss) :     0 is   0.00%
   Random coil      (Cc) :   143 is  43.33%
   Ambiguous states (?)  :     0 is   0.00%
   Other states          :     0 is   0.00%
```

Large ACBP

AT4G24230_L_At

```
        10        20        30        40        50        60        70
         |         |         |         |         |         |         |
MEVFLEMLLTAVVALLFSFLLAKLVSVATVENDLSSDQPLKPEIGVGVTEDVRFGMKMDARVLESQRNFQ
cccchhhhhhhhhhhhhhhhhhhhhhhhhhcccccccccccccccceeehhhhhhhhhhhhhhhhhcccc
VVDENVELVDRFLSEEADRVYEVDEAVTGNAKICGDREAESSAAASSENYVIAEEVILVRGQDEQSDSAE
ccccchhhhhhhhhhhhhhhhhhhhhhccccccccccchhhhhhhhhhhhhhhhhhhhhcccccccchhh
AESISSVSPENVVAEEIKSQGQEEVTELGRSGCVENEESGGDVLVAESEEVRVEKSSNMVEESDAEAENE
hhccccccccchhhhhhhhcchhhhhhhccccceeccccccceeeeechhhhhhhccchhhhhhhhhhhh
EKTELTIEEDDDWEGIERSELEKAFAAAVNLLEESGKAEEIGAEAKMELFGLHKIATEGSCREAQPMAVM
hhhheeeecccchhhhhhhhhhhhhhhhhhhhhhhchhhhhhhhhhhhhhhheeeccccccccccchhhh
ISARAKWNAWQKLGNMSQEEAMEQYLALVSKEIPGLTKAGHTVGKMSEMETSVGLPPNSGSLEDPTNLVT
hhhhhhhhhhhhhcchhhhhhhhhhhhhhhhcccceecccccccccccccceeccccccccccccccceee
TGVDESSKNVSGER
eccccccccceeec

Sequence length :   364

GOR4 :
   Alpha helix     (Hh) :   201 is  55.22%
   3_10 helix      (Gg) :     0 is   0.00%
   Pi helix        (Ii) :     0 is   0.00%
   Beta bridge     (Bb) :     0 is   0.00%
   Extended strand (Ee) :    28 is   7.69%
   Beta turn       (Tt) :     0 is   0.00%
   Bend region     (Ss) :     0 is   0.00%
   Random coil     (Cc) :   135 is  37.09%
   Ambiguous states (?)  :     0 is   0.00%
   Other states          :     0 is   0.00%
```

XP_013739038_L_Bn

```
        10         20         30         40         50         60         70
         |          |          |          |          |          |          |
MEFLLELLLTAVVALLFSFLVAKLVSVSMTGVNDRSSDQAGETEIGVGDGSATVEELCFGLKVDAPVVQG
cccchhhhhhhhhhhhhhhhhhhhhhhhhhhccccccccccceeeeecccccchhhhhhccccccccccc
ERKLRVVVGENVEHVDRFGSGADRVVDEVEEAARDVELVVPTTEANEFLAAVSPGNVVAKEMVVRGEEET
ccceeeeecccccceeccccccccchhhhhhhhhhhhhhhcccchhhhhhhhcccccchhhhhhhhccccc
GYEREELVVSTAEAESTASISPENVITEEIMNRGQEEGTEGRSSCVENVERGEVVVTEAEEVRVEESNNW
chhhhhhhhhhhhhhcccccccccchhhhhhhhcccccccccccccccccccchhhhhhhhhhhhhccch
EKSEDKMELSIEGQVELSIEEEDLDDDWEGIERSELEIAFAAASNLLEESGKGEDIGAEAKMELYGLHKI
hhhhhhhhhhhhhhhhhhhhccccccchhhhhhhhhhhhhhhhhhhhhhccccchhhhhhhhhhhhheee
ATEGSCREAQPMAVMLSARAKWNAWQRLGNMSQEEAMEQYLALVSKEIPDLVNTVGKMPETETSVDLPPN
ccccccccccchhhhhhhhhhhhhhhhhcchhhhhhhhhhhhhhhhcccceeccccccccccceecccccc
SGSLEDPTTLDTIGVATSKNEIHVSGEDESSV
ccccccccceeeeeecccccceeeecccceec

Sequence length :   382

GOR4 :
   Alpha helix      (Hh) :   188 is  49.21%
   3_10 helix       (Gg) :     0 is   0.00%
   Pi helix         (Ii) :     0 is   0.00%
   Beta bridge      (Bb) :     0 is   0.00%
   Extended strand  (Ee) :    31 is   8.12%
   Beta turn        (Tt) :     0 is   0.00%
   Bend region      (Ss) :     0 is   0.00%
   Random coil      (Cc) :   163 is  42.67%
   Ambiguous states (?)  :     0 is   0.00%
   Other states          :     0 is   0.00%
```

XP_013601844_L_Bo

```
         10        20        30        40        50        60        70
          |         |         |         |         |         |         |
MEFLLELLLTAVVALLFSFLVAKIVSVSMAGESDRSSDQIEKTEIGVGDGSATVEELCFGLKVDAPVVQS
cccchhhhhhhhhhhhhhhhhhhhhhhhcccccccchhhhhheeeecccccccchhhhhhccccccchhhh
ERKLRVVVDENVEHVDRFGNGADRVVDKVEEAARDVELVVLTTEANEFLAALSPGNVIAKEMIVRDEDEG
hhcceeeecccccchhhhcccccchhhhhhhhhhhhhhhhhhhhhhhhhhhhccccchhhhhhhhhhhhcc
REETSGEVGDERQELIESTAEAESTASVVQENMIAEEIINRGHEEELSSAEGVSSCVERGEVVVTESEEV
ccccccccchhhhhhhhhhhhhhchhhhhhhhhhhhhhhhhccchhhhhhccccceeccccccceeeeccccc
RVEESNSGEKSEDKMEFSIEEQVELSIEEDDDDDDWEGIEKSELEITFSAASNLLEQSGKGEEITAEAKM
eeeecccccchhhhhhhhhhhhhhhhhcccccccchhhhhhhhhhhhhhhhhhhhhhcccchhhhhhhhh
ELYGLHKIATEGSCREAQPMAIMLSARAKWNAWQRLGNMSQEEAMEQYLALVSKEIPGLLNTVGKMPETE
hhhhheeeccccccccccchhhhhhhhhhhhhhhhhcchhhhhhhhhhhhhhhhcccceecccccccccc
TSVDLGSLEDPTTLDTIGVATSKNEIVSGEDESSV
ccccccccccccceeeeeecccccceecccccceec

Sequence length :   385

GOR4 :
   Alpha helix     (Hh) :   207 is  53.77%
   3₁₀ helix       (Gg) :     0 is   0.00%
   Pi helix        (Ii) :     0 is   0.00%
   Beta bridge     (Bb) :     0 is   0.00%
   Extended strand (Ee) :    33 is   8.57%
   Beta turn       (Tt) :     0 is   0.00%
   Bend region     (Ss) :     0 is   0.00%
   Random coil     (Cc) :   145 is  37.66%
   Ambiguous states (?) :     0 is   0.00%
   Other states         :     0 is   0.00%
```

XP_009137819_L_Br

```
        10        20        30        40        50        60        70
         |         |         |         |         |         |         |
MEFLLELLLTAVVALLFSFLVAKIVSVSMAGENDGSSDQAGETEIGVGDGSATVEELCFGLKVDAPVVQS
cccchhhhhhhhhhhhhhhhhhhhhhhhhccccccccccccceeeeecccccchhhhhhccccccchhhh
ERKLRAVVDENVEHVDRFGSGADRVVDEVEEAARDVELVVPTTEANEFLAAVSPGNVVAKEMIVRGEEET
hhhhhhhhhcchhhhhhccccccchhhhhhhhhhhhhhhcccchhhhhhhhcccccchhhhhhhhccccc
GYEREELVVSTAEAESTASISPENVITEEIMNRGQEEGTEGRSDCVENVKREVVVTESEKVRVEESNSVE
chhhhhhhhhhhhhhcccccccccchhhhhhhhcccccccccccccccccceeeeeccccceeecccchh
KSEDKMELSIEEQVELSIEEEDLDDDWEGIERSELEIAFAAASNLLEESGKGEDIGAEAKMELYGLHKIA
hhhhhhhhhhhhhhhhhhhhcccccchhhhhhhhhhhhhhhhhhhhhhccccchhhhhhhhhhhhheeec
TEGSCREAQPMAVMLSARAKWNAWQRLGNMSQEEAMEQYLALVSKEIPDLVNTVGKMPETETSVDLPPNS
cccccccccchhhhhhhhhhhhhhhhhcchhhhhhhhhhhhhhhhcccceecccccccccceeccccccc
GSLEDPTTLDTIGVATSKNEIHVSGEDESSV
cccccccceeeeeecccccceeeecccceec
```

```
Sequence length :   381
```

```
GOR4 :
   Alpha helix      (Hh) :   193 is  50.66%
   3₁₀ helix        (Gg) :     0 is   0.00%
   Pi helix         (Ii) :     0 is   0.00%
   Beta bridge      (Bb) :     0 is   0.00%
   Extended strand  (Ee) :    32 is   8.40%
   Beta turn        (Tt) :     0 is   0.00%
   Bend region      (Ss) :     0 is   0.00%
   Random coil      (Cc) :   156 is  40.94%
   Ambiguous states (?)  :     0 is   0.00%
   Other states          :     0 is   0.00%
```

XP_016732243_L_Gh

```
        10        20        30        40        50        60        70
         |         |         |         |         |         |         |
MEIILELFLTAFIALVFSFLIAKIVSLATGGDAGGGCSGGDDDKIIMEQLEFAEKFKVSSFESEKKVDFV
cccchhhhhhhhhhhhhhhhhhhhhhhhccccccccccccccchhhhhhhhhhhhhhhhcccccceeeee
KESGDDNKVVVLESVDDDDNDDQGFKSETKVDELEGEESQEVVERSKLESFVQETDKKIDGFEAEVERIG
eccccccceeeeeeeccccccccccccchhhhhhhcccchhhhhhhhhhhhhhhhhhccccchhhhhhhhh
EEIEAENKTKFQPQEIRIEESQMKVLGEEEKEVKLANYEDEDDDWEGIEKSELEKVFGSASKFIEQEGDL
hhhhhhhhccccchhhhhhhhhhhhhhhhhhhhhhhcccccccchhhhhhhhhhhhhcccchhhhhcccc
GIGNDVQMELYGLHKVATEGPCHESQPLAFMVASRSKWNAWQKLGNMSPEAAMEQYVALVSDKVPGWTKY
ccccchhhhhhhhhhhcccccccccccceeeeeeecchhhhhccccccchhhhhhhhheeecccccceee
TSDGERKLESADQGVAGSVAPDIDSFPDKQAIFMHERNADSNTAPAGGDITESASLEKQAKDLGIGNDVQ
ccccchhhhhhccccccccccccccccchhhhhhhhhccccccccccccchhhhhhhhhhhhhccccchh
MELYGLHKVATEGPCHEPQPLAFMVASRSKCCYV
hhhhhhhhhcccccccccccceeeeeeceeeeec

Sequence length :   384

GOR4 :
   Alpha helix      (Hh) :   187 is  48.70%
   3_10  helix      (Gg) :     0 is   0.00%
   Pi helix         (Ii) :     0 is   0.00%
   Beta bridge      (Bb) :     0 is   0.00%
   Extended strand  (Ee) :    37 is   9.64%
   Beta turn        (Tt) :     0 is   0.00%
   Bend region      (Ss) :     0 is   0.00%
   Random coil      (Cc) :   160 is  41.67%
   Ambiguous states (?)  :     0 is   0.00%
   Other states          :     0 is   0.00%
```

XP_003550311_L_Gm

```
         10        20        30        40        50        60        70
          |         |         |         |         |         |         |
MELLMVVDDFFVTASLALLLTFIVLKLVEAMNDTHAAPKRHVVHLEPDHPVSRAKTKVGLISPVQEEIAT
ccccccccchhhhhhhhhhhhhhhhhhhhhhhhhccccccceeeeccccccccccccccecccchhhhhh
CAVGAKHGIEKKAAVKSVRPRGKRFTVNPAQSKVKVEPVPPAQSESTIGFVSPVQSAMCVETERKVEEAM
hhcccccchhhhhhhhcccccccceeecccccccccccccccccccceeeeeccccccchhhhhhhhhhh
VEPELVQLVKTEGTVGFISPIQADTCVGTEDKKEEATVEFDSHVVVESPVKSDTDIVVTEEIAEGNEGTQ
hchhhhhhhccccccceeeccccccccccccccchhhhhhccceeeeccccccccceeeehhhhcccccch
ELDDSAEKRIVESVEEINVEPSVVDSDGKENEDDYDDDWEGIERSELEKEFMAATEFVSGEENRLGGVGS
hhhhhhhhhhhhhhhccccccceeccccccccccccccchhhhhhhhhhhhhhhhhhhhcccccccccccc
NLRMELYGLYKVATEGLCREPQPMALKLAARAKWNAWQKLGNMNPEVAMEQYISLLSDKFPGWIKDTSAG
chhhhhhhheeeccccccccccchhhhhhhhhhhhhhhccccccchhhhhhhhheeecccccceeeccccc
MSEHETTRPEVSGSDASDLSTTLSHQQMIITERELEQESDSKDRSPLTETDLENNVNK
ccccccccccccccccccchhhhhhhhhhhhhhhhhhhcccccccccchhhhccceec

Sequence length :   408

GOR4 :
   Alpha helix      (Hh) :   161 is  39.46%
   3_10 helix       (Gg) :     0 is   0.00%
   Pi helix         (Ii) :     0 is   0.00%
   Beta bridge      (Bb) :     0 is   0.00%
   Extended strand  (Ee) :    37 is   9.07%
   Beta turn        (Tt) :     0 is   0.00%
   Bend region      (Ss) :     0 is   0.00%
   Random coil      (Cc) :   210 is  51.47%
   Ambiguous states (?)  :     0 is   0.00%
   Other states          :     0 is   0.00%
```

XP_022011286_L_Ha

```
        10        20        30        40        50        60        70
         |         |         |         |         |         |         |
MELVQELVFTISFSLIVSLLIAKLFSVGSSGNSSVSIRVEEKIEKEGNFDDPVKGCEEGGFGCLEGDLGS
ccccccceeecchhhhhhhhhhheeeccccccccchhhhhhhhhhccccccccccccccccceecccccc
GVVVDCGGGEGAGYVFDESPERNEDDNGDAGLVKSDEGNMVTFSGSLVEEDCVKCVEVETESISGVGVCY
eeeeeccccccceeeecccccccccccccceeeeccccceeeeecceeeecccccccccccccccceeee
VFDESHERTECKDDDDGSLVKSDEGNLVNFSGLLVQENCVKSVEVDTESGSGVGVRYVFDESPERTECKE
eecccccceeeccccccceeeecccccceeeecceeeeecccceeeecccccccceeeeeecccccceeccc
GGGNLVKSDEGNVVNFTGSLVQEDCVKSVEVERVFSAINDGEVKGEEEDEMFDDDWQGVETTELERRFGA
cccceeeccccceeeeeceecccccccccccceeeeeccccccccccccccccccccccchhhhhhhhhh
AVAFMDAKVNSGVVNLIDNEVKVELYGLHRVAIEGSCFEPQPMALKVSARANWNSWKRFENLGREDAMEQ
hhhhhhhccccceeeeeccchhhhhhccceeeeccccccccccceeeeeechhhhhhhhcccccchhhhh
YIALLSRHVPGWMGSHSYDKQ
hhhheeeccccceeeceeeec
```

Sequence length :   371

GOR4 :

Alpha helix	(Hh)	:	61	is	16.44%
3_{10} helix	(Gg)	:	0	is	0.00%
Pi helix	(Ii)	:	0	is	0.00%
Beta bridge	(Bb)	:	0	is	0.00%
Extended strand	(Ee)	:	104	is	28.03%
Beta turn	(Tt)	:	0	is	0.00%
Bend region	(Ss)	:	0	is	0.00%
Random coil	(Cc)	:	206	is	55.53%
Ambiguous states	(?)	:	0	is	0.00%
Other states		:	0	is	0.00%

XP_012068250_L_Jc

```
         10         20         30         40         50         60         70
          |          |          |          |          |          |          |
MELLQELFVTAFVAVVCSFLIAKIVSIAMAGGDSSNASQLSKSQNDDQKITGDDGDERVMEDLRYFEKLK
ccccchhhheeeeeeechhhhchhhhhhhccccccchhhhhhcccccceeeccccchhhhhhhhhhhhhh
VEGYTSEKRVEFVQEGAQKVDGFVGGSIEAAEVEKSVNRDEVIETECRELVTISVEEGLKEEDKSSEDML
hccccccchhhhhhhcccccccccccchhhhhhhhhhcchhhhhhhccchhhhhhhhhcchhhhhhhhhh
GKCEGEFQVNRHVGVELADGKGAIDEKQRGVDDNLLKENIEIESVGIESSVKKDAVEESEEIRVVGSETI
hhccccccccceeeeeeecccchhhhhhhchhhhhhhcceeeeeecchhhcccchhhhhhhheeeccccch
EKGEEKKIEIDSDDDDWEGIERSELDHVFAKAVNLVESADKDGGSRSIGSDVQMELYGLHKVATEGPCRE
hhhhhhheeeecccccchhhhchhhhhhhhhhhhhhhhccccccccceeccchhhhhhhheeecccccccc
QPPLPLKVAARAKWNAWQRLGNMNPEVAMEQYVALVSDKVPGWMEGKSADDDKPGSSAAANPGAVASDLS
ccccchhhhhhhhhhhhhhcccccchhhhhhhheeeecccccceeeccccccccccccccccccceeeccc
TTSSHHQNITEERNPEVMLGIEKNDYIGPNVEDKVKE
cccccccccccccchhhheeeccccccccccccceeec
```

```
Sequence length :   387
```

```
GOR4 :
   Alpha helix     (Hh) :   168 is  43.41%
   3_10  helix     (Gg) :     0 is   0.00%
   Pi helix        (Ii) :     0 is   0.00%
   Beta bridge     (Bb) :     0 is   0.00%
   Extended strand (Ee) :    51 is  13.18%
   Beta turn       (Tt) :     0 is   0.00%
   Bend region     (Ss) :     0 is   0.00%
   Random coil     (Cc) :   168 is  43.41%
   Ambiguous states (?)  :     0 is   0.00%
   Other states          :     0 is   0.00%
```

XP_022844505_L_Oe

```
        10         20         30         40         50         60         70
         |          |          |          |          |          |          |
MADWQQYIQSVIVGVIFSFLLAKLFSIIFAFRDENLRITRSATLEPESEPEELQTSSLELLEEKEPLIHE
ccccccceeeeecchhhhhhhhhhhhhhhhhhhhchhhhhhccccccccchhhhhhhhhhhhhhchhhhh
SEELRYGSSSVGDHSDSDDDWEGVESTELDEEFSAATAFVAATAADKAAQKVSNELQLQLYGLYKIATEG
hhhhhcccceecceeccccccccccccchhhhhhhhhhhhhhhhhhhhhhhhhhhhhhhhhhhheeecccc
PCSAPQPSALKLTARAKWQAWQKLGTMPPEEAMQKYIDIVTELYPTWATGAATKRKYKEASDPPNAGSKG
cccccccchhhhhhhhhhhhhhccccccchhhhhhhhheeeeecccccchhhhhhhhhcccccccccccc
PMGPVFSSFVHEEEPEIESKLDVIHAFAREGDEEKLLKCIESGIPVNLKG
cccceeccccccccchhhhhhhhhhhhhhhcchhhhhhhhcccccccceec

Sequence length :   260

GOR4 :
   Alpha helix      (Hh) :   141 is   54.23%
   3_10 helix        (Gg) :     0 is    0.00%
   Pi helix         (Ii) :     0 is    0.00%
   Beta bridge      (Bb) :     0 is    0.00%
   Extended strand  (Ee) :    21 is    8.08%
   Beta turn        (Tt) :     0 is    0.00%
   Bend region      (Ss) :     0 is    0.00%
   Random coil      (Cc) :    98 is   37.69%
   Ambiguous states (?)  :     0 is    0.00%
   Other states          :     0 is    0.00%
```

Os03g14000_L_Os

```
         10        20        30        40        50        60        70
          |         |         |         |         |         |         |
MELFYELLLTAAASLLVAFLLARLLASAATASDPRRRAPDHAAVIAEEEAVVVEEERIIEVDEVEVKSAR
cccchhhhhhhhhhhhhhhhhhhhhhhhhhcccccccchhhhhhhhhhhhhhhhhhhhhhhhhhhhhhhh
ARECVVSEGWVEVGRASSAEGKLECLPEEEEAPAKAARELVLDAVLEEREEEGQVGEERCDLAAAVAEVV
cceeeeeccceeeccccccccccccccchhhhhhhhhhhhhhhhhhhhhhhhcchhhhhhhhhhhhhhhhh
GVKPHELGVEAAPGEVSDVTLEEGKVQDVGVEQHDLVAEAAPREALDTGLEKQGVPIIEAVEIKRQDDLG
cccccccccccccccccceeeccccccceecccchhhhhhhhhhhhhhhhhhcccccchhhhhhhhhhhhhc
AEVAPSDVPEVEFEQQGVRIIEAIDVNQHHRVALAAPAEVVDAGLEERVQAIEAGSSGLTSETVPEEVLD
cccccccccchhhhhcchhhhhhhchhhhhhhhhhcchhhhhhhhhhhhhhhhcccccccccccchhhhh
ELSEKQEEQVIEEKEHQLAAATAPVAIPGVALAETEELKEEQSSEKAVNVHEEVQSKDEAKCKLHLVDQQ
hhhhhhhhhhhhhhhhhhhhhcccccccchhhhhhhhhhhhhhhhhhhhhhhhhhhhhhhhhhhhheeeccc
EGSASKVELVGRNTDNVEISHGSSSGDKMIAELTEEELTLQGVPADETQTDMEFGEWEGIERTEIEKRFG
ccccceeeeecccccceeeecccccchhhhhhhhhhhhhhcccccccccchhhhccccchhhhhhhhhhh
VAAAFASSDAGMAALSKLDSDVQLQLQGLLKVAIDGPCYDSTQPLTLRPSSRAKWAAWQKLGNMYPETAM
hhhhhhhhhhhhhhhhhhchhhhhhhhhheeeeeccccccccccccccccccchhhhhhhcccccccchhhh
ERYMNLLSEAIPGWMGDNISGTKEHEAGDDAVGSVLTMTSNTINQHDSQGNEDNTGMYEGHLTSSPNPEK
hhhhhheeeeeccccccccccccccccccccccceeeeccccccccccccccccccceeeeeecccccccce
EF
ec
```

Sequence length : 562

GOR4 :

Alpha helix	(Hh) :	303	is	53.91%
3_{10} helix	(Gg) :	0	is	0.00%
Pi helix	(Ii) :	0	is	0.00%
Beta bridge	(Bb) :	0	is	0.00%
Extended strand	(Ee) :	47	is	8.36%
Beta turn	(Tt) :	0	is	0.00%
Bend region	(Ss) :	0	is	0.00%
Random coil	(Cc) :	212	is	37.72%
Ambiguous states	(?) :	0	is	0.00%
Other states	:	0	is	0.00%

AFZ62129_L_Vf

```
         10        20        30        40        50        60        70
          |         |         |         |         |         |         |
MELLQELFVTAVFAVVCSFLIAKLVSMAMAGADSSHDSQFSKSQNVDQKITRDDDDLQYFEKLKVEGFKS
ccccchhhhhhhhhhhhhhhhhhhhhhhhhcccccccccccccccccceeccccchhhhhhhhhhccccc
EKRVKFTDEVAEMVDEFVDEPVDVEKSVNRDEPIETECRELRPKSIKGSLKEEEELENVLGKREAEFEEN
ccccchhhhhhhhhhhccccccccccccccccccchhhhcccccccccchhhhhhhhhhhhhhhhhhhhh
KQVGVKLAADKSFTEKSEEVIEEKQKGEIESIGIEFAAEHDVVEESEEIRIVDSEAKEKAEEKKIEIESD
hhhhhhhhccccchhhhhhhhhhhhhcchhhhhhhhhhhhcchhhhhhhhhhhhhhhhhhhhhhhheeecc
EDDWEGIERSELEQIFAKAAKFVESGDKDEGLTSVGSDVQMELYGLHKVATEGPCREQPPMALKVAARAK
ccchhhhhhhhhhhhhhhhhhhhhccccccceeecccchhhhhhhhheeecccccccccccchhhhhhhhh
WNAWQRLGNMNPEVAMEQYVALVSDKVPGWMEDKSTDNGKPGSTEAANHGALPSDLSTSSSHHPYITEER
hhhhhhcccccchhhhhhhheeeecccccceeecccccccccchhhhccccccccccccccccceeeccc
NPEVAPGTEKNDLTGGLILENRATE
cccccccccccccccchhhhcceec
```

```
Sequence length :   375
```

```
GOR4 :
   Alpha helix     (Hh) :   187 is  49.87%
   3₁₀ helix       (Gg) :     0 is   0.00%
   Pi helix        (Ii) :     0 is   0.00%
   Beta bridge     (Bb) :     0 is   0.00%
   Extended strand (Ee) :    23 is   6.13%
   Beta turn       (Tt) :     0 is   0.00%
   Bend region     (Ss) :     0 is   0.00%
   Random coil     (Cc) :   165 is  44.00%
   Ambiguous states (?)  :     0 is   0.00%
   Other states          :     0 is   0.00%
```

ONL95885_L_Zm

```
        10        20        30        40        50        60        70
         |         |         |         |         |         |         |
MELFYELLLTAAVTLLAALLLATLFAANDSHRRTDRAAAAIAEEVAEEERIIEVDEVMRSEGMAAVAPSE
cccchhhhhhhhhhhhhhhhhhhhhhhcchhhhhhhhhhhhhhhhhhhhhhhhhhhhhhhhhhhhhhhhc
ADGWVEAEKAPAVVVAEVKEPESLPEEEGVPVKAVREVRLAAEVEEGEDGGGGVKRPDLTSATVVAAVGA
cchhhhhhccchhhhhhhcccccccccccccchhhhhhhhhhhhhhcccccccccccccccchhhhhhhhh
EASQLVSGAEVVPKEVSGAAGLEERTVQDVVVNQYDLGAEVALVPVEVLEAGPDKQGVEVVGVAQVPPLE
hhhhhhccceecccccccccccccccccceeeeccccchhhhhcchhhhcccccccceeeeeecccccccch
TEAAEVKQHHLVAEAAPAEDVLDVGLVDKSVRAIEVRPNELDSETVLEEILDAVLEEEEQVEHELPAGAA
hhhhhhhhhhhhhhhcchhhhhhhcccccceeeeeeccccccchhhhhhhhhhhhhhhhhhhhhcccccc
PQPVLDVPLAGKGELKHHQPVEEAAEVHEDVQCKQEAECEAQTVDQQQELVPEEESVAGKNDDVNVIHEC
ccccccccccccccccccchhhhhhhhhhhhhhhhhhcccccccchhhhcchhhhhhcccccceeeeeec
SFSDEVVTELPVGEVTSQGLPKDDPEADMEFEEWEGIERSEVEKRFGAAAAFAASGAGAAALSKLDSDVQ
ccccceeeecccccEEcccccccccchhhhhhhhhhhhhhhhhhhhhhhhhhhhhhhhhhhhhhhhchhhh
LQLQGLLKVAIDGPCYDSAQPLTLRPSSRAKWSAWQKLGNMNPEIAMEKYMNLLSETIPGWMMNETLDTE
hhhhhheeeeecccccccccccccccccchhhhhhhhccccccchhhhhhhhheeeeeeccccceeeecceecccc
NTGSLPAETILTTMASTSDQRSNQGNEDNSSIGEGHPITSPNPEKGM
cccccceeeeeeeeecccchhhcccccccccccccccceeccccceec
```

Sequence length : 537

```
GOR4 :
   Alpha helix      (Hh) :   255 is  47.49%
   3_10  helix      (Gg) :     0 is   0.00%
   Pi helix         (Ii) :     0 is   0.00%
   Beta bridge      (Bb) :     0 is   0.00%
   Extended strand  (Ee) :    58 is  10.80%
   Beta turn        (Tt) :     0 is   0.00%
   Bend region      (Ss) :     0 is   0.00%
   Random coil      (Cc) :   224 is  41.71%
   Ambiguous states (?)  :     0 is   0.00%
   Other states          :     0 is   0.00%
```

Kelch motif ACBP

AT3G05420_K_At

```
        10        20        30        40        50        60        70
         |         |         |         |         |         |         |
MAMPRATSGPAYPERFYAAASYVGLDGSDSSAKNVISKFPDDTALLLYALYQQATVGPCNTPKPSAWRPV
ccccccccccccchhhhhhheeeecccccccceeeeeccccchhhhhhhhhcccccccccccccccccce
eeceeeeeecccccchhhhhhhhhheeecccccceeeccccccccchhhhhhccccccceeccccccccc
eeeeecchhhhccccceeecccceeeeecccccccccccccchhhhhhchhhhhhhheeeccccccccccc
LHVLDLKSWTWSRVETKVATESQETSTPTLLAPCAGHSLIAWDNKLLSIGGHTKDPSESMQVKVFDPHTI
eeeecccccceeeeeeecccccccccccceeecccccceeeeceeeeeeecccccccccccceeeeeccee
TWSMLKTYGKPPVSRGGQSVTMVGKTLVIFGGQDAKRSLLNDLHILDLDTMTWDEIDAVGVSPSPRSDHA
eeeeeecccccccccccceeeeeeceeeeeecccchhhhhhhhhhhhcccccccceeccccccccccchhh
AAVHAERFLLIFGGGSHATCFDDLHVLDLQTMEWSRPAQQGDAPTPRAGHAGVTIGENWFIVGGGDNKSG
hhhhhhhheeeecccccee ccchhhhhhhhhhhhhccccccccccccccccceeeccceeeecccccccc
ASESVVLNMSTLAWSVVASVQGRVPLASEGLSLVVSSYNGEDVLVAFGGYNGRYNNEINLLKPSHKSTLQ
cceeeeeeecccceeeeecccccccccccccceeeeecccccceeeeeccccccccccceeecccccccchhh
TKTLEAPLPGSLSAVNNATTRDIESEVEVSQEGRVREIVMDNVNPGSKVEGNSERIIATIKSEKEELEAS
hhcccccccccccccccccccchhhhhhhhhhccceeeeeeecccccccccccchhhhhhhhhhhhhhhhh
LNKERMQTLQLRQELGEAELRNTDLYKELQSVRGQLAAEQSRCFKLEVDVAELRQKLQTLETLQKELELL
hhhhhhhhhhhhhhhhhhhhhhhhhhhhhhhhhhhhhhhhhhhhhhhhhhhhhhhhhhhhhhhhhhhhhh
QRQKAASEQAAMNAKRQGSGGVWGWLAGSPQEKDDDSP
hhhhhhhhhhhhhhhhccccceeeeeeccccccccceee
```

```
Sequence length :   668
```

```
GOR4 :
   Alpha helix      (Hh) :   203 is  30.39%
   3_10 helix       (Gg) :     0 is   0.00%
   Pi helix         (Ii) :     0 is   0.00%
   Beta bridge      (Bb) :     0 is   0.00%
   Extended strand  (Ee) :   147 is  22.01%
   Beta turn        (Tt) :     0 is   0.00%
   Bend region      (Ss) :     0 is   0.00%
   Random coil      (Cc) :   318 is  47.60%
   Ambiguous states (?)  :     0 is   0.00%
   Other states          :     0 is   0.00%
```

AIS76197_K_Bn

```
        10        20        30        40        50        60        70
         |         |         |         |         |         |         |
MAKQSATLAYPDRFYAAASYLGLDGSAPSSVKQLSSKFSNDTALLLYALHQQATVGPCNTPKPSAWNPAE
cccccccccccchhhhhhhhcccccccccchhhhccccccchhhhhhhhhcccccccccccccccccccc
QSKWKSWQGLGTMPSIEAKRHFVKILEEGDSSWYPNPPNSVPDPAIDVQISSTKAEPSVENGGSFGETMT
cceeeeeecccccchhhhhhhhhheeecccccccccccccccccccceeeecccccccccccccccceeee
TATEDGRLMETQDKDVVLENPNTISVYNQWTAPLTSGHPPKARYEHGAAVIQDKMYMYGGNHNGRYLGDL
eeeechhhhhhcccceeecccceeeeeccceecccccccccchhhhceeeeeeeeeeeecccccccccce
HVLDLKNWTWSRVETKVVTESQETSSPATLTHCAGHSLIPWDNKLLSIGGHAKDPSESILVKVFDLHTCT
eeecccccceeeeeeeeeeccccccccceeecccccccccccceeeeecccccccccceeeeeeeeeccce
WSILKTDGKPPISRGGQSVTLVGKKLVIFGGQNVNKSLLNDLHLLDLDTMTWDEIDAVGSPPSPRSDHAA
eeeecccccccccccccceeeeeceeeeeecccccchhhhhhhhhhchhhhchhhhccccccccccchhhh
AVHAERYLLIFGGGSHTNCFSDLHVLDLQTMEWSRHAQQGEAPTPRAGHAGVTIGENWFIVGGGDNKSGA
hhhhhhheeeeccccccccccchhhhhhhhhhhhhhccccccccccccccceeeccceeeeccccccccc
CETVVLNMSTLAWSVLTTVQGGAPLASEGLSLVVSSYNGEDVIVAFGGYNGRYNNEVNVLKPSHKSSLKS
ceeeeecccccceeeeeecccccccccccceeeeecccccceeeeecccccccccceeeecccccchhhh
KIMEASPVRDSVSAVNNATTRDIEFEIGVSQESKVREIVMDNVNSGSKVEGKSERIITSLRSEKEELEAS
hhhhccccccceeeecccccccchhhhhcchhhhhhheeeeecccccccccccchhhhhhhhhhhhhhhhh
LSKEKIQTLQLKEELTETETRSTELYKELHSVRSQLAAEQSRCFKLEVEVAEVRQKLQTMETLEKELELL
hhhhhhhhhhhhhhhhhhhhhhhhhhhhhhhhhhhhhhhhhhhhhhhhhhhhhhhhhhhhhhhhhhhhhh
HRQRAVASEQAAVNMNGKPQSSGGVWGWLAGTPPPKT
hhhhhhhhhhhhhhccccceeccceeeeeeccccceee

Sequence length :   667

GOR4 :
   Alpha helix      (Hh) :   205 is  30.73%
   3_10 helix       (Gg) :     0 is   0.00%
   Pi helix         (Ii) :     0 is   0.00%
   Beta bridge      (Bb) :     0 is   0.00%
   Extended strand  (Ee) :   144 is  21.59%
   Beta turn        (Tt) :     0 is   0.00%
   Bend region      (Ss) :     0 is   0.00%
   Random coil      (Cc) :   318 is  47.68%
   Ambiguous states (?)  :     0 is   0.00%
   Other states          :     0 is   0.00%
```

XP_013616076_K_Bo

```
         10        20        30        40        50        60        70
          |         |         |         |         |         |         |
MANTAKQSATLAYPDRFYAAASYLGLDGSAPSSVKQLSSKFSNDAVLLLYALHQQATVGPCNTPKPSAWN
ccccccccccccccchhhhhhhhcccccccccchhhhcccccchhhhhhhhhhccccccccccccccccc
PAEQSKWKSWQGLGTMPSIEAMSHFVKILEEGDPSWYPNPPNSVPDPAIDVQISQSTKAEPSIENGGSFG
ccccceeeeeeccccccchhhhhhhheeeecccccccccccccccccccceeeeecccccccccccccccc
ETMTTATEDGRLMETQDKDVVLENPNTISVYNQWTAPLTSGHPPKARYEHGAAVIQDKMYMYGGNHNGRY
eeeeeeeechhhhhhcccceeecccceeeeeccceeccccccccccchhhhceeeeeeeeeeeeccccccc
LGDLHVLDLKNWTWSRVETKVVTESQETSSPATLTHCAGHSLIPWDNKLLSIGGHAKDPSESILVKVFDL
ccceeeecccccceeeeeeeeeecccccccccceeecccccccccccceeeeeccccccccceeeeeeeee
HTCTWSILKTDGKPPISRGGQSVTLVGKKLVIFGGQDVNKSLLNYLHLLDLDTMTWDEIDAVGSPPSPRS
ccceeeeeccccccccccccceeeeecceeeeecccccchhhhhhhhhhcccccchhhhccccccccccc
DHAAAVHAERYLLIFGGGSHTNCFSDLHVLDLQTMEWSRHAQQGEAPTPRAGHAGVTIGENWFIVGGGDN
hhhhhhhhhhheeeeccccccccccchhhhhhhhhhhhhhccccccccccccccceeeccceeeeccccc
KSGACETVVLNMSTLAWSVLTTVQGGAPLASEGSSLVVSSYNGEDVIVAFGGYNGRYNNEVNVLKPSHKS
ccccceeeeecccccceeeeeecccccccccccceeeeecccccceeeeecccccccccceeeecccccc
SLKSKIMEASPVRDSVSAVNNATTRDIESEIGVSQESKVREIVMDNVNSGSKVEGKSERIITSLRSEKEE
hhhhhhhhccccccceeeecccccccchhhhccchhhhhhhheeeeecccccccccccchhhhhhhhhhhhh
LEASLSKEKIQTLQLKEELTETETRSTELYKELHSVRSQLAAEQSRCFKLEVEVAEVRQKLQTMETLEKE
hhhhhhhhhhhhhhhhhhhhhhhhhhhhhhhhhhhhhhhhhhhhhhhhhhhhhhhhhhhhhhhhhhhhhh
LELLHRQRAVASEQAAVNMNGKPQSSGGVWGWLAGTPPPKT
hhhhhhhhhhhhhhhhhhcccccceeccceeeeeeccccccee
```

Sequence length : 671

GOR4 :

Alpha helix	(Hh) :	199	is	29.66%
3_{10} helix	(Gg) :	0	is	0.00%
Pi helix	(Ii) :	0	is	0.00%
Beta bridge	(Bb) :	0	is	0.00%
Extended strand	(Ee) :	146	is	21.76%
Beta turn	(Tt) :	0	is	0.00%
Bend region	(Ss) :	0	is	0.00%
Random coil	(Cc) :	326	is	48.58%
Ambiguous states (?)	:	0	is	0.00%
Other states	:	0	is	0.00%

XP_009129871_K_Br

```
         10        20        30        40        50        60        70
          |         |         |         |         |         |         |
MAKASATLAYPDRFYAAVSYLGLDGSVPSSVKQLSSKFSNDTALLLHALHKQATVGPCNVPKPSAWNPAE
cccccccccccceeeeeeeeeccccccccchhhhcccccchhhhhhhhhhcccccccccccccccccccc
QSNWRSWQGLGAMPSIEAMRHFVKILEEGDPSWYPNPTNSAPEPAIDVQISQSTKAEPSVENGGSFGETM
ccchhhhhhccccchhhhhhhhheeeecccccccccccccccccceeeeeccccccccccccccccceee
TTATEDGRLMETQDKDVVLENPNTISVYNQWTAPFTLGQPPKARYEHGAAVIQDKMYMYGGNHNGRYLGD
eeeeechhhhhhcccceeecccceeeeeccccccccccccchhhhhceeeeeeeeeeeecccccccccc
LHVLDLKNWTWSRVETKVVTESQETSSPATLTHCAGHSLIPWDNKLLSIGGHAKDPSESILVKVFDLHTC
eeeecccccceeeeeeeeeeccccccccceeeccccccccccccceeeeecccccccccceeeeeeeeeccc
TWSILKTDGKPPISRGGQSVTLVGKKLVIFGGQDVNKSLLNDLHLLDLDTMTWDEIDAVGSPPSPRSDHA
eeeeecccccccccccccceeeeecceeeeecccccchhhhhhhhhhchhhhchhhhccccccccccchhh
AAVHAERHLLIFGGGSHTNCFSDLHVLDLQTMEWSRHAQQGEAPTPRAGHAGVTIGENWFIVGGGDNKSG
hhhhhhhhheeeccccccccccchhhhhhhhhhhhhhccccccccccccccceeeccceeeecccccccc
ACETVVLNMSTLAWSVLTSVQGGVPLASEGLSLVVSSYNGEDVIVAFGGYNGRYNNEVNVLKPSHKSSLK
cceeeeecccccceeeeeccccccccccccceeeeecccccceeeeecccccccccceeeecccccchhh
SKIMEASPVRDSVSAVNNATTRDIESEIGVSQESKVREIVMDNVNSGSKVEGKSERIITSLRSEKEELEA
hhhhhccccccceeeecccccccchhhhccchhhhhhheeeeecccccccccccchhhhhhhhhhhhhhhh
SLSKEKIQTLQLKEELTETETRSAELYKELHSVRSQLAAEQSRCFKLEVEVAEVRQKLQTMETLEKELEL
hhhhhhhhhhhhhhhhhhhhhhhhhhhhhhhhhhhhhhhhhhhhhhhhhhhhhhhhhhhhhhhhhhhhhh
LHRQRAVASEQAAVNMNGKRQSSGGVWGWLAGTPPPKT
hhhhhhhhhhhhhhhhccceeecceeeeeeeccccccee
```

```
Sequence length :   668
```

```
GOR4 :
   Alpha helix      (Hh) :   205 is  30.69%
   3_10  helix      (Gg) :     0 is   0.00%
   Pi helix         (Ii) :     0 is   0.00%
   Beta bridge      (Bb) :     0 is   0.00%
   Extended strand  (Ee) :   147 is  22.01%
   Beta turn        (Tt) :     0 is   0.00%
   Bend region      (Ss) :     0 is   0.00%
   Random coil      (Cc) :   316 is  47.31%
   Ambiguous states (?)  :     0 is   0.00%
   Other states          :     0 is   0.00%
```

XP_016667870_K_Gh

```
         10        20        30        40        50        60        70
          |         |         |         |         |         |         |
MARASSGLAYPERFYAAAAYVGFDGSPNSNDKDIGSRFSTDVALILYALYQQATVGPCNVPKPSLWNPVE
cccccccccchhhhhhhhheeecccccccccccccccccchhhhhhhhhhccccccccccccccccccee
QGKWKSWKQLGNMVSAEAMRLFVKILEEEEPGWYSRASNSVLEPVTDVQMNVGHVPLLQHNAIVEPIIEN
ccceeeeeeccchhhhhhhhhhhhhhccccccceeecccccccceeccccccccccccccccccchhhhc
RNSFAETKTISAENGGLIETQDIDVVSEGLGSVVVYDQWTSLPITGQRPKGRYEHAAVVEDKMYIYGGN
ccccccchhhhhccccceeeccceeeecccceeeeecccccccccccccccchhhhhhhhhhhheeeecc
HNGRYLSDLHVLDLRSCTWSKIEAKVESKSVETPSPINITPSAGHSLIPWENKLLSIAGHTKDPSETFQV
cccccccceeeeeecccchhhhhhhhcccccccccccceecccccccchhhhheeeeccccccccchhhh
KAFDLQIGTWSMLNTYGKPPVSRGGQSVTLVGTTLVIFGGEDAKQTLLNDLHILDLETLTWDEIDAVGVP
hheeeeecceeeecccccccccccccceeeeeceeeeeecccchhhhhhhhhhhhcccccccccccccccc
PSPRSGHAAVVHAERYLLIFGGGSHATCFNDLHVLDLQTMEWSRPTQQGEIPVPRAGHAGVTVGENWFIV
ccccccchhhhhhhhheeeecccccceecccceeeechhhhccccccccccccccccccccceeeccceeee
GGGDNKSGASETVVLNMSTLAWSVVTSVEGRVPLASEGLSLVLGSLNGEDILISFGGYNGHYNSEVNVLK
ecccccccccceeeeeeecccceeeeeccccccccccccceeeecccccccceeeeeccccccccccceeeec
PSHKSTLQSKIMEGPLPDSVSAVHNATNPTRDLESEFEVGQEGKIRETAIDNFEPMKSKGGETSEHILAM
cccchhhhhhhcccccccceeeecccccccccchhhhhhhcccccchhhhhhcccccccccccchhhhhhh
LKAEKEELESSFNNEKLLSLQLKQELADAEARNTDLYKELQSVRGQLASEQSRCFKLEVDVAELRQKLQT
hhhhhhhhhhhhchhhhhhhhhhhhhhhhhhhhhhhhhhhhhhhhhhhhhhhhhhhhhhhhhhhhhhhhh
METLQRELELLQRQKATSEQVAINAKQRQGSGGVWGWLAGSTPQNADDA
hhhhhhhhhhhhhhhhhhhhhhhhhhhccccccceeeeecccccccceeec
```

```
Sequence length :   679
```

```
GOR4 :
   Alpha helix     (Hh) :   221 is  32.55%
   3₁₀ helix       (Gg) :     0 is   0.00%
   Pi helix        (Ii) :     0 is   0.00%
   Beta bridge     (Bb) :     0 is   0.00%
   Extended strand (Ee) :   119 is  17.53%
   Beta turn       (Tt) :     0 is   0.00%
   Bend region     (Ss) :     0 is   0.00%
   Random coil     (Cc) :   339 is  49.93%
   Ambiguous states (?)  :     0 is   0.00%
   Other states          :     0 is   0.00%
```

XP_006606527_K_Gm

```
         10        20        30        40        50        60        70
          |         |         |         |         |         |         |
MARASSGLQYPERFYAAASYVGFDGSTKSLTSKFSNSTALLLYSLYQQASIGPCNVPEPSSWKLVEHSKW
ccccccccccchhhhhhceeecccccccceeecccccchhhhhhhhhccccccccccccccccceeeccee
ASWNQLGNMSSTEAMRLFVKILEEEDPGWYSRASNSVLDPVVDVQMNHNSKVEPVIENANAYPEIKTIST
eeeeecccccchhhhhhhhheeeccccccceeecccccccceeeeeeccccccccchhhhccccccceeeee
ENGSHVGTQDKDVVIEGFGSVGVYDQWVAPPVSGQRPKARYEHGAAVVQDKLYIYGGNHNGRYLNDLHVL
cccccccccccceeeecccceeeeeceeecccccccccchhhhhhhhhhhhheeeeccccccccccccceee
DLRSWTWSKIEAEVVESTNSSSITFPCAGHSLIPWENKLLSIAGHTKDPNESIQVKVFDLPNATWTTLKT
eeeccchhhhhhheeeccccccceeeccccccccchhhheeeeccccccccccceeeeeecccccceeecc
YGKPPVSRGGQSVTFVGTSLVIFGGEDAKRTLLNDLHILDLETMTWDEIDAVGVPPSPRSDHAAAVHVER
ccccccccccceeeeecceeeeecccchhhhhhhhhhhhhhhhcchhhhcccccccccccchhhhhhhhh
YLLIFGGGSHATCYNDLHVLDMQTMEWSRPTQLGEIPTPRAGHAGVTVGENWFIVGGGDNKSGVSETVVL
eeeeeccccccceeeceeceeechhhhcccccccccccccccccccceeeccceeeeecccccccccceeeee
NMATLTWSVVTSVQGRVPVASEGSSLVVSSYDGEDILVSFGGYNGHYNNEVYVLKPSHKSTLQSKLIENP
eeceeeeeeeeecccccccccccceeeeecccccceeeeecccccccccceeeecccccchhhhhccccc
IPYSVSGAHNAANATRDLDSEAGHKGIIKELVMDSVDSIVCIIKSKGDVITVLKVEKEDLESSLYKEKLQ
ccccccccchhhhhhhhhhhhhcchhhhhhhhhcccccceeeeeecccceeeeehhhhhchhhhhhhhhhhh
TLQLKQELSETKTRNSDLCKELQSIRGQLASEQSRCFKLEVEVAELSQKLQTIGTLQKELELLQRQKAAS
hhhhhhhhhhhhchhhhhhhhhhhhcchhhhhhhhhhhhhhhhhhhhhhhhhhhhhhhhhhhhhhhhhhhh
ELAALNAKQKQGSGGVWGWLAGAPPPTQKEDDG
hhhhhhhhhcccccceeeeecccccccceeeec

Sequence length :   663

GOR4 :
   Alpha helix      (Hh) :   203 is  30.62%
   3_10  helix      (Gg) :     0 is   0.00%
   Pi helix         (Ii) :     0 is   0.00%
   Beta bridge      (Bb) :     0 is   0.00%
   Extended strand  (Ee) :   153 is  23.08%
   Beta turn        (Tt) :     0 is   0.00%
   Bend region      (Ss) :     0 is   0.00%
   Random coil      (Cc) :   307 is  46.30%
   Ambiguous states (?)  :     0 is   0.00%
   Other states          :     0 is   0.00%
```

XP_021993755_K_Ha

```
         10        20        30        40        50        60        70
          |         |         |         |         |         |         |
MAMARASSGLAYPDRFFAAAAYAGFGGSPNSSSKGVTSKFSNDVALLLYALYQQATVGPCTLPKPRGWSP
cccccccccccccchhhhhhhhhcccccccccccceeeeccchhhhhhhhhhcccccccccccccccccc
VEQSKWTSWNGLGNMASIEAMRLFVKILEEEDPGWYSRASNFISDPVVDVEMNHNLKVELATKNEITLPE
eecceeeeecccccchhhhhhhhhhhheeeccccccccccccccccchhhhhhhchhhhhhhccccccccc
MKTIPTENGNSVDKDVMVEGVGSVSVYDQWVAPPISGPRPKPRYEHAAAVVDDKMYIFGGNHNGRYLNDL
cceecccccccccccceeeeceeeeeeecceecccccccccccchhhhhhhhhhhheeeeccccccccccc
QTLDLRNWTWSTVEVKANSEDPVRVIPCAGHSLIPWEGNKLISIAGHSKDASEVVNVKAFDLQTYTWSTM
ceeeecccceeeeeeccccccceeeecccccceecccccccceeeeecccccchhhhhhhccceeeceeeee
KTYGKPPVSRGGQSVTLVGTNLVIFGGQDGNRTLLNDLHILDLETMTWDEMDTIGVSPAPRSDHAAAVHA
eeccccccccccceeeeecceeeeeccccccchhhhhhchhhhhhhcccccccceecccccchhhhhhhh
ERYLLIFGGGTHATCFNDLHVLDLKTMEWSRPSQQGEIPSPRAGHAGVTVGESWFIVGGGDNKSGVSETV
hhheeeecccccceeeccceeeechhhhcccccccccccccccccccceeecceeeeecccccccccceeee
VLNMSTLSWSVVTTVQGRVPLASEGLSLVLSSYNGEDVLVSFGGYNGKYNNEVNLLKPSHKSTLQATKSG
eeeecccccceeeeecccccccccccceeeeecccccceeeeecccccccccceeecccccchhhhhcccc
TPALGSGSGVQNATNGTRDVDVEFEAGQEAKVREISMDNNEPQIIEINEVSERSIAVLKAEKEELESALN
cceecccccceecccccccchhhhhhhhhhhhhhhhhcccccccceeeehhhhhhhhhhhhhhhhhhhhhhh
NEKSQSLQLKQELIEAESKNADLYKELQSVRGQLAAEQSRCFKLEVDVAELRQKVQTMDSLQKEVEILQR
hhhhhhhhhhhhhhhhhhhhhhhhhhhhhhhhhhhhhhhhhhhhhhhhhhhhhhhhhhhhhhhhhhhhhh
QKAASEEALAKQKQTSGGVWGWLAGAPPTHDT
hhhhhhhhhhhhhccccceeeeeecccceeec
```

```
Sequence length :   662
```

```
GOR4 :
   Alpha helix      (Hh) :   220 is  33.23%
   3_10 helix       (Gg) :     0 is   0.00%
   Pi helix         (Ii) :     0 is   0.00%
   Beta bridge      (Bb) :     0 is   0.00%
   Extended strand  (Ee) :   138 is  20.85%
   Beta turn        (Tt) :     0 is   0.00%
   Bend region      (Ss) :     0 is   0.00%
   Random coil      (Cc) :   304 is  45.92%
   Ambiguous states (?)  :     0 is   0.00%
   Other states          :     0 is   0.00%
```

XP_012089029_K_Jc

```
         10        20        30        40        50        60        70
          |         |         |         |         |         |         |
MAMARASSGLAYPERFYAAAAYAGFDGSPNSTNIVSSKFQNDTALLLYALYQQATAGPCNTPKPSTWNAV
cccccccccccchhhhhhhhhhccccccccccceeeccccchhhhhhhhhhhhcccccccccccccchhh
EQSKWKSWHGLANMASTEAMRLFVKILEEEDPGWYSRASNFVSEPVVDVQMNHNSKVEAVVENGNSFPET
hhhhhhhhhceechhhhhhhhhhhhheeecccccceeecccccccceeeeeeccccceeeeeecccccccc
KTTSSENGTVIETQDKDVVSEGLGSVVVYDQWIAPPISGQRPKARYEHDAAIIQDKMYIYGGNHNGRYLN
eeeccccceeeeecccceeeccceeeeeecceecccccccccchhhhhhhhhhhhhheeecccccccccc
DLHVLDLRSWAWSKVDAKIEEESDGSKTPATITPCAGHSLIPWENKLLTIAGHTKDPSESIQVKVFDPQT
chhhhhhhhhhhhhhchhhhhhccccccceeeecccccccccchhhheeeecccccccccceeeeeecccc
RCWSTLKTYGKAPVSRGGQSVSLVGTSLVIFGGQDAKRSLLNDLHILDLETMTWDEIDAIGVPPSPRSDH
cceeeecccccccccccccceeeeecceeeeecccchhhhhhhhhhhhhhhhhchhhhccccccccccchh
AAAVHAERYLLIFGGGSHATCFNDLHVLDLQAMEWTRPTQQGEIPSPRAGHAGVTVGENWFIVGGGDNKS
hhhhhhhhheeeecccccceeccchhhhhhhhhhhhcccccccccccccccccceeeccceeeeecccccc
GVSETVVLNMSTLVWSIVTSVEGRVPLASEGLSLVVSSYSGEDILVSFGGYNGRYSNDVNVLKPSHKSTL
cceeeeeeeccceeeeeeeccccccccccccceeeeecccccceeeeecccccccccccceecccccccc
QSKIVETPVPDSVSAVPNTTNPTRDLESESGQESKIREIVMDNADPEPMKTKEEVSSEHILATLKAEKEE
ccceeeccccccceeecccccccccccccccchhhhhheeeeccccccccccccccchhhhhhhhhhhhhh
LESSLSKEKLQTHQLKQELTEAETRNTDLYKELQSVRGQLAAEQSRCFKLEVDVAELRQKLQTMEALQKE
hhhhhhhhhhhhhhhhhhhhhhhhhhhhhhhhhhhhhhhhhhhhhhhhhhhhhhhhhhhhhhhhhhhhhh
LELLQRQKAASEQAALNAKQREGSGGVWGWLAGTPGSQRKDED
hhhhhhhhhhhhhhhhhhhhhhcccceeeeeeccccccceeec

Sequence length :   673

GOR4 :
   Alpha helix      (Hh) :   240 is  35.66%
   3₁₀ helix        (Gg) :     0 is   0.00%
   Pi helix         (Ii) :     0 is   0.00%
   Beta bridge      (Bb) :     0 is   0.00%
   Extended strand  (Ee) :   132 is  19.61%
   Beta turn        (Tt) :     0 is   0.00%
   Bend region      (Ss) :     0 is   0.00%
   Random coil      (Cc) :   301 is  44.73%
   Ambiguous states (?)  :     0 is   0.00%
   Other states          :     0 is   0.00%
```

XP_022895969_K_Oe

```
        10        20        30        40        50        60        70
         |         |         |         |         |         |         |
MARVSSGLAYPDRFYAAADYAGFGGSPNSSAKGVSSKFSNDAALLLYALYQQATVGPCNIPKPRGWSPVE
cccccccccccchhhhhhhhcccccccccccceeeccccchhhhhhhhhhccccccccccccccccccee
QSKWTSWSGLGNMASTEAMRLFVKILEEEDPGWYSRASHFVSEPMVDVEMHHNPKVEPVAENGHTKSTLA
eceeeeeecccchhhhhhhhhhhheeeccccccccеeccеeecccеeeeeccccccеeeeccccccchhh
ENDYLSETQDKDVLSEGLGVVNIYDQWVAPPVSGTLPKARYEHGAAVIDDKMYVFGGNHNGRYLNDLQVL
hcccccccccccccccceeeeeeccecccccccccccchhhhhchhhhceeeeeeecccccccccеeeee
DLRNWTWAKVNIQLGNEGPITSCAGHSLIPWGGNKLLSVAGHAKDPPETLQDNSQCLAFIITVKEFDLQT
cccccceeeeeeccccccccеeeccceecccccceeeeecccccccccccccccеeeeeeeeccccceе
CTWSTLKTCGKPPVSRGGQSVTLVGSILVIFGGQDAKRSHLNDLHILDLESMTWDEMDTLGVPPSPRSDH
ceeeeeccccccccccccceeeeeceeeeeeccccchhhhhhhhhhhhhhhhchhhhccccccccccchh
AAAVHAERYLLIFGGGSHATCFNDLHVFDLQTMEWSRPIQQGEIPSPRAGHASVTIGENWFIVGGGDNKS
hhhhhhhhheeeecccccеeccccccеechhhhcccceeccccccccccccеeeeeccceeeecccccсc
GVSETVVLNMSTLVWSVVTSVQRGAPLASEGSSLVLSSFSGEDILVSFGGYNGRYNNEVNILKPSHKSTL
cceeeeeeecceeeeeeeeeccccccccccccеeeeeccccccеeeeeccccccccccccеcccccchhh
QSKLMGTSVPDSIAAVQNATNATRDVESELETDQEGKIREIIMDNADSEPMIKKVGETHDHLLTTLKAEK
hhhcccccccchhhhhhhhcccchhhhhhhhhhhhchhhhhhhcccccchhhhhcccchhhhhhhhhhhh
EELELSLNKEKMQTLDLKQELMEAETRNTDLYKELQSVRGQLAAEQSRCFKLEVDVAEFRQKLQMLDTLQ
hhhhhhhhhhhhhhhhhhhhhhhhhhhhhhhhhhhhhhhhhhhhhhhhhhhhhhhhhhhhhhhhhhhhhh
KELELLRRQKAASEQAALNAKQRQNGGGGGVWGWLAGTPPTQNVNDS
hhhhhhhhhhhhhhhhhhhhhhhcccccceeeeeecccccccеeeec
```

```
Sequence length :   677
```

```
GOR4 :
   Alpha helix      (Hh) :   221 is  32.64%
   3_10 helix       (Gg) :     0 is   0.00%
   Pi helix         (Ii) :     0 is   0.00%
   Beta bridge      (Bb) :     0 is   0.00%
   Extended strand  (Ee) :   146 is  21.57%
   Beta turn        (Tt) :     0 is   0.00%
   Bend region      (Ss) :     0 is   0.00%
   Random coil      (Cc) :   310 is  45.79%
   Ambiguous states (?)  :     0 is   0.00%
   Other states          :     0 is   0.00%
```

XP_015629756_K_Os

```
        10        20        30        40        50        60        70
         |         |         |         |         |         |         |
MASSGLAYPDRFYAAAAYAGFGAGGATSSSAISRFQNDVALLLYGLYQQATVGPCNVPKPRAWNPVEQSK
cccccccccchhhhhhhhhcccccccccchhhhhhhhhhhhhhhhhhcccccccccccccccccceecce
WTSWHGLGSMPSAEAMRLFVKILEEEDPGWYSRVPEFNPEPVVDIEMHKPKEDPKVILASTNGTSVPEPK
eeeeecccccchhhhhhhhhheeecccccceeecccccccchhhhhhcccccccceeeeeecccccccccc
TISENGSSVETQDKVVILEGLSAVSVHEEWTPLSVNGQRPKPRYEHGATVVQDKMYIFGGNHNGRYLSDL
eeecccccccccceeeeeecccceeeecccccceeccccccccccccccceeeeeeeeeeecccccchhhhh
QALDLKSLTWSKIDAKFQAGSTDSSKSAQVSSCAGHSLISWGNKFFSVAGHTKDPSENITVKEFDPHTCT
hhhhhhhhchhhhhhhhccccccccccceeeecccceeecccceeeeeccccccccccceeeeeecccccce
WSIVKTYGKPPVSRGGQSVTLVGTTLVLFGGEDAKRCLLNDLHILDLETMTWDDVDAIGTPPPRSDHAAA
eeeeecccccccccccceeeeeceeeeeeecccchhhhhhhccchhhhhhhcccccccccccccccccchh
CHADRYLLIFGGGSHATCFNDLHVLDLQTMEWSRPKQQGLAPSPRAGHAGATVGENWYIVGGGNNKSGVS
hhhhheeeeeccccceeccccceeeechhhhhhhcccccccccccccccccceeccceeeeeccccccccc
ETLVLNMSTLTWSVVSSVEGRVPLASEGMTLVHSNYNGDDYLISFGGYNGRYSNEVFALKLTLKSDLQSK
eeeeeeecceeeeeeecccccccceecccceeeeeecccccceeeeccccccccccchhhhhhhhhhhhhhhc
TKEHASDGTSSVLEPEVELSHDGKIREIAMDSADSDLKKDDANELLVALKAEKEELEAALNREQVQTIQL
cccccccccccccccccccccccchhhhhhhhhhhhhhhhhhhhhhhhhhhhhhhhhhhhhhhhhhhhhhh
KEEIAEAEARNAELTKELQTVRGQLAAEQSRCFKLEVDVAELRQKLQSMDALEREVELLRRQKAASEQAA
hhhhhhhhhhhhhhhhhhhhhhhhhhhhhhhhhhhhhhhhhhhhhhhhhhhhhhhhhhhhhhhhhhhhhh
LEAKQRQSSSGMWGWLVGTPPDKSES
hhhhhhhcccceeeeeeccccccceec

Sequence length :   656

GOR4 :
   Alpha helix      (Hh) :   230 is  35.06%
   3_10 helix       (Gg) :     0 is   0.00%
   Pi helix         (Ii) :     0 is   0.00%
   Beta bridge      (Bb) :     0 is   0.00%
   Extended strand  (Ee) :   132 is  20.12%
   Beta turn        (Tt) :     0 is   0.00%
   Bend region      (Ss) :     0 is   0.00%
   Random coil      (Cc) :   294 is  44.82%
   Ambiguous states (?)  :     0 is   0.00%
   Other states          :     0 is   0.00%
```

AFZ62126_K_Vf

```
         10        20        30        40        50        60        70
          |         |         |         |         |         |         |
MAMARASSGLAYPERFYAAAAYAGFDGSPNSTNTVSSKFQNDTALLLYALYQQATVGPCKTPKPSTWNAV
cccccccccccchhhhhhhhhhccccccccccceeeccccchhhhhhhhhhcccccccccccccccceeee
EQSKWKSWNGLANMASTEAMRLFVKILEEEDPGWYSRASNFVLEPVVDMQMNHNPKADPVVENGNSFPET
ehhhhcccccccchhhhhhhhhhhheeecccccccccccceeeechhhhhhcccccccceeecccccccc
KTISSENGTVIETQDKDVVSEGLGSVAVYDQWIAPPISGQCPKARYEHGAVVIQDQMYIYGGNHNGRYLN
eeeecccccceeeecccceeeccceeeeeecceeecccccccccccccccceeeeeeeeeeeccccccccc
DLHVLDLRSWTWSKVDAKVEAESDESKSPATVTPCAGHSLIPWENKLLSIAGHTKDPSESIQVKAFDPQN
ccceeeeeccceeeechhhhhhccccccccceeeccccccccchhhheeeecccccccccchhhhhhccccc
RSWSTLKTYGKAPVSRGGQSVTLVGTNLVIFGGQDAKRSLLNDLHILDLETMTWDEIDAVGGPPSPRSDH
ccceeeeccccccccccccceeeeecceeeeeecccchhhhhhhhhhhhhhhhcceeeeccccccccccchh
AAAVHAERYLLIFGGGSHATCFNDLHVLDLQAMEWTRPIQQGEIPSPRAGHAGVTVGENWFIVGGGDNKS
hhhhhhhhheeeecccccceeccchhhhhhhhhhhhcceeccccccccccccccceeeccceeeeecccccc
GVSETVVLNMSTLVWSVVTSVEGRVPLASEGLSLVVSSYNGEDILVSFGGYNGRYSNDVNVLKPSHKSTK
cceeeeeeecceeeeeeeeeccccccccccccceeeeecccccceeeeecccccccccccceeccccccccc
IMETPVLDSVSAVHNATNATRDLESEFDQEGKIREIVMDNVDAEPMKSKGEVSSEHRIASLKGEKEELES
eeeeceecceeeeccccccchhhhhhhhhhceeeeeeeeccccccccccccccchhhhhhhhhcchhhhhh
SLSKEKLQTLQLKQELAEAETRNTDLYKELQSVRGQLAAEQSRCFKLEVDVAELRQKFQTMEALQKELEL
hhhhhhhhhhhhhhhhhhhhhhhhhhhhhhhhhhhhhhhhhhhhhhhhhhhhhhhhhhhhhhhhhhhhhh
LQRQKAASEQALSAKQRQGSGGVWGWLAGSPGSEREDEA
hhhhhhhhhhhhhhhhhccccceeeeeeccccccccccee
```

Sequence length : 669

GOR4 :

Alpha helix	(Hh)	:	209 is	31.24%
3_{10} helix	(Gg)	:	0 is	0.00%
Pi helix	(Ii)	:	0 is	0.00%
Beta bridge	(Bb)	:	0 is	0.00%
Extended strand	(Ee)	:	153 is	22.87%
Beta turn	(Tt)	:	0 is	0.00%
Bend region	(Ss)	:	0 is	0.00%
Random coil	(Cc)	:	307 is	45.89%
Ambiguous states	(?)	:	0 is	0.00%
Other states		:	0 is	0.00%

AQK61749_K_Zm

```
        10        20        30        40        50        60        70
         |         |         |         |         |         |         |
MGGKQLNIPRASATSAGSVKRRPRRDEASVGSPSPSLESLPPDLGASSPCRLTLQYIGEEEELRFGRVNS
cccccccccccccccccccccccccccccccccccccccccccccccccchhhhhccchhhhhhhccccc
TRRLAPVDRREGEIRAEIQGAAPAMASSGLAYPDRFYAAAAYAGFGVGEATSSAAISRFQNDVALLLYGL
cccccchhhhhhhhhhhhhhhchhhhhccccccchhhhhhhhccccccccchhhhhhhhhhhhhhhhhhh
HQQATVGPCNVPKPRAWNPVEQSKWTSWHGLGSMPSAEAMRLFVKILEEEDPGWYSRAPEFNPEPVVDIQ
cccccccccccccccccccceecceeeeeecccccchhhhhhhhhheeeccccccccccccccccchhhhh
MHKPKDEPHSVPGSTNGTSIPEPNISENGSSVEAQDKDVILEGLSTVSSHDQWTPLSVSGLRPKPRYEHG
ccccccccceeccccccccccccccccccccchhhhchhhhhhccceeecccccccceeccccccccccccc
ATVLQNKMYIFGGNHNGRYLSDLQALDLKSLTWSKVDAKLQAEPADSTKTTQIAPCAGHSLISWGNKFLS
ceeeeeeeeeeeccccccchhhhhhhhhhhhhchhhhhhhhhhcccccccceeeecccccceeecccceeee
IAGHTKDPSEGVTVKEFDPHTCTWSIVRTYGKPPVSRGGQTVTLVGTTLVLFGGEDAERCLLNDLHILDL
eccccccccccceeeeecccccceeeeeecccccccccccceeeeeceeeeeeecccchhhhhhhccchhhh
ETMTWDDVDAIGTPPSPRSDHAAACHADRYLLIFGGGSHATCFNDLHVLDLQTMEWSRPKQQGPTPSPRA
hhhcccccccccccccccccchhhhhhhheeeeecccccceecccceeeechhhhhhcccccccccccccc
GHAGGTVGENWYIVGGGNNKSVACDLLCIFMPNITHTLASTGVSETLVLNMSTLTWSVVSTVEGRVPLAS
cccccccccceeeecccccccccceeeeeecccccccceeecccccceeeeeeecceeeeeeecccccccccc
EGMTLVHSNYGGYDYLISFGGYNGRYNNEVYTLKLSLKSDSQSTLKEETLSDTTSRVIEPEAEIYQDGKI
ccceeeeecccccceeeeeecccccccccccceeeeeeeccccccchhhhcccccccccceccchhhhhhcccc
REISVDNADSDLNNRNDEASEQLLADLKAQKEELEATLSREQLQTVQLKEDIARAETRNAELTKANHLSL
eeeeecccccccccchhhhhhhhhhhhhhhhhhhhhhhhhhhhhhhhhhhhhhhhhhhhhhhhhhhhhhhh
TLNFKLSVANLLLSSQDVSNLRYTLVDVAELRQKLQSMDALEKEVELLRRQRAASEQAAMDAKQRQSSGG
hhhhhhhhhhhhhccccccchhhhhhhhhhhhhhhhhhhhhhhhhhhhhhhhhhhhhhhhhhhhhhhhcccc
MWGWLAGSPTPAV
eeeeeecccccee

Sequence length :   783

GOR4 :
   Alpha helix     (Hh) :   258 is  32.95%
   3₁₀ helix       (Gg) :     0 is   0.00%
   Pi helix        (Ii) :     0 is   0.00%
   Beta bridge     (Bb) :     0 is   0.00%
   Extended strand (Ee) :   132 is  16.86%
   Beta turn       (Tt) :     0 is   0.00%
   Bend region     (Ss) :     0 is   0.00%
   Random coil     (Cc) :   393 is  50.19%
   Ambiguous states (?)  :     0 is   0.00%
   Other states          :     0 is   0.00%
```

Supplementary figure 4 Secondary structure of ACBP in oil crops. The prediction was made using GOR version IV (Garnier et al., 1996; Combet et al., 2000).
