# Supplementary material for: Functional and Structural Diversity of Acyl-coA Binding Proteins in Oil Crops
Source: Front Genet. 2018 May 22;9:182. doi: 10.3389/fgene.2018.00182 (PMC5972291; doi:10.3389/fgene.2018.00182)
Supplement: Supplementary Figure 5 — Hypothetical 3D domain structure of ACBP in oil crops. The model were obtained from Phyre2. A part but not full structure of ACBP could be modeled. Conserved domain were highlighted using VAST. ACBD are in pink, ankyrin repeats are in blue, kelch motifs are in blue, brown, and green, unknown domain is highlighted in gray. A1 to A9 indicate different model of ankyrin repeats ACBP: A1 (O. sativa), A2 (Z. mays), A3 (A. thaliana), A4 (B. rapa and O. europeae), A5 (B. napus), A6 (G. max), A7 (J. curcas), A8 (G. hirsutum), A9 (B. oleraceae and H. annuus). K1–K4 indicate different model of kelch motif ACBP: K1 (Z. mays), K2 (G. max), K3 (A. thaliana, B. napus, B. oleraceae, G. hirsutum, H. annuus, J. curcas, O. europeae, O. sativa, and V. fordii), K4(B. rapa). Models were classified according to the number of domains and number of helixes or strands found in oil crops ACBP. [file Image_5.PDF]

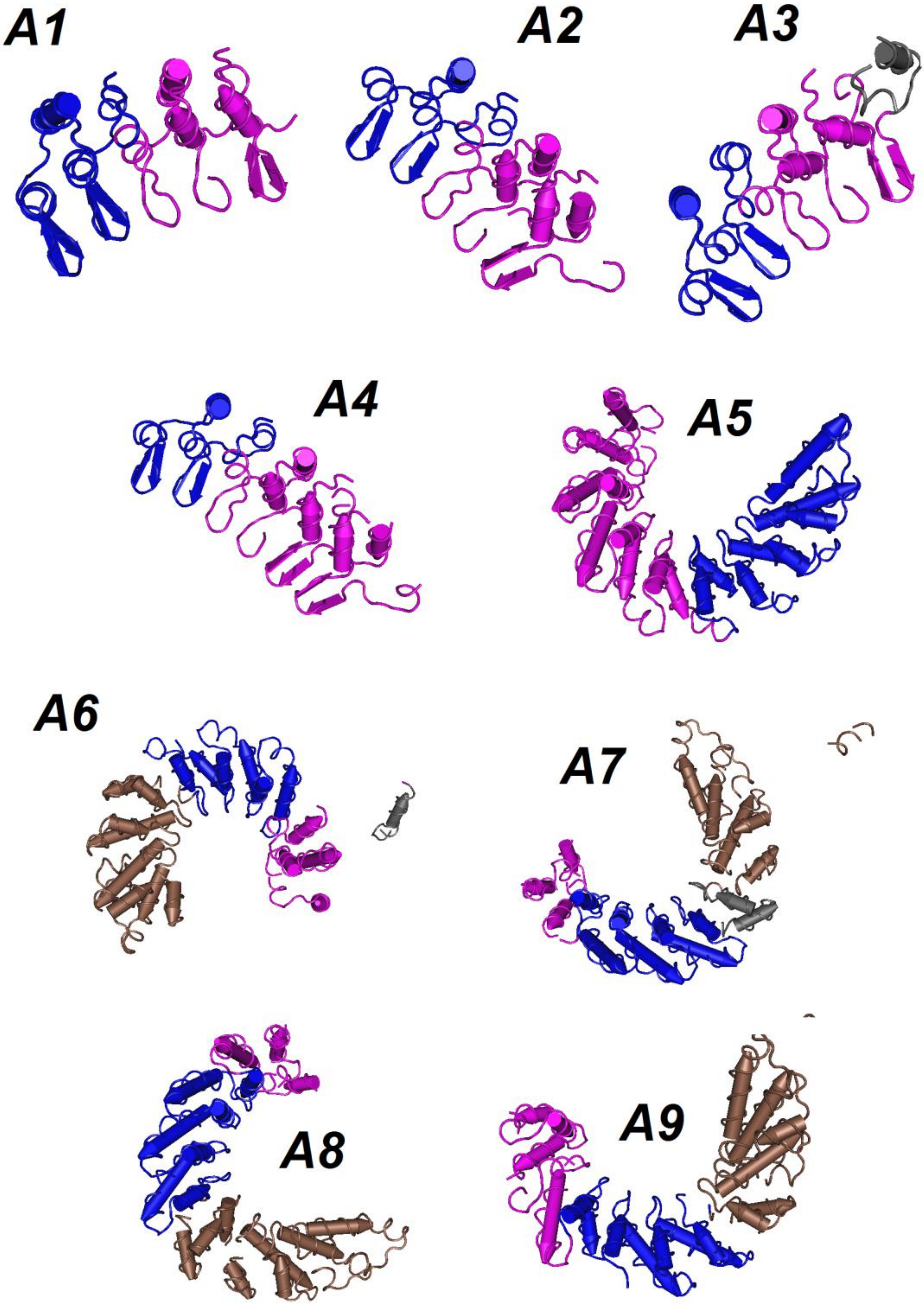
A1
A2
A3
A4
A5
A6
A7
A8
A9

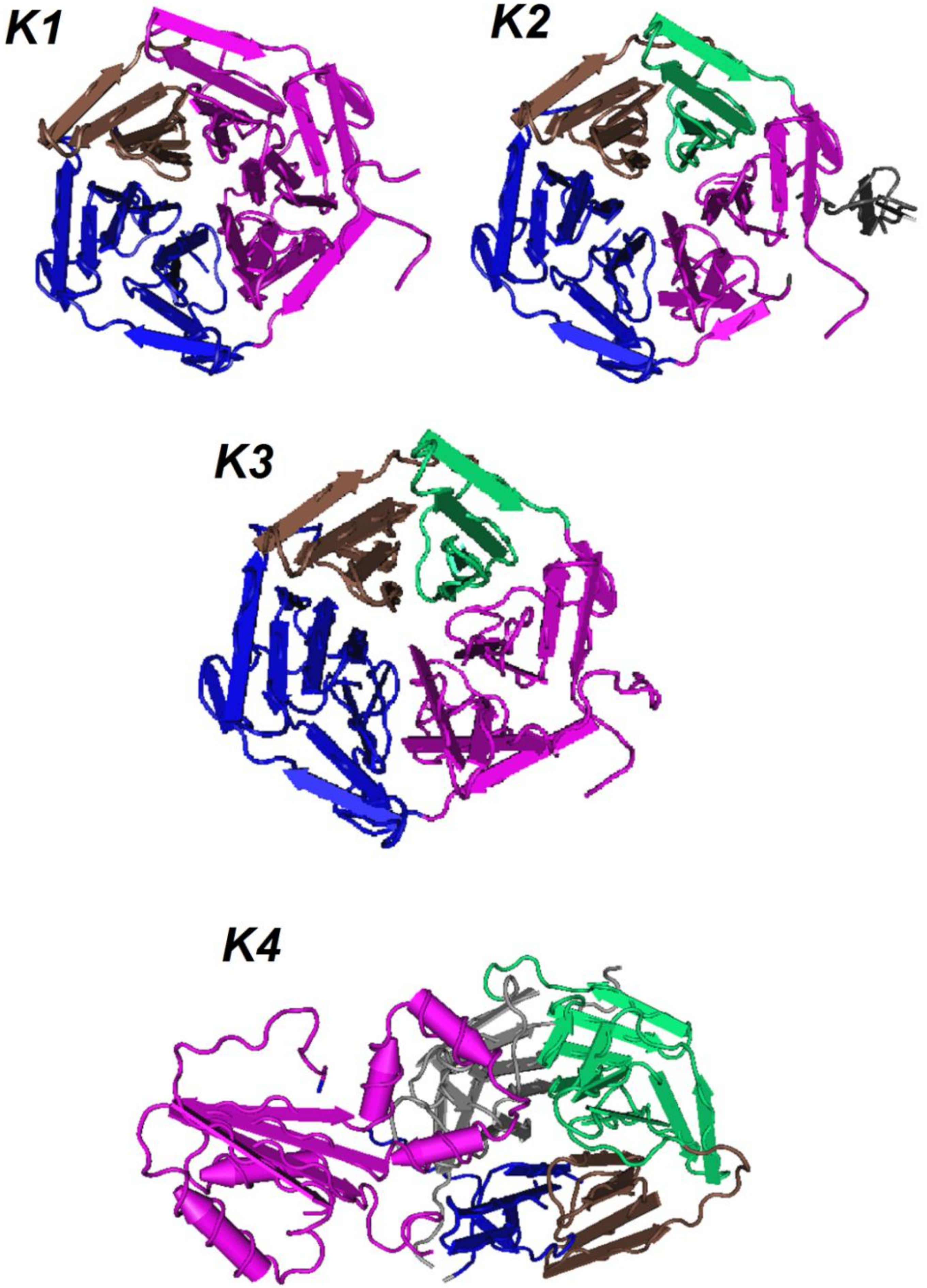
K1
K2
K3
K4

Supplementary figure 5 Hypothetical 3D domain structure of ACBP in oil crops. The model were obtained from Phyre2. A part but not full structure of ACBP could be modeled. Conserved domain were highlighted using VAST. ACBD are in pink, ankyrin repeats are in blue, kelch motifs are in blue, brown and green, unknown domain is highlighted in gray. A1 to A9 indicate different model of ankyrin repeats ACBP: A1 (*O. sativa*), A2 (*Z. mays*), A3 (*A. thaliana*), A4 (*B. rapa* and *O. europeae*), A5 (*B. napus*), A6 (*G. max*), A7 (*J. curcas*), A8 (*G. hirsutum*), A9 (*B. oleraceae* and *H. annuus*). K1 to K4 indicate different model of kelch motif ACBP: K1 (*Z. mays*), K2 (*G. max*), K3 (*A. thaliana, B. napus, B. oleraceae, G. hirsutum, H. annuus, J. curcas, O. europeae, O. sativa,* and *V. fordii*), K4 (*B. rapa*). Models were classified according to the number of domains and number of helixes or strands found in oil crops ACBP..
